# Examining Differences in Fear Learning in Patients With Obsessive-Compulsive Disorder With Pupillometry, Startle Electromyography and Skin Conductance Responses

**DOI:** 10.3389/fpsyt.2021.730742

**Published:** 2021-10-01

**Authors:** Dorothee Pöhlchen, Marthe Priouret, Miriam S. Kraft, Florian P. Binder, Deniz A. Gürsel, Götz Berberich, BeCOME working group, E. B. Binder, Kathrin Koch, Victor I. Spoormaker

**Affiliations:** ^1^Department of Translational Research in Psychiatry, Max Planck Institute of Psychiatry, Munich, Germany; ^2^International Max Planck Research School for Translational Psychiatry, Max Planck Institute of Psychiatry, Munich, Germany; ^3^Department of Neuroradiology, School of Medicine, Klinikum Rechts der Isar, Technical University of Munich, Munich, Germany; ^4^TUM-Neuroimaging Center (TUM-NIC), Technical University of Munich, Munich, Germany; ^5^Windach Institute and Hospital of Neurobehavioural Research and Therapy, Windach, Germany; ^6^Max Planck Institute of Psychiatry, Munich, Germany

**Keywords:** obsessive compulsive disorder, anxiety, inhibitory learning, extinction, fear conditioning

## Abstract

Obsessive-compulsive disorder (OCD) is characterized by recurrent, persistent thoughts and repetitive behaviors causing stress and anxiety. In the associative learning model of OCD, mechanisms of fear extinction are supposed to partly underlie symptom development, maintenance and treatment of OCD, proposing that OCD patients suffer from rigid memory associations and inhibitory learning deficits. To test these assumptions, previous studies have used skin conductance and subjective ratings as readouts in fear conditioning paradigms, finding impaired fear extinction learning, impaired fear extinction recall or no differences between individuals with OCD and healthy controls. Against this heterogeneous background, we tested fear acquisition and extinction in 37 OCD patients and 56 healthy controls, employing skin conductance as well as pupillometry and startle electromyography. Extinction recall was also included in a subsample. We did not observe differences between groups in any of the task phases, except a trend toward higher startle amplitudes during extinction for OCD. Overall, sensitive readouts such as pupillometry and startle responses did not provide evidence for moderate-to-large inhibitory learning deficits using classical fear conditioning, challenging the assumption of generically impaired extinction learning and memory in OCD.

## Introduction

Obsessive-compulsive disorder (OCD) is an anxiety related disorder characterized by persistent, intrusive and undesired thoughts (obsessions) that result in high anxiety and distress as well as compulsive behaviors (1). Before gaining its own chapter in the fifth version of the diagnostic and statistical manual of mental disorders (DSM-5), OCD used to be classified among the section of anxiety disorders. Indeed, fear and anxiety are core characteristics of OCD and key features of the symptomatology, typically preceding and accompanying obsessions and compulsions (2).

A key question in OCD research is to determine the factors that underlie the development and maintenance of the rigid associations between certain triggering stimuli and obsessions and compulsions. One experimental model to assess the creation and maintenance of such pathological associative memories is classical fear-conditioning. This paradigm is used to investigate the learned association between a neutral stimulus and an inherently aversive unconditioned stimulus (US). Over repetition and time, the neutral stimulus becomes a conditioned stimulus (CS+). This stimulus elicits a physiological conditioned response (CR) that can be quantified as an increase in the skin conductance response (SCR), larger pupil dilations and a potentiated startle reflex compared to previous trials or control conditions. A common experimental manipulation in fear conditioning studies is extinction - the creation of a new association between the CS and safety due to repeated presentations of the CS without the US. Fear extinction can be conceptualized as a conflict between two opposing knowledge states, comprising the original acquisition of an excitatory association between the CS and the US and the new, inhibitory learning, decreasing the CR to the CS (3).

Fear conditioning and extinction protocols are commonly employed to study anxiety disorders but have also been used to study and model processes related to the development and maintenance of OCD. One of the first studies to explicitly compare OCD and healthy controls (HC) in their physiological and neural correlates during fear conditioning and extinction was published by Milad et al. (4). While there were no differences in SCR between the 21 OCD patients and the 21 HC during conditioning and extinction, they observed that OCD patients exhibited deficits in recalling extinction memory the next day. Furthermore, functional magnetic resonance imaging (fMRI) revealed that the OCD group was characterized by having significantly less activity in the hippocampus/caudate nucleus and ventromedial prefrontal cortex (vmPFC) during conditioning and extinction respectively, which is in line with inhibitory processing deficits. While the results in SCR were essentially replicated by McLaughlin et al. (5), other groups showed extinction learning deficits in adolescents with OCD (6, 7), or reported a complete lack of differences (8, 9). Apergis-Schoute et al. (10) compared SCR to a first conditioning phase and a later reversal phase, where the former CS+ was no longer paired with the US and the former CS- was paired with the US instead. In their study, the OCD group had generally lower SCR toward the CS+ after reversal but did not show differential SCR, which was interpreted as impaired safety signaling in OCD patients. Overall, the few existing studies are heterogeneous and insufficient to draw compelling evidence for inhibitory learning or extinction recall deficits in OCD.

Against this background, it seems promising to include other readouts to quantify psychophysiological responses in a fear conditioning paradigm, potentially exposing dysfunctional mechanisms in OCD. To our knowledge, no previous study has used pupillometry as a readout of fear conditioning in OCD before. Pupil dilations reliably discriminate between CS- and CS+ (11–15) and unlike SCR appear to show less habituation over time (16). Similarly, scant attention has been given to startle electromyography (EMG) as a readout in OCD. Compared to SCR and pupil dilations, startle responses are not only modulated by arousal, but also by the valence of the presented stimulus, offering additional insights into emotional processing (17). Moreover, there is evidence suggesting that extinction deficits in another disorder, posttraumatic stress disorder, are more readily observed in SCR when probing extinction recall (18) but are visible already during immediate extinction with startle EMG (19, 20). If this is a methodological issue, for instance due to dissimilar habituation effects in the readouts, then something similar could be occurring within OCD.

In our study, we therefore aimed at investigating potential differences between 37 OCD patients and 56 HC in a classical fear-conditioning task, employing pupil dilations, startle amplitudes and SCR across conditioning and extinction (day 1). In a subset of participants, we additionally examined extinction recall and reinstatement (day 2). We hypothesized the diagnosis of OCD to be reflected in altered extinction following a fear conditioning phase. Since startle EMG and especially pupillometry are not as strongly affected by habituation as SCR, these readouts would be more sensitive during extinction and provide more evidence on whether group differences exist during that phase already or may only appear when probing extinction memory during the extinction recall phase.

## Methods

### Participants

We combined data from two samples undergoing the same task and a similar experimental procedure. The first sample (from now on sample A, Table 1) originally contained 24 HC (12 females, aged 33.1 ± 11.2, M ± SD) who were recruited through online advertisements and 28 treatment-seeking OCD patients (14 females, aged 34.4 ± 12.7, M ± SD) who were recruited from clinics specialized on OCD in the larger Munich area (psychosomatic clinic Windach and day clinic Munich Westend). Participants took part in a study on the neurobiology and psychophysiology of impaired fear learning and extinction processes in OCD that was approved by a local ethics committee in accordance with the declaration of Helsinki. Written informed consent was obtained from all participants. All participants were reimbursed for participation. Patients were considered to be eligible if they met ICD-10 criteria for OCD, scoring at least 15 points on the Yale-Brown Obsessive Compulsive Scale (Y-BOCS) (21). Apart from the Y-BOCS, participants completed the Obsessive Compulsive Inventory-Revised (OCI-R) (22), the Hamilton Anxiety Rating Scale (HAM-A) (23) and the Hamilton Depression Rating Scale (HAM-D) (24). Of the original sample, only a subset took part in the fear-conditioning experiment (17 HC: 7 females, aged 30.0 ± 6.1; 9 OCD: 4 females, aged 27.9 ± 14.4, M ± SD). Two of the HC were excluded (one male, one female) due to missing data, resulting in a subsample of 15 HC and nine patients with OCD (age of onset 15 ± 8.2). Five patients were on stable medication (>8 weeks), with three patients taking serotonin-reuptake inhibitors, one patient taking amphetamines and one patient taking serotonin-reuptake inhibitors and neuroleptics. Participants filled in a self-report questionnaire on comorbidities with one patient reporting a comorbid depression. Participants suffering from PTSD, bipolar disorder, substance abuse or schizoaffective disorder were excluded from the study.

**Table 1:** Demographic and psychometric information of OCD and healthy controls in the combined sample and sample A.

**Combined sample**	**OCD (*n* = 37)**			**HC (*n* = 56)**				
**Variable**	**N**	**M**	**SD**	**N**	**M**	**SD**	**t/U/X2**	***P***
Age	37	32.6	11.0	55	31.9	9.3	−0.39^a^	0.70
Female	22			32			0.03^b^	0.86
**Sample A**	**OCD (*n* = 9)**			**HC (*n* = 15)**				
Variable	N	M	SD	N	M	SD	t/U/X2	*P*
Y-BOCS	9	19.8	3.6	13	0.8	2.5	−14.05^c^	<0.001
OCI-R	9	30.3	7.4	11	1.2	1.4	−12.9^c^	<0.001
Medication	5 (3 SSRI, 1 Amphetamine, 1 SSRI and neuroleptics)							

a *t-Test,*

b *Mann-Whitney-U-Test,*

c *X2 test*.

The second sample (from now on sample B, Table 1) consists of participants taking part in the ongoing BeCOME study (registered on ClinicalTrials.gov, TRN: NCT03984084) (25). Data collection threshold for the analyses presented here was in August 2020. Analysis on previous batches reporting results of the fear-conditioning task have been published in (25, data collection threshhold June 2017) and (26, data collection threshhold October 2019). All participants provided their written informed consent after the study protocol had been fully explained and were reimbursed for their participation. Participants in the BeCOME study undergo a computer-based slightly modified version of the Munich-Composite International Diagnostic Interview (26) and were assigned to the control group when they did not fulfill criteria for any current or past lifetime, full or subthreshold psychiatric diagnosis and existing physiological datasets (*n* = 41, 25 females, aged 32.6 ± 10.2, M ± SD). Participants fulfilling full criteria for a current OCD were assigned to the OCD group (*n* = 28, 18 females, aged 34.0 ± 10.0, M ± SD, see Table 2 for a comorbidity table). All participants from sample B were unmedicated.

**Table 2:** Comorbidity table displaying current and lifetime comorbidities of the OCD group in sample B.

	**Current**		**Lifetime**	
	***n***	**%**	***n***	**%**
Specific phobia	17	60.1	6	21.4
Phobia	20	71.4	8	28.6
Panic disorder	8	28.6	2	7.1
PTSD	6	21.4	4	14.3
Any depressive disorder	22	78.6	1	3.6
Dysthymia	13	46.4	0	0
Bipolar disorder	4	14.3	1	3.6

### Experimental Setup and Measurements

#### Task

**Figure 1:**
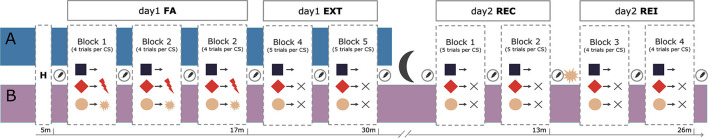
Experimental procedure of the fear-conditioning task. Sample **(A)** underwent a habituation phase, three blocks of fear acquisition (FA) and two blocks of extinction (EXT) on day 1. Sample **(B)** additionally underwent a two-block fear recall (REC) and two-block reinstatement (REI) procedure on day 2. All blocks were separated by US-expectancy ratings.

Both samples underwent an uninstructed classical fear-conditioning task (Figures 1A,B) consisting of a habituation, a conditioning and an extinction phase on one day (day 1). The fear conditioning and extinction task was divided into five blocks separated by US expectancy ratings. The fear conditioning phase consisted of three blocks where participants learnt to associate three distinct CS with either no aversive outcome, an electrical shock, or an airblast. In each block, all three CS (CS-, CS+ shock, and CS+ air) were presented four times in a pseudo-randomized order. Reinforcement rates for both CS+ was 75% and startle probes occurred in 75% of trials and in 40% of inter-trial-intervals (ITIs). The extinction phase directly followed upon conditioning and comprised two blocks. Here, the CS+ air was not presented anymore and the CS- as well as the CS+ shock were shown five times each per block. No US occurred during extinction, but startle probes were delivered in 60% of trials and ITIs. Sample B additionally underwent a recall and return of fear procedure on the following day. Sample A underwent fear conditioning and extinction between 2 and 4 PM while sample B was tested between 10 AM and 12 PM.

#### Stimuli

Three differently colored geometrical shapes served as neutral stimuli, two of which were followed by distinct US. Stimuli were presented for ~4 s in equal brightness in the center of a dark screen and were followed by an ITI varying between 10 s and 14 s, during which a fixation cross was presented. The first US consisted of a mild electrical shock to the right wrist delivered for 20ms using a Linear Isolated Stimulator (Stimsola, BIOPAC Systems, Inc., Goleta, USA) via two Ag/AgCl electrodes filled with electrolyte gel. The intensity of the electric stimulation was adjusted individually for each participant. Intensity was increased in 0.5 mA steps from 0.5 mA until the participant rated the shock as “very unpleasant but not yet painful”. The second US consisted of a 9 bar airblast lasting 250ms, equal for each subject. This US has been shown to be especially useful in conditioning paradigms used to distinguish between psychiatric patients and HC (17, 27). The airblast was delivered through an air nozzle attached to a backpack worn on the chest and placed in a distance of approximately 1-2 cm to the larynx. Startle probes consisted of a 108 dB burst of white noise with a near instantaneous rise time and were delivered binaurally through headphones, lasting 40ms (27). They were delivered approximately 3 seconds after stimulus onset. For a more detailed description of procedure and experimental setup please refer to (28, 29).

### Physiological Recordings and Preprocessing of Physiological Readouts

For measuring SCR, two 5mm Ag/AgCl electrodes filled with electrolyte gel were attached to the palm of the left hand. EMG was measured via two 5mm Ag/AgCl electrodes that were filled with electrolyte gel and placed on the orbicularis oculi muscle underneath the left eye as well as behind the left ear as a reference electrode. Impedance of the electrodes was below 5–10 kΩ. SCR and EMG signal were recorded with 2,000 Hz sampling rate via a wireless system (BIOPAC Systems, Inc., Goleta, USA) and AcqKnowledge Software (Version 4.4.0, BIOPAC Systems, Inc., 2014). Pupil dilations were acquired with an EyeLink 1000 desktop system (SR Research Ltd., Ottawa, Canada). Participants rested their chin on a head-rest that was placed in a distance of 80 cm to the screen. The camera recording the pupil diameter of the right eye was located underneath the screen at a distance of approximately 60cm to the eyes of the participant. A standard nine-point-calibration was conducted before starting the measurement. Pupillometry was recorded at a sampling rate of 250 Hz.

Skin conductance data were lowpass filtered at 1 Hz in AcqKnowledge. All further preprocessing and analysis steps were performed in Matlab (version R2020b, MathWorks, Natick, USA). After downsampling from 2000Hz to 1000 Hz, SCR were segmented around trials (500 ms before CS onset to 4 s after CS onset). To quantify SCR, the peak skin conductance between 2 s and 4 s after CS onset and the minimum skin conductance between 1 s after CS onset and the time of the peak were determined. Amplitudes were defined by subtracting the minimum SCR from the peak SCR. Trials where SCR was only declining were scored as zero and included in the analysis. Trials were excluded when the standard deviation (SD) of a segment deviated by more than 3.3 SD from the mean SD across all trials (3.5 ± 3.5%, M ± SD).

Pupil data was processed and analyzed in Matlab. Missing data resulting from blinks were linearly interpolated between the last saccade before blink onset and the last saccade after blink offset. Saccade markers were provided by EyeLink software (SR research Ltd). Interpolated data were smoothed with a sliding window of 400 ms. Trial wise pupil dilations were calculated by subtracting the maximum pupil dilation within 2–4 s after stimulus onset from the average pupil size during a 500 ms baseline period preceding stimulus onset. Pupil size and gaze coordinates were segmented around trials (500 ms before CS onset to 4 s after CS onset). The automatic artifact detection was performed aiming at excluding trials with too many missing values, artifacts, or outlier trials. First, trials with >50% interpolated data points were disregarded. Second, we identified trials with sudden jumps in pupil size. For this purpose, we split each trial into five subsegments (500 ms baseline and four 1 s segments covering the stimulus presentation) and calculated the SD for these subsegments across all trials per participant. Trials deviating by more than 3.3 SD from the participant's average deviation in any of the sub-segments were excluded. Third, trials where the gaze was not directed at the center of the screen for more than 500 ms were excluded. To do so, we defined a cut-off window around the participant's median gaze position across all trials. The limits of this window were informed by the mean gaze deviation across all participants. Exclusion criteria partially overlapped and resulted in 13.5 ± 17.6%, M ± SD of excluded trials.

EMG data was band-pass filtered between 28 and 400 Hz, rectified and lowpass filtered at 40 Hz (30) in AcqKnowledge. All further steps were done in MATLAB. Startle data was downsampled to 1,000 Hz and segmented around startle probes (50 ms baseline before startle onset and 200 ms after startle offset). The startle response was quantified as the amplitude between the baseline and the maximum response in a window of interest from 20 to 120 ms after the startle probe. Trials were excluded if the SD or maximum during the baseline exceeded the SD or maximum in the window of interest (8.8 ± 11.5%, M ± SD).

To ensure comparability between participants, valid physiological responses were z-transformed over trials within participants across conditioning and extinction for the analysis of the joint sample and across conditioning, extinction, recall and reinstatement for the additional analyses comprising only sample B. Responses of all measurements were averaged across blocks.

### Statistical Analyses

We report Bayesian statistics as implemented in the software package JASP 0.12.2 (https://jasp-stats.org). We performed two-way repeated measures (rm) ANOVAs with stimulus (CS-, CS+ shock, CS+ air, ITI) and time (3 levels during conditioning, 2 levels during all other task phases) as within-subject factors and diagnostic category (OCD versus healthy control) as between-subject factor. Physiological responding and subjective ratings were the dependent variables. In a Bayesian rmANOVA different models are compared based on their likelihood given the data. In our case, model comparisons were done between the null model, stating that there is no effect of time, stimulus, their interaction, or the group factor, and 18 alternative models with either an effect of time, stimulus, group or any two-or three-way interaction. The prior probability is equally distributed over all possible options (0.0526) and the updated probability after observing the data (P(M|data)) provides the relevant output for these analyses. The posterior odds represent the relative plausibility of the respective model after observing the data, and it is equal to the Bayes factor (BF10) multiplied by the prior odds. The Bayes factor quantifies the change of relative plausibility given the data. A BF10 of around one indicates that the observed data are equally likely to occur under both models, a BF10 between one and three can be interpreted as anecdotal evidence for the alternative hypothesis. A BF10 above three but under 10 is seen as moderate evidence for the presence of an effect in favor of the alternative model, and a BF10 above 10 is proposed to indicate strong evidence for the presence of an effect. Whereas, for example a BF10 < 1/3, which is mathematically equivalent to BF01 > 3, can be interpreted as moderate evidence in favor of the null model (31). Based on our a-priori hypotheses on extinction or extinction recall deficits in the OCD group we also report independent t-tests comparing differential responding (CS+shock versus CS−) between the OCD and HC group for all measures (SCR, pupillometry, startle, subjective ratings) and task phases of interest (extinction block 1 and 2, recall block 1 and 2). For readability and transparency reasons, we followed a hybrid approach and also report more commonly used frequentist statistics in the supplement (32).

## Results

**Figure 2:**
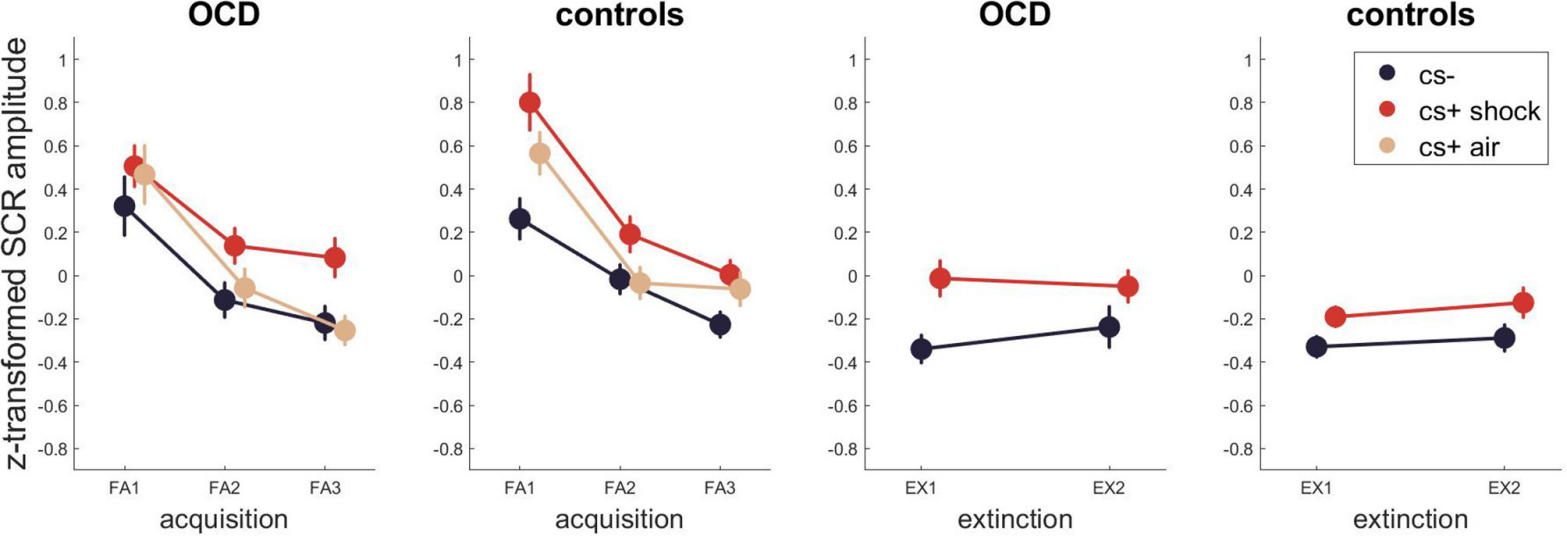
SCR across fear acquisition and extinction. Error bars are standard errors of the mean.

For skin conductance (Figure 2, Tables S1–S3 for Bayesian and Tables S4,S5 for frequentist statistics) the best model for fear acquisition comparing responses to CS- and CS+ shock contained the time and stimulus effect P(M|data) = 0.76. The best model for fear acquisition comparing CS- and CS+ air was also the one with main effects of time and stimulus P(M|data) = 0.39 as well as the model including time P(M|data) = 0.35. For fear extinction, the best model was the model including only stimulus with P(M|data) = 0.55, indicating incomplete extinction across both groups. There was no evidence for models including either a group effect or an interaction with group (stimulus x group, time x group, time x stimulus x group) during any task phase.

**Figure 3:**
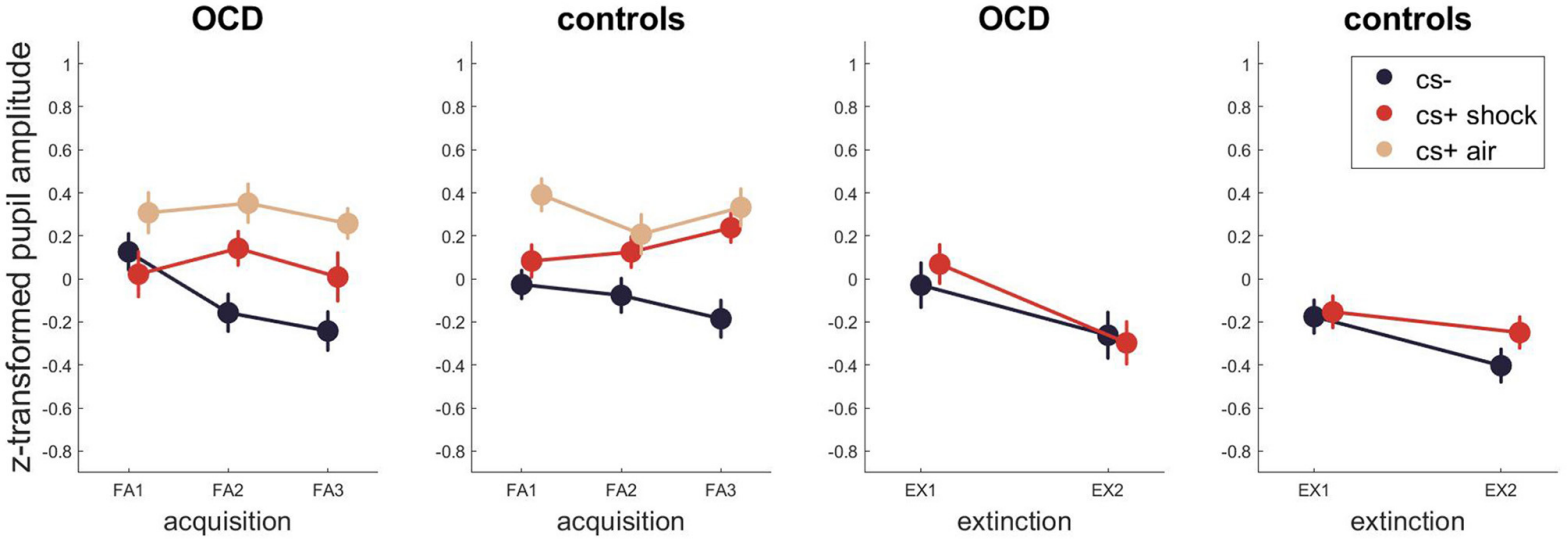
Pupil dilations across fear acquisition and extinction. Error bars are standard errors of the mean.

For pupillometry (Figure 3, Tables S7–S9 for Bayesian and Tables S10–S12 for frequentist statistics) the best model for fear acquisition comparing responses to CS- and CS+ shock contained only the stimulus effect P(M|data) = 0.75. The best models for fear acquisition comparing CS- and CS+ air were the ones containing only stimulus P(M|data) = 0.43 and time as well as stimulus P(M|data) = 0.37. For fear extinction, the best model was the model including only time with P(M|data) = 0.56. There was no evidence for models including either a group effect or an interaction with group (stimulus x group, time x group, time x stimulus x group) during any task phase.

**Figure 4:**
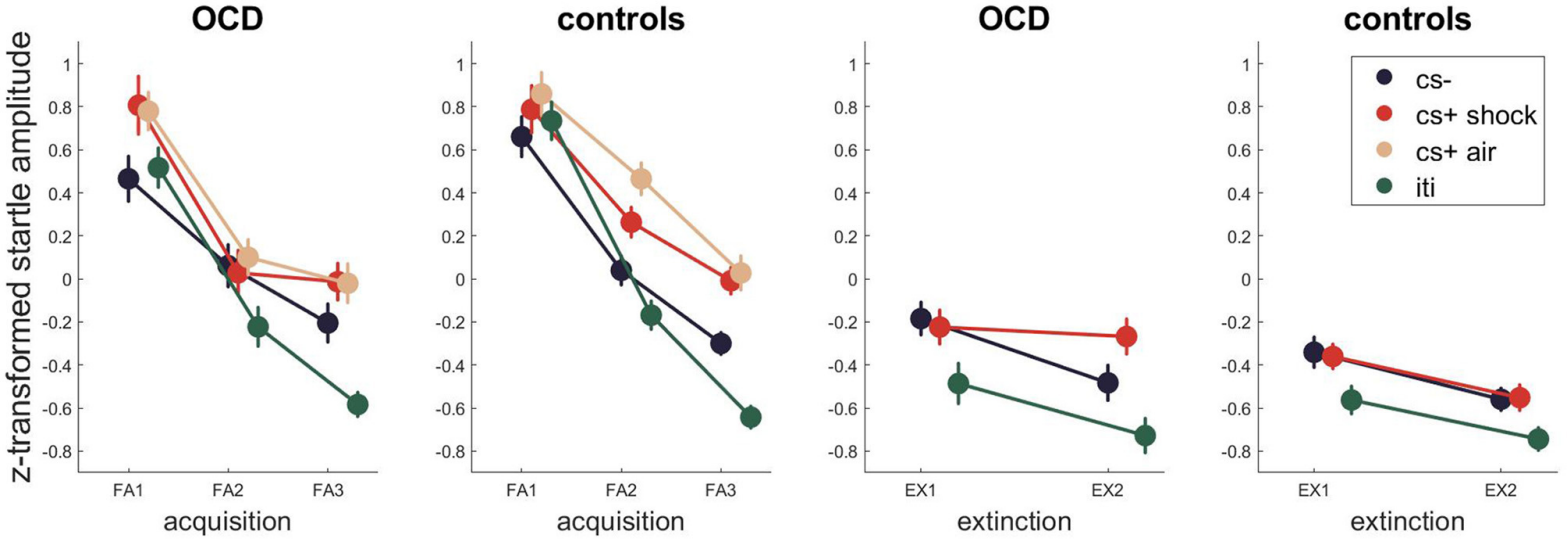
Startle EMG across fear acquisition and extinction. Error bars are standard errors of the mean.

For the startle response (Figure 4, Tables S13–S15 for Bayesian and Tables S16–S18 for frequentist statistics) the best model for fear acquisition comparing responses to CS- and CS+ shock and ITI as well as CS-, CS+ air and ITI contained the time and stimulus effect as well as their interaction P(M|data) = 0.80 and P(M|data) = 0.63. For fear extinction, the winning model included time, stimulus and group P(M|data) = 0.63, indicating different startle amplitudes between the patient and control group.

**Figure 5:**
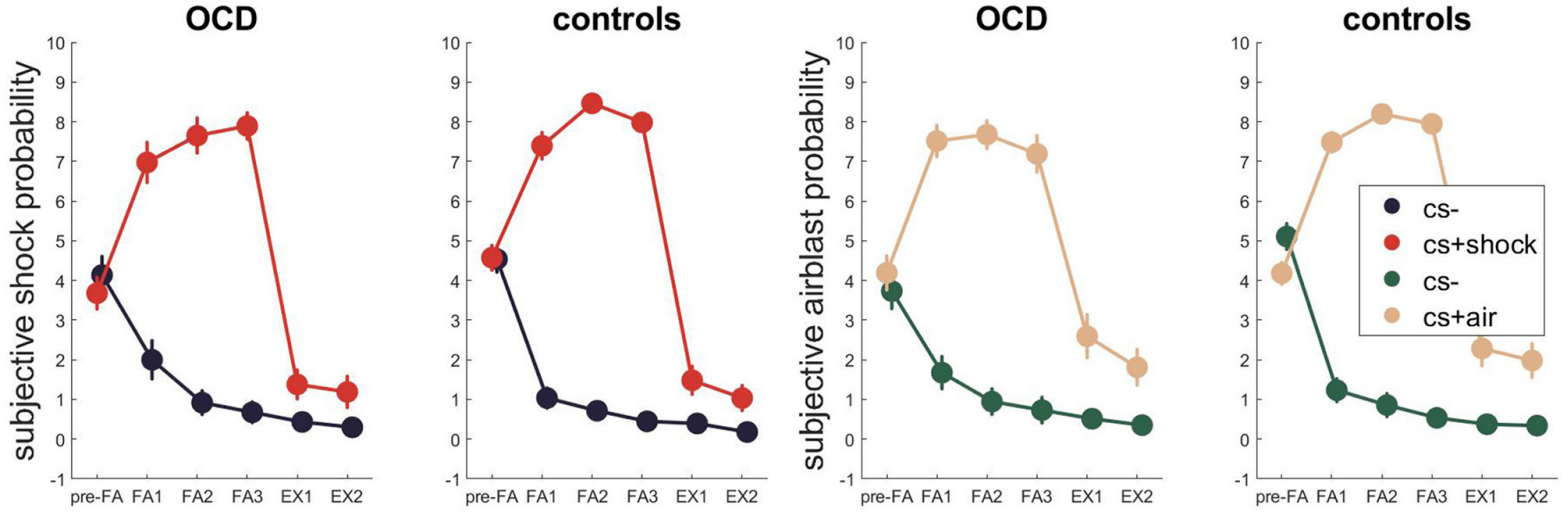
Subjective US expectancy across fear acquisition and extinction. Error bars are standard errors of the mean.

Regarding the subjective ratings (Figure 5, Tables S19–S22 for Bayesian and Tables S23–S26 for frequentist statistics), the best model for fear acquisition comparing subjective shock expectancy to CS- and CS+ shock contained the time and stimulus effect as well as their interaction P(M|data) = 0.65. During fear extinction, the winning models only contained the stimulus effect P(M|data) = 0.46, followed by the model containing the main effects of time and stimulus P(M|data) = 0.26.

When assessing subjective airblast probability for the CS- and CS+ air during conditioning and extinction, a similar picture evolved with the former winning model containing time and stimulus effects as well as their interaction P(M|data) = 0.79 and the latter containing only the main effect of stimulus P(M|data) = 0.58.

For sample B, we additionally had data for fear recall and reinstatement phases, allowing us to investigate whether OCD and HC would differ in extinction recall. None of the Bayesian or frequentist analyses revealed group as a significant factor in contributing to stimulus or time-dependent physiological or subjective responding (Tables S27–S42). *Post-hoc* tests, comparing differential responding of HC and OCD during extinction and recall also did not show evidence for group differences (Table 3).

**Table 3:** Differential physiological responding (CS + shock versus CS-) between OCD and HC during extinction and recall.

	**Extinction block 1**		**Extinction block 2**		**Recall block 1**		**Recall block 2**	
	**BF(10)**	***d***	**BF(10)**	***d***	**BF(10)**	***d***	**BF(10)**	***d***
SCR	0.729	−0.35	0.226	−0.03	0.436	0.281	0.306	−0.165
Pupillometry	0.265	−0.13	0.352	0.22	0.440	−0.285	0.284	−0.108
Startle	0.224	0.03	0.664	−0.34	0.654	−0.369	0.262	0.035
Subjective ratings	0.231	0.06	0.222	−0.02	0.285	0.133	0.258	0.060

## Discussion

In our study, we aimed to investigate potential differences between 37 OCD patients and 56 HC in a classical fear-conditioning task, comparing pupil dilations, startle amplitudes, SCR and subjective US expectancy ratings across conditioning, and extinction. Extinction recall was investigated in a subsample. Based on the current literature positing deficits in inhibitory learning in OCD, we hypothesized a group difference for OCD during fear extinction in physiological or subjective measures [(33), under review]. We found no group differences in SCR, pupillometry or subjective ratings in extinction learning and extinction recall; we only observed subtle differences in startle responses during extinction, with a trend toward higher amplitudes in OCD patients.

Our findings appear to contradict the literature on fear learning in OCD, but a closer look at the studies to date shows a large heterogeneity in the findings. Psychophysiological studies show a rather inconclusive pattern: Milad et al. (4) and McLaughlin et al. (5) observed group differences in SCR during extinction recall but not immediate extinction, Geller et al. (6) and McGuire et al. (7) reported group differences in SCR during immediate extinction in adolescents, Nanbu et al. (8) and Tracy et al. (9) did not observe any differences during extinction in SCR or startle EMG, whereas Kaczkurkin & Lissek (34) revealed increased generalization in startle responses in an OCD trait group, and Apergis-Schoute et al. (10) less differential responding in fear reversal that was already visible during fear conditioning. The findings during extinction recall are also not completely unambiguous: extinction retention was positively correlated with OCD severity in one study (4), but not in a follow-up study (5), in which the interaction was at trend-level significance. So, the picture is not that straightforward, which is why our study reporting no moderate-to-large differences seems to fit well within this large variety of findings.

We hypothesized that between-group differences would emerge in the more sensitive pupillometric and startle measures. While differential pupil responses reflected successful conditioning and extinction in our study, they were similar between OCD and HC. Previous research has shown that CS-elicited pupil dilations largely reflect valence-independent outcome expectancy (16) and are closely related to activity in regions of the salience network (28, 35). Integrating pupil dilations as regular readouts in future fear conditioning research will allow to compare our lack of between-group differences with other studies, potentially informing on similarities or differences in processing salience and aversive outcome expectancy between OCD and controls.

Regarding startle, we observed a trend of the OCD group displaying a higher overall amplitude during extinction than the controls. This trend could be tentatively interpreted as a deficit in extinction (no significant group interaction, but an effect size for the differential extinction d ~ 0.3), but may also be a result of reduced habituation in the OCD group as shown by a main effect of group. In line with the latter explanation, Kumari et al. (36) presented startle responses during negative and neutral movie clips. The OCD group demonstrated larger startle amplitude, independent of time or valence leading the authors to hypothesize a constant hyperexcitability of fear circuits in the OCD group. Another study probed prepulse inhibition of the acoustic startle and found prepulse inhibition deficits in unmedicated OCD patients compared to controls. These results suggest an impaired sensorimotor gating, possibly reflecting sensory hyperarousal in the disorder (37). In the only other study probing startle during fear-conditioning and extinction in OCD, the group scoring higher on OCD scale showed a quicker acquisition of the CR to the CS+ in the higher OCD trait group, but there were also no differences in startle responding during extinction (9).

Finally, we found no group effect in the subjective ratings, showing that patients and controls equally estimated electrical shock and airblast probability after the different CS. This is in line with most other beforementioned studies also not reporting differences in subjective measures, with the exception of Armstrong and Olatunji (34) reporting elevated ratings to CS+ in the higher OCD trait group both during conditioning and extinction. Importantly, the authors used a disgust conditioning approach, that could drive the effects compared to other classical conditioning studies.

Indeed, the absence of evidence, and from a Bayesian perspective, the moderate evidence for absence of associative learning differences between the groups, could be explained by the paradigm employed. Classical conditioning as a general model for the evolvement of pathological associative memories may not be equally well suited for investigating different types of anxiety disorders or OCD. In a meta-analysis of fear conditioning studies, the largest effects are found for disorder-relevant conditioned stimuli (38, 39), questioning whether the disorders arise from generic fear conditioning abnormalities or whether abnormalities in fear-conditioning are primarily visible after the disorder has developed. To capture OCD symptomatology, one way forward would be to adapt the paradigm more specifically to the disorder, for example by using disgust conditioning (applying contamination stimuli as unconditioned stimuli). Multiple studies have found a positive correlation between disgust proneness and OCD, suggesting that disgust processing could play a specific role in the development of the disorder (40). Armstrong and Olatunji (34) compared two groups with high and low contamination concerns using disgust conditioning, and found that the high contamination concern group reported less reduction in US expectancy during extinction. This result suggests that fear of contamination can modulate the adjustment of US expectancy and extinction learning processes. To our knowledge, the physiology of disgust conditioning has not been assessed with classical psychophysiological readouts to date and may represent a promising future research avenue.

Moreover, another potential flaw of our fear conditioning procedure is that it may not be ambiguous enough to capture divergent response patterns in OCD patients (41). The paradigm is quite easy with the majority of participants quickly learning when to expect a shock or an airblast. Cooper and Dunsmoor [(33), under review] point out that such classical conditioning allows to investigate associative learning through automatic sensory associations, but does not capture inductive reasoning and cognitive biases leading to obsessions and compulsions in OCD. Indeed, unlike non-clinical mental intrusions, obsessions in OCD can result not only from simple associations but also from abstracted thoughts that enforce the belief of a potential danger at the view of a trigger (42). A paradigm with less or a more unclear reinforcement would increase the ambiguity of the task and could trigger larger difference between groups. For instance, it was shown with SCR data that OCD patients have troubles in decision making under implicit risk conditions but not under explicit ones (43). In this experiment, patients participated in two gambling games: The Iowa gambling task that has implicit risk rules, and the Game of dice task, with an explicitly stated risk. Interestingly, the SCR significantly differed between the OCD and control groups for the task with implicit risk, but not for the task with explicit risk. A potentiating factor explaining a deficit in extinction learning in OCD could therefore be the intolerance of uncertainty, manifest as an increased need for control, and shown to be a shared personality trait among OCD patients (44).

The main drawbacks of our study are the relatively small sample size, precluding statements on small effect sizes, as well as the lack of the Y-BOCS scale in sample B, making it impossible to compare symptom severity between both subsamples and analyzing the influence of symptom severity on fear and extinction learning in a more continuous manner. Furthermore, subjective or physiological readouts may not be optimal in capturing OCD symptomatology. Instead, existing readouts need to be paired with behavioral readouts that may be better suited to measure compulsive, repetitive behavior and avoidance patterns (45). Illustrating the need for avoidance paradigms, Gillan et al. showed that OCD patients had greater avoidance habits than control subjects (46). However, SCR responses did not differ between groups at any stage during the shock avoidance task. As avoidance behavior is thought to prevent extinction and maintain OCD symptomatology, it would be interesting to test OCD patients in a virtual environment, investigating naturalistic body postures and movement in an operant conditioning task (47).

## Conclusion

To conclude, using a classical fear conditioning paradigm, we compared response patterns of different physiological readouts in a group of OCD patients to HC. We did not observe differences in SCR, pupillometry or subjective ratings, except for small group differences during extinction in startle EMG. Our results add to the existing literature by pointing toward an absence of evidence for large associative fear learning deficits in OCD. Overall, more research is needed to establish a clearer picture of inhibitory learning deficits in OCD. Future work should compare results of classical compared to OCD-specific aversive conditioning and include behavioral readouts to better understand the key mechanisms sustaining the disorder.

## Data Availability Statement

The original contributions presented in the study are included in the article/Supplementary Material, further inquiries can be directed to the corresponding author/s.

## Ethics Statement

The studies involving human participants were reviewed and approved by Ethikkommission of the Ludwig Maximilians University, Munich. The patients/participants provided their written informed consent to participate in this study.

## Author Contributions

DG, KK, and VS designed the study, developed, and implemented the tasks. FB, MK, and DG acquired the data. DP, MK, MP, and VS analyzed and interpreted the data. DP and MP wrote the manuscript under supervision of VS. All authors critically read and approved the submitted version.

## Funding

This study was supported by a Deutsche Forschungsgemeinschaft (DFG) grant to KK (KO 3744/7-1) and VS (SP 1325/2-1).

## Conflict of Interest

VS has received income from consultations and advisory services for Roche. The remaining authors declare that the research was conducted in the absence of any commercial or financial relationships that could be construed as a potential conflict of interest.

## Publisher's Note

All claims expressed in this article are solely those of the authors and do not necessarily represent those of their affiliated organizations, or those of the publisher, the editors and the reviewers. Any product that may be evaluated in this article, or claim that may be made by its manufacturer, is not guaranteed or endorsed by the publisher.

## BeCOME Working Group

Binder, E. B., Brückl, T. M., Czisch, M., Erhardt, A., Grandi, N. C., Lucae, S., Sämann, P., Tontsch A., von Muecke-Heim, I.A., Ziebula, J.
