# Supplementary material for: Examining Differences in Fear Learning in Patients With Obsessive-Compulsive Disorder With Pupillometry, Startle Electromyography and Skin Conductance Responses
Source: Front Psychiatry. 2021 Oct 1;12:730742. doi: 10.3389/fpsyt.2021.730742 (PMC8517251; doi:10.3389/fpsyt.2021.730742)
Supplement: Supplementary file 1 [file Data_Sheet_1.ZIP › supplementary tables S1 - S26.html]

JASP 


# sample A and sample B conditioning and extinction - Tables S1 - S26

## Table S1. SCR conditioning (cs+shock)

| Model Comparison | | | | | | | | | | | |
| --- | --- | --- | --- | --- | --- | --- | --- | --- | --- | --- | --- |
| Models | | P(M) | | P(M|data) | | BF M | | BF 10 | | error % | |
| time + stim |  | 0.053 |  | 0.758 |  | 56.407 |  | 1.000 |  |  |  |
| time + stim + ocd\_vs\_hc |  | 0.053 |  | 0.112 |  | 2.274 |  | 0.148 |  | 4.643 |  |
| time + stim + time  ✻  stim |  | 0.053 |  | 0.082 |  | 1.618 |  | 0.109 |  | 6.844 |  |
| time + stim + ocd\_vs\_hc + stim  ✻  ocd\_vs\_hc |  | 0.053 |  | 0.019 |  | 0.357 |  | 0.026 |  | 4.614 |  |
| time + stim + ocd\_vs\_hc + time  ✻  stim |  | 0.053 |  | 0.012 |  | 0.220 |  | 0.016 |  | 7.178 |  |
| time + stim + ocd\_vs\_hc + time  ✻  ocd\_vs\_hc |  | 0.053 |  | 0.011 |  | 0.194 |  | 0.014 |  | 6.310 |  |
| time + stim + ocd\_vs\_hc + time  ✻  stim + stim  ✻  ocd\_vs\_hc |  | 0.053 |  | 0.002 |  | 0.036 |  | 0.003 |  | 6.817 |  |
| time + stim + ocd\_vs\_hc + time  ✻  ocd\_vs\_hc + stim  ✻  ocd\_vs\_hc |  | 0.053 |  | 0.002 |  | 0.033 |  | 0.002 |  | 6.604 |  |
| time + stim + ocd\_vs\_hc + time  ✻  stim + time  ✻  ocd\_vs\_hc |  | 0.053 |  | 0.001 |  | 0.019 |  | 0.001 |  | 4.972 |  |
| time + stim + ocd\_vs\_hc + time  ✻  stim + time  ✻  ocd\_vs\_hc + stim  ✻  ocd\_vs\_hc |  | 0.053 |  | 1.763e  -4 |  | 0.003 |  | 2.325e  -4 |  | 4.876 |  |
| time + stim + ocd\_vs\_hc + time  ✻  stim + time  ✻  ocd\_vs\_hc + stim  ✻  ocd\_vs\_hc + time  ✻  stim  ✻  ocd\_vs\_hc |  | 0.053 |  | 6.233e  -5 |  | 0.001 |  | 8.222e  -5 |  | 6.232 |  |
| time |  | 0.053 |  | 1.122e  -6 |  | 2.020e  -5 |  | 1.481e  -6 |  | 8.886 |  |
| time + ocd\_vs\_hc |  | 0.053 |  | 1.564e  -7 |  | 2.816e  -6 |  | 2.063e  -7 |  | 4.689 |  |
| time + ocd\_vs\_hc + time  ✻  ocd\_vs\_hc |  | 0.053 |  | 1.362e  -8 |  | 2.451e  -7 |  | 1.796e  -8 |  | 4.544 |  |
| stim |  | 0.053 |  | 3.673e -17 |  | 6.611e -16 |  | 4.844e -17 |  | 4.695 |  |
| stim + ocd\_vs\_hc |  | 0.053 |  | 5.330e -18 |  | 9.594e -17 |  | 7.031e -18 |  | 4.502 |  |
| stim + ocd\_vs\_hc + stim  ✻  ocd\_vs\_hc |  | 0.053 |  | 8.950e -19 |  | 1.611e -17 |  | 1.181e -18 |  | 4.513 |  |
| Null model (incl. subject) |  | 0.053 |  | 4.575e -22 |  | 8.234e -21 |  | 6.035e -22 |  | 4.115 |  |
| ocd\_vs\_hc |  | 0.053 |  | 6.821e -23 |  | 1.228e -21 |  | 8.998e -23 |  | 4.708 |  |
|  | | | | | | | | | | | |
|  |  |  |  |  |  |  |  |  |  |  |  |
| --- | --- | --- | --- | --- | --- | --- | --- | --- | --- | --- | --- |
| *Note.*  All models include subject | | | | | | | | | | | |

## Table S2. SCR conditioning (cs+air)

| Model Comparison | | | | | | | | | | | |
| --- | --- | --- | --- | --- | --- | --- | --- | --- | --- | --- | --- |
| Models | | P(M) | | P(M|data) | | BF M | | BF 10 | | error % | |
| time + stim |  | 0.053 |  | 0.395 |  | 11.735 |  | 1.000 |  |  |  |
| time |  | 0.053 |  | 0.348 |  | 9.592 |  | 0.881 |  | 1.694 |  |
| time + stim + time  ✻  stim |  | 0.053 |  | 0.088 |  | 1.747 |  | 0.224 |  | 1.992 |  |
| time + stim + ocd\_vs\_hc |  | 0.053 |  | 0.069 |  | 1.332 |  | 0.175 |  | 3.139 |  |
| time + ocd\_vs\_hc |  | 0.053 |  | 0.061 |  | 1.161 |  | 0.154 |  | 2.336 |  |
| time + stim + ocd\_vs\_hc + time  ✻  stim |  | 0.053 |  | 0.015 |  | 0.283 |  | 0.039 |  | 2.909 |  |
| time + stim + ocd\_vs\_hc + stim  ✻  ocd\_vs\_hc |  | 0.053 |  | 0.014 |  | 0.264 |  | 0.037 |  | 4.677 |  |
| time + stim + ocd\_vs\_hc + time  ✻  stim + stim  ✻  ocd\_vs\_hc |  | 0.053 |  | 0.003 |  | 0.057 |  | 0.008 |  | 4.327 |  |
| time + stim + ocd\_vs\_hc + time  ✻  ocd\_vs\_hc |  | 0.053 |  | 0.003 |  | 0.056 |  | 0.008 |  | 2.866 |  |
| time + ocd\_vs\_hc + time  ✻  ocd\_vs\_hc |  | 0.053 |  | 0.003 |  | 0.047 |  | 0.007 |  | 1.967 |  |
| time + stim + ocd\_vs\_hc + time  ✻  ocd\_vs\_hc + stim  ✻  ocd\_vs\_hc |  | 0.053 |  | 6.317e  -4 |  | 0.011 |  | 0.002 |  | 4.740 |  |
| time + stim + ocd\_vs\_hc + time  ✻  stim + time  ✻  ocd\_vs\_hc |  | 0.053 |  | 2.036e  -4 |  | 0.004 |  | 5.158e  -4 |  | 45.442 |  |
| time + stim + ocd\_vs\_hc + time  ✻  stim + time  ✻  ocd\_vs\_hc + stim  ✻  ocd\_vs\_hc |  | 0.053 |  | 1.527e  -4 |  | 0.003 |  | 3.870e  -4 |  | 10.552 |  |
| time + stim + ocd\_vs\_hc + time  ✻  stim + time  ✻  ocd\_vs\_hc + stim  ✻  ocd\_vs\_hc + time  ✻  stim  ✻  ocd\_vs\_hc |  | 0.053 |  | 1.923e  -5 |  | 3.462e  -4 |  | 4.874e  -5 |  | 4.109 |  |
| Null model (incl. subject) |  | 0.053 |  | 4.840e -19 |  | 8.712e -18 |  | 1.226e -18 |  | 1.357 |  |
| stim |  | 0.053 |  | 3.722e -19 |  | 6.700e -18 |  | 9.431e -19 |  | 1.701 |  |
| ocd\_vs\_hc |  | 0.053 |  | 8.018e -20 |  | 1.443e -18 |  | 2.032e -19 |  | 2.404 |  |
| stim + ocd\_vs\_hc |  | 0.053 |  | 7.108e -20 |  | 1.279e -18 |  | 1.801e -19 |  | 14.377 |  |
| stim + ocd\_vs\_hc + stim  ✻  ocd\_vs\_hc |  | 0.053 |  | 1.170e -20 |  | 2.105e -19 |  | 2.964e -20 |  | 2.630 |  |
|  | | | | | | | | | | | |
|  |  |  |  |  |  |  |  |  |  |  |  |
| --- | --- | --- | --- | --- | --- | --- | --- | --- | --- | --- | --- |
| *Note.*  All models include subject | | | | | | | | | | | |

## Table S3. SCR extinction (cs+shock)

| Model Comparison | | | | | | | | | | | |
| --- | --- | --- | --- | --- | --- | --- | --- | --- | --- | --- | --- |
| Models | | P(M) | | P(M|data) | | BF M | | BF 10 | | error % | |
| stim |  | 0.053 |  | 0.548 |  | 21.787 |  | 1.000 |  |  |  |
| stim + ocd\_vs\_hc |  | 0.053 |  | 0.196 |  | 4.382 |  | 0.358 |  | 1.968 |  |
| time + stim |  | 0.053 |  | 0.104 |  | 2.093 |  | 0.190 |  | 1.805 |  |
| stim + ocd\_vs\_hc + stim  ✻  ocd\_vs\_hc |  | 0.053 |  | 0.061 |  | 1.173 |  | 0.112 |  | 1.978 |  |
| time + stim + ocd\_vs\_hc |  | 0.053 |  | 0.039 |  | 0.735 |  | 0.072 |  | 5.191 |  |
| time + stim + time  ✻  stim |  | 0.053 |  | 0.020 |  | 0.369 |  | 0.037 |  | 5.713 |  |
| time + stim + ocd\_vs\_hc + stim  ✻  ocd\_vs\_hc |  | 0.053 |  | 0.012 |  | 0.216 |  | 0.022 |  | 2.206 |  |
| time + stim + ocd\_vs\_hc + time  ✻  stim |  | 0.053 |  | 0.007 |  | 0.132 |  | 0.013 |  | 10.178 |  |
| time + stim + ocd\_vs\_hc + time  ✻  ocd\_vs\_hc |  | 0.053 |  | 0.006 |  | 0.112 |  | 0.011 |  | 4.002 |  |
| time + stim + ocd\_vs\_hc + time  ✻  stim + stim  ✻  ocd\_vs\_hc |  | 0.053 |  | 0.002 |  | 0.043 |  | 0.004 |  | 13.955 |  |
| time + stim + ocd\_vs\_hc + time  ✻  ocd\_vs\_hc + stim  ✻  ocd\_vs\_hc |  | 0.053 |  | 0.002 |  | 0.039 |  | 0.004 |  | 4.440 |  |
| time + stim + ocd\_vs\_hc + time  ✻  stim + time  ✻  ocd\_vs\_hc |  | 0.053 |  | 0.001 |  | 0.021 |  | 0.002 |  | 5.683 |  |
| time + stim + ocd\_vs\_hc + time  ✻  stim + time  ✻  ocd\_vs\_hc + stim  ✻  ocd\_vs\_hc |  | 0.053 |  | 3.512e -4 |  | 0.006 |  | 6.413e -4 |  | 3.457 |  |
| Null model (incl. subject) |  | 0.053 |  | 2.527e -4 |  | 0.005 |  | 4.615e -4 |  | 0.995 |  |
| time + stim + ocd\_vs\_hc + time  ✻  stim + time  ✻  ocd\_vs\_hc + stim  ✻  ocd\_vs\_hc + time  ✻  stim  ✻  ocd\_vs\_hc |  | 0.053 |  | 1.646e -4 |  | 0.003 |  | 3.007e -4 |  | 23.325 |  |
| ocd\_vs\_hc |  | 0.053 |  | 8.716e -5 |  | 0.002 |  | 1.592e -4 |  | 1.837 |  |
| time |  | 0.053 |  | 4.862e -5 |  | 8.752e -4 |  | 8.879e -5 |  | 4.086 |  |
| time + ocd\_vs\_hc |  | 0.053 |  | 1.607e -5 |  | 2.893e -4 |  | 2.935e -5 |  | 2.910 |  |
| time + ocd\_vs\_hc + time  ✻  ocd\_vs\_hc |  | 0.053 |  | 2.669e -6 |  | 4.804e -5 |  | 4.874e -6 |  | 3.298 |  |
|  | | | | | | | | | | | |
|  |  |  |  |  |  |  |  |  |  |  |  |
| --- | --- | --- | --- | --- | --- | --- | --- | --- | --- | --- | --- |
| *Note.*  All models include subject | | | | | | | | | | | |

## Table S4. SCR conditioning (cs+shock) frequentist ANOVAs

| Within Subjects Effects | | | | | | | | | | | | | |
| --- | --- | --- | --- | --- | --- | --- | --- | --- | --- | --- | --- | --- | --- |
| Cases | | Sum of Squares | | df | | Mean Square | | F | | p | | η² | |
| time |  | 30.573 | ᵃ | 2 | ᵃ | 15.286 | ᵃ | 32.080 | ᵃ | < .001 | ᵃ | 0.123 |  |
| time ✻ ocd\_vs\_hc |  | 0.618 | ᵃ | 2 | ᵃ | 0.309 | ᵃ | 0.649 | ᵃ | 0.524 | ᵃ | 0.002 |  |
| Residuals |  | 86.724 |  | 182 |  | 0.477 |  |  |  |  |  |  |  |
| stim |  | 10.916 |  | 1 |  | 10.916 |  | 27.450 |  | < .001 |  | 0.044 |  |
| stim ✻ ocd\_vs\_hc |  | 0.215 |  | 1 |  | 0.215 |  | 0.540 |  | 0.464 |  | 8.619e -4 |  |
| Residuals |  | 36.189 |  | 91 |  | 0.398 |  |  |  |  |  |  |  |
| time ✻ stim |  | 0.414 | ᵃ | 2 | ᵃ | 0.207 | ᵃ | 0.668 | ᵃ | 0.514 | ᵃ | 0.002 |  |
| time ✻ stim ✻ ocd\_vs\_hc |  | 1.261 | ᵃ | 2 | ᵃ | 0.630 | ᵃ | 2.031 | ᵃ | 0.134 | ᵃ | 0.005 |  |
| Residuals |  | 56.491 |  | 182 |  | 0.310 |  |  |  |  |  |  |  |
|  | | | | | | | | | | | | | |
|  |  |  |  |  |  |  |  |  |  |  |  |  |  |
| --- | --- | --- | --- | --- | --- | --- | --- | --- | --- | --- | --- | --- | --- |
| *Note.*  Type III Sum of Squares | | | | | | | | | | | | | |
| ᵃ Mauchly's test of sphericity indicates that the assumption of sphericity is violated (p < .05). | | | | | | | | | | | | | |

| Between Subjects Effects | | | | | | | | | | | | | |
| --- | --- | --- | --- | --- | --- | --- | --- | --- | --- | --- | --- | --- | --- |
| Cases | | Sum of Squares | | df | | Mean Square | | F | | p | | η² | |
| ocd\_vs\_hc |  | 0.333 |  | 1 |  | 0.333 |  | 1.188 |  | 0.279 |  | 0.001 |  |
| Residuals |  | 25.493 |  | 91 |  | 0.280 |  |  |  |  |  |  |  |
|  | | | | | | | | | | | | | |
|  |  |  |  |  |  |  |  |  |  |  |  |  |  |
| --- | --- | --- | --- | --- | --- | --- | --- | --- | --- | --- | --- | --- | --- |
| *Note.*  Type III Sum of Squares | | | | | | | | | | | | | |

### Descriptives

| Descriptives | | | | | | | | | | | |
| --- | --- | --- | --- | --- | --- | --- | --- | --- | --- | --- | --- |
| time | | stim | | ocd\_vs\_hc | | Mean | | SD | | N | |
| 1 |  | min |  | 0 |  | 0.262 |  | 0.696 |  | 56 |  |
|  |  |  |  | 1 |  | 0.321 |  | 0.819 |  | 37 |  |
|  |  | shock |  | 0 |  | 0.801 |  | 0.958 |  | 56 |  |
|  |  |  |  | 1 |  | 0.506 |  | 0.570 |  | 37 |  |
| 2 |  | min |  | 0 |  | -0.017 |  | 0.498 |  | 56 |  |
|  |  |  |  | 1 |  | -0.113 |  | 0.482 |  | 37 |  |
|  |  | shock |  | 0 |  | 0.191 |  | 0.602 |  | 56 |  |
|  |  |  |  | 1 |  | 0.138 |  | 0.488 |  | 37 |  |
| 3 |  | min |  | 0 |  | -0.227 |  | 0.430 |  | 56 |  |
|  |  |  |  | 1 |  | -0.219 |  | 0.465 |  | 37 |  |
|  |  | shock |  | 0 |  | 0.004 |  | 0.488 |  | 56 |  |
|  |  |  |  | 1 |  | 0.083 |  | 0.535 |  | 37 |  |
|  | | | | | | | | | | | |

## Table S5. SCR conditioning (cs+air) frequentist ANOVAs

| Within Subjects Effects | | | | | | | | | | | | | |
| --- | --- | --- | --- | --- | --- | --- | --- | --- | --- | --- | --- | --- | --- |
| Cases | | Sum of Squares | | df | | Mean Square | | F | | p | | η² | |
| time |  | 33.731 | ᵃ | 2 | ᵃ | 16.866 | ᵃ | 30.058 | ᵃ | < .001 | ᵃ | 0.148 |  |
| time ✻ ocd\_vs\_hc |  | 0.097 | ᵃ | 2 | ᵃ | 0.048 | ᵃ | 0.086 | ᵃ | 0.917 | ᵃ | 4.237e -4 |  |
| Residuals |  | 100.998 |  | 180 |  | 0.561 |  |  |  |  |  |  |  |
| stim |  | 1.376 |  | 1 |  | 1.376 |  | 5.924 |  | 0.017 |  | 0.006 |  |
| stim ✻ ocd\_vs\_hc |  | 0.298 |  | 1 |  | 0.298 |  | 1.285 |  | 0.260 |  | 0.001 |  |
| Residuals |  | 20.897 |  | 90 |  | 0.232 |  |  |  |  |  |  |  |
| time ✻ stim |  | 1.065 | ᵃ | 2 | ᵃ | 0.532 | ᵃ | 2.201 | ᵃ | 0.114 | ᵃ | 0.005 |  |
| time ✻ stim ✻ ocd\_vs\_hc |  | 0.472 | ᵃ | 2 | ᵃ | 0.236 | ᵃ | 0.975 | ᵃ | 0.379 | ᵃ | 0.002 |  |
| Residuals |  | 43.537 |  | 180 |  | 0.242 |  |  |  |  |  |  |  |
|  | | | | | | | | | | | | | |
|  |  |  |  |  |  |  |  |  |  |  |  |  |  |
| --- | --- | --- | --- | --- | --- | --- | --- | --- | --- | --- | --- | --- | --- |
| *Note.*  Type III Sum of Squares | | | | | | | | | | | | | |
| ᵃ Mauchly's test of sphericity indicates that the assumption of sphericity is violated (p < .05). | | | | | | | | | | | | | |

| Between Subjects Effects | | | | | | | | | | | | | |
| --- | --- | --- | --- | --- | --- | --- | --- | --- | --- | --- | --- | --- | --- |
| Cases | | Sum of Squares | | df | | Mean Square | | F | | p | | η² | |
| ocd\_vs\_hc |  | 0.419 |  | 1 |  | 0.419 |  | 1.466 |  | 0.229 |  | 0.002 |  |
| Residuals |  | 25.745 |  | 90 |  | 0.286 |  |  |  |  |  |  |  |
|  | | | | | | | | | | | | | |
|  |  |  |  |  |  |  |  |  |  |  |  |  |  |
| --- | --- | --- | --- | --- | --- | --- | --- | --- | --- | --- | --- | --- | --- |
| *Note.*  Type III Sum of Squares | | | | | | | | | | | | | |

### Descriptives

| Descriptives | | | | | | | | | | | |
| --- | --- | --- | --- | --- | --- | --- | --- | --- | --- | --- | --- |
| time | | stim | | ocd\_vs\_hc | | Mean | | SD | | N | |
| 1 |  | air |  | 0 |  | 0.565 |  | 0.718 |  | 56 |  |
|  |  |  |  | 1 |  | 0.467 |  | 0.810 |  | 36 |  |
|  |  | min |  | 0 |  | 0.262 |  | 0.696 |  | 56 |  |
|  |  |  |  | 1 |  | 0.316 |  | 0.830 |  | 36 |  |
| 2 |  | air |  | 0 |  | -0.035 |  | 0.537 |  | 56 |  |
|  |  |  |  | 1 |  | -0.057 |  | 0.528 |  | 36 |  |
|  |  | min |  | 0 |  | -0.017 |  | 0.498 |  | 56 |  |
|  |  |  |  | 1 |  | -0.111 |  | 0.489 |  | 36 |  |
| 3 |  | air |  | 0 |  | -0.062 |  | 0.572 |  | 56 |  |
|  |  |  |  | 1 |  | -0.254 |  | 0.400 |  | 36 |  |
|  |  | min |  | 0 |  | -0.227 |  | 0.430 |  | 56 |  |
|  |  |  |  | 1 |  | -0.213 |  | 0.470 |  | 36 |  |
|  | | | | | | | | | | | |

## Table S6. SCR extinction (cs+shock) frequentist ANOVAs

| Within Subjects Effects | | | | | | | | | | | | | |
| --- | --- | --- | --- | --- | --- | --- | --- | --- | --- | --- | --- | --- | --- |
| Cases | | Sum of Squares | | df | | Mean Square | | F | | p | | η² | |
| time |  | 0.145 |  | 1 |  | 0.145 |  | 0.762 |  | 0.385 |  | 0.002 |  |
| time ✻ ocd\_vs\_hc |  | 0.014 |  | 1 |  | 0.014 |  | 0.071 |  | 0.790 |  | 1.793e -4 |  |
| Residuals |  | 17.160 |  | 90 |  | 0.191 |  |  |  |  |  |  |  |
| stim |  | 3.682 |  | 1 |  | 3.682 |  | 19.130 |  | < .001 |  | 0.048 |  |
| stim ✻ ocd\_vs\_hc |  | 0.256 |  | 1 |  | 0.256 |  | 1.331 |  | 0.252 |  | 0.003 |  |
| Residuals |  | 17.323 |  | 90 |  | 0.192 |  |  |  |  |  |  |  |
| time ✻ stim |  | 0.091 |  | 1 |  | 0.091 |  | 0.765 |  | 0.384 |  | 0.001 |  |
| time ✻ stim ✻ ocd\_vs\_hc |  | 0.174 |  | 1 |  | 0.174 |  | 1.465 |  | 0.229 |  | 0.002 |  |
| Residuals |  | 10.669 |  | 90 |  | 0.119 |  |  |  |  |  |  |  |
|  | | | | | | | | | | | | | |
|  |  |  |  |  |  |  |  |  |  |  |  |  |  |
| --- | --- | --- | --- | --- | --- | --- | --- | --- | --- | --- | --- | --- | --- |
| *Note.*  Type III Sum of Squares | | | | | | | | | | | | | |

| Between Subjects Effects | | | | | | | | | | | | | |
| --- | --- | --- | --- | --- | --- | --- | --- | --- | --- | --- | --- | --- | --- |
| Cases | | Sum of Squares | | df | | Mean Square | | F | | p | | η² | |
| ocd\_vs\_hc |  | 0.535 |  | 1 |  | 0.535 |  | 1.859 |  | 0.176 |  | 0.007 |  |
| Residuals |  | 25.916 |  | 90 |  | 0.288 |  |  |  |  |  |  |  |
|  | | | | | | | | | | | | | |
|  |  |  |  |  |  |  |  |  |  |  |  |  |  |
| --- | --- | --- | --- | --- | --- | --- | --- | --- | --- | --- | --- | --- | --- |
| *Note.*  Type III Sum of Squares | | | | | | | | | | | | | |

### Descriptives

| Descriptives | | | | | | | | | | | |
| --- | --- | --- | --- | --- | --- | --- | --- | --- | --- | --- | --- |
| time | | stim | | ocd\_vs\_hc | | Mean | | SD | | N | |
| 1 |  | min |  | 0 |  | -0.329 |  | 0.357 |  | 56 |  |
|  |  |  |  | 1 |  | -0.337 |  | 0.391 |  | 36 |  |
|  |  | shock |  | 0 |  | -0.191 |  | 0.338 |  | 56 |  |
|  |  |  |  | 1 |  | -0.001 |  | 0.497 |  | 36 |  |
| 2 |  | min |  | 0 |  | -0.288 |  | 0.453 |  | 56 |  |
|  |  |  |  | 1 |  | -0.232 |  | 0.576 |  | 36 |  |
|  |  | shock |  | 0 |  | -0.125 |  | 0.506 |  | 56 |  |
|  |  |  |  | 1 |  | -0.050 |  | 0.441 |  | 36 |  |
|  | | | | | | | | | | | |

## Table S7. Pupil dilation conditioning (cs+shock)

| Model Comparison | | | | | | | | | | | |
| --- | --- | --- | --- | --- | --- | --- | --- | --- | --- | --- | --- |
| Models | | P(M) | | P(M|data) | | BF M | | BF 10 | | error % | |
| stim |  | 0.053 |  | 0.753 |  | 55.014 |  | 1.000 |  |  |  |
| stim + ocd\_vs\_hc |  | 0.053 |  | 0.113 |  | 2.296 |  | 0.150 |  | 4.543 |  |
| time + stim |  | 0.053 |  | 0.057 |  | 1.094 |  | 0.076 |  | 22.943 |  |
| time + stim + time  ✻  stim |  | 0.053 |  | 0.040 |  | 0.742 |  | 0.053 |  | 36.787 |  |
| stim + ocd\_vs\_hc + stim  ✻  ocd\_vs\_hc |  | 0.053 |  | 0.020 |  | 0.359 |  | 0.026 |  | 3.203 |  |
| time + stim + ocd\_vs\_hc |  | 0.053 |  | 0.007 |  | 0.118 |  | 0.009 |  | 2.873 |  |
| time + stim + ocd\_vs\_hc + time  ✻  stim |  | 0.053 |  | 0.005 |  | 0.096 |  | 0.007 |  | 29.754 |  |
| time + stim + ocd\_vs\_hc + stim  ✻  ocd\_vs\_hc |  | 0.053 |  | 0.001 |  | 0.026 |  | 0.002 |  | 17.104 |  |
| time + stim + ocd\_vs\_hc + time  ✻  ocd\_vs\_hc |  | 0.053 |  | 0.001 |  | 0.024 |  | 0.002 |  | 4.003 |  |
| time + stim + ocd\_vs\_hc + time  ✻  stim + time  ✻  ocd\_vs\_hc |  | 0.053 |  | 6.980e -4 |  | 0.013 |  | 9.263e -4 |  | 3.026 |  |
| time + stim + ocd\_vs\_hc + time  ✻  stim + stim  ✻  ocd\_vs\_hc |  | 0.053 |  | 6.889e -4 |  | 0.012 |  | 9.144e -4 |  | 3.786 |  |
| Null model (incl. subject) |  | 0.053 |  | 4.835e -4 |  | 0.009 |  | 6.417e -4 |  | 2.331 |  |
| time + stim + ocd\_vs\_hc + time  ✻  ocd\_vs\_hc + stim  ✻  ocd\_vs\_hc |  | 0.053 |  | 2.401e -4 |  | 0.004 |  | 3.187e -4 |  | 4.778 |  |
| time + stim + ocd\_vs\_hc + time  ✻  stim + time  ✻  ocd\_vs\_hc + stim  ✻  ocd\_vs\_hc |  | 0.053 |  | 1.259e -4 |  | 0.002 |  | 1.671e -4 |  | 3.981 |  |
| ocd\_vs\_hc |  | 0.053 |  | 7.166e -5 |  | 0.001 |  | 9.511e -5 |  | 2.615 |  |
| time |  | 0.053 |  | 2.981e -5 |  | 5.365e -4 |  | 3.956e -5 |  | 3.359 |  |
| time + stim + ocd\_vs\_hc + time  ✻  stim + time  ✻  ocd\_vs\_hc + stim  ✻  ocd\_vs\_hc + time  ✻  stim  ✻  ocd\_vs\_hc |  | 0.053 |  | 1.884e -5 |  | 3.392e -4 |  | 2.501e -5 |  | 4.323 |  |
| time + ocd\_vs\_hc |  | 0.053 |  | 4.150e -6 |  | 7.470e -5 |  | 5.507e -6 |  | 2.673 |  |
| time + ocd\_vs\_hc + time  ✻  ocd\_vs\_hc |  | 0.053 |  | 7.200e -7 |  | 1.296e -5 |  | 9.556e -7 |  | 2.959 |  |
|  | | | | | | | | | | | |
|  |  |  |  |  |  |  |  |  |  |  |  |
| --- | --- | --- | --- | --- | --- | --- | --- | --- | --- | --- | --- |
| *Note.*  All models include subject | | | | | | | | | | | |

## Table S8. Pupil dilation conditioning (cs+air)

| Model Comparison | | | | | | | | | | | |
| --- | --- | --- | --- | --- | --- | --- | --- | --- | --- | --- | --- |
| Models | | P(M) | | P(M|data) | | BF M | | BF 10 | | error % | |
| stim |  | 0.053 |  | 0.433 |  | 13.745 |  | 1.000 |  |  |  |
| time + stim |  | 0.053 |  | 0.373 |  | 10.690 |  | 0.861 |  | 1.441 |  |
| time + stim + time  ✻  stim |  | 0.053 |  | 0.063 |  | 1.213 |  | 0.146 |  | 3.595 |  |
| stim + ocd\_vs\_hc |  | 0.053 |  | 0.056 |  | 1.060 |  | 0.128 |  | 2.509 |  |
| time + stim + ocd\_vs\_hc |  | 0.053 |  | 0.046 |  | 0.874 |  | 0.107 |  | 2.078 |  |
| stim + ocd\_vs\_hc + stim  ✻  ocd\_vs\_hc |  | 0.053 |  | 0.008 |  | 0.152 |  | 0.019 |  | 2.224 |  |
| time + stim + ocd\_vs\_hc + time  ✻  stim |  | 0.053 |  | 0.008 |  | 0.146 |  | 0.019 |  | 4.755 |  |
| time + stim + ocd\_vs\_hc + stim  ✻  ocd\_vs\_hc |  | 0.053 |  | 0.008 |  | 0.144 |  | 0.018 |  | 6.996 |  |
| time + stim + ocd\_vs\_hc + time  ✻  ocd\_vs\_hc |  | 0.053 |  | 0.003 |  | 0.049 |  | 0.006 |  | 4.380 |  |
| time + stim + ocd\_vs\_hc + time  ✻  stim + stim  ✻  ocd\_vs\_hc |  | 0.053 |  | 0.001 |  | 0.022 |  | 0.003 |  | 3.133 |  |
| time + stim + ocd\_vs\_hc + time  ✻  stim + time  ✻  ocd\_vs\_hc |  | 0.053 |  | 4.834e  -4 |  | 0.009 |  | 0.001 |  | 9.509 |  |
| time + stim + ocd\_vs\_hc + time  ✻  ocd\_vs\_hc + stim  ✻  ocd\_vs\_hc |  | 0.053 |  | 4.532e  -4 |  | 0.008 |  | 0.001 |  | 11.588 |  |
| time + stim + ocd\_vs\_hc + time  ✻  stim + time  ✻  ocd\_vs\_hc + stim  ✻  ocd\_vs\_hc |  | 0.053 |  | 6.419e  -5 |  | 0.001 |  | 1.482e  -4 |  | 3.339 |  |
| time + stim + ocd\_vs\_hc + time  ✻  stim + time  ✻  ocd\_vs\_hc + stim  ✻  ocd\_vs\_hc + time  ✻  stim  ✻  ocd\_vs\_hc |  | 0.053 |  | 2.581e  -5 |  | 4.645e  -4 |  | 5.960e  -5 |  | 6.640 |  |
| Null model (incl. subject) |  | 0.053 |  | 2.659e -13 |  | 4.786e -12 |  | 6.141e -13 |  | 0.782 |  |
| time |  | 0.053 |  | 1.505e -13 |  | 2.710e -12 |  | 3.477e -13 |  | 1.440 |  |
| ocd\_vs\_hc |  | 0.053 |  | 3.270e -14 |  | 5.886e -13 |  | 7.553e -14 |  | 1.535 |  |
| time + ocd\_vs\_hc |  | 0.053 |  | 1.815e -14 |  | 3.268e -13 |  | 4.193e -14 |  | 1.579 |  |
| time + ocd\_vs\_hc + time  ✻  ocd\_vs\_hc |  | 0.053 |  | 9.993e -16 |  | 1.799e -14 |  | 2.308e -15 |  | 1.974 |  |
|  | | | | | | | | | | | |
|  |  |  |  |  |  |  |  |  |  |  |  |
| --- | --- | --- | --- | --- | --- | --- | --- | --- | --- | --- | --- |
| *Note.*  All models include subject | | | | | | | | | | | |

## Table S9. Pupil dilation extinction (cs+shock)

| Model Comparison | | | | | | | | | | | |
| --- | --- | --- | --- | --- | --- | --- | --- | --- | --- | --- | --- |
| Models | | P(M) | | P(M|data) | | BF M | | BF 10 | | error % | |
| time |  | 0.053 |  | 0.556 |  | 22.561 |  | 1.000 |  |  |  |
| time + ocd\_vs\_hc |  | 0.053 |  | 0.224 |  | 5.189 |  | 0.402 |  | 3.061 |  |
| time + stim |  | 0.053 |  | 0.092 |  | 1.822 |  | 0.165 |  | 2.974 |  |
| time + ocd\_vs\_hc + time  ✻  ocd\_vs\_hc |  | 0.053 |  | 0.049 |  | 0.927 |  | 0.088 |  | 2.412 |  |
| time + stim + ocd\_vs\_hc |  | 0.053 |  | 0.033 |  | 0.616 |  | 0.059 |  | 1.963 |  |
| time + stim + time  ✻  stim |  | 0.053 |  | 0.015 |  | 0.272 |  | 0.027 |  | 2.416 |  |
| time + stim + ocd\_vs\_hc + time  ✻  ocd\_vs\_hc |  | 0.053 |  | 0.009 |  | 0.172 |  | 0.017 |  | 10.058 |  |
| time + stim + ocd\_vs\_hc + stim  ✻  ocd\_vs\_hc |  | 0.053 |  | 0.006 |  | 0.111 |  | 0.011 |  | 3.043 |  |
| time + stim + ocd\_vs\_hc + time  ✻  stim |  | 0.053 |  | 0.006 |  | 0.105 |  | 0.010 |  | 2.809 |  |
| Null model (incl. subject) |  | 0.053 |  | 0.003 |  | 0.062 |  | 0.006 |  | 1.280 |  |
| time + stim + ocd\_vs\_hc + time  ✻  stim + time  ✻  ocd\_vs\_hc |  | 0.053 |  | 0.001 |  | 0.025 |  | 0.003 |  | 3.766 |  |
| time + stim + ocd\_vs\_hc + time  ✻  ocd\_vs\_hc + stim  ✻  ocd\_vs\_hc |  | 0.053 |  | 0.001 |  | 0.025 |  | 0.003 |  | 2.940 |  |
| ocd\_vs\_hc |  | 0.053 |  | 0.001 |  | 0.024 |  | 0.002 |  | 3.382 |  |
| time + stim + ocd\_vs\_hc + time  ✻  stim + stim  ✻  ocd\_vs\_hc |  | 0.053 |  | 9.393e -4 |  | 0.017 |  | 0.002 |  | 3.021 |  |
| stim |  | 0.053 |  | 5.380e -4 |  | 0.010 |  | 9.673e -4 |  | 1.705 |  |
| stim + ocd\_vs\_hc |  | 0.053 |  | 3.102e -4 |  | 0.006 |  | 5.576e -4 |  | 35.870 |  |
| time + stim + ocd\_vs\_hc + time  ✻  stim + time  ✻  ocd\_vs\_hc + stim  ✻  ocd\_vs\_hc |  | 0.053 |  | 2.421e -4 |  | 0.004 |  | 4.352e -4 |  | 5.193 |  |
| time + stim + ocd\_vs\_hc + time  ✻  stim + time  ✻  ocd\_vs\_hc + stim  ✻  ocd\_vs\_hc + time  ✻  stim  ✻  ocd\_vs\_hc |  | 0.053 |  | 9.196e -5 |  | 0.002 |  | 1.653e -4 |  | 4.208 |  |
| stim + ocd\_vs\_hc + stim  ✻  ocd\_vs\_hc |  | 0.053 |  | 3.385e -5 |  | 6.092e -4 |  | 6.085e -5 |  | 2.305 |  |
|  | | | | | | | | | | | |
|  |  |  |  |  |  |  |  |  |  |  |  |
| --- | --- | --- | --- | --- | --- | --- | --- | --- | --- | --- | --- |
| *Note.*  All models include subject | | | | | | | | | | | |

## Table S10. Pupil dilations conditioning (cs+shock) frequentist ANOVAs

| Within Subjects Effects | | | | | | | | | | | | | |
| --- | --- | --- | --- | --- | --- | --- | --- | --- | --- | --- | --- | --- | --- |
| Cases | | Sum of Squares | | df | | Mean Square | | F | | p | | η² | |
| time |  | 0.923 | ᵃ | 2 | ᵃ | 0.461 | ᵃ | 1.312 | ᵃ | 0.272 | ᵃ | 0.006 |  |
| time ✻ ocd\_vs\_hc |  | 0.977 | ᵃ | 2 | ᵃ | 0.489 | ᵃ | 1.389 | ᵃ | 0.252 | ᵃ | 0.006 |  |
| Residuals |  | 57.657 |  | 164 |  | 0.352 |  |  |  |  |  |  |  |
| stim |  | 5.367 |  | 1 |  | 5.367 |  | 13.944 |  | < .001 |  | 0.034 |  |
| stim ✻ ocd\_vs\_hc |  | 0.163 |  | 1 |  | 0.163 |  | 0.424 |  | 0.517 |  | 0.001 |  |
| Residuals |  | 31.560 |  | 82 |  | 0.385 |  |  |  |  |  |  |  |
| time ✻ stim |  | 1.782 |  | 2 |  | 0.891 |  | 3.556 |  | 0.031 |  | 0.011 |  |
| time ✻ stim ✻ ocd\_vs\_hc |  | 0.425 |  | 2 |  | 0.213 |  | 0.848 |  | 0.430 |  | 0.003 |  |
| Residuals |  | 41.099 |  | 164 |  | 0.251 |  |  |  |  |  |  |  |
|  | | | | | | | | | | | | | |
|  |  |  |  |  |  |  |  |  |  |  |  |  |  |
| --- | --- | --- | --- | --- | --- | --- | --- | --- | --- | --- | --- | --- | --- |
| *Note.*  Type III Sum of Squares | | | | | | | | | | | | | |
| ᵃ Mauchly's test of sphericity indicates that the assumption of sphericity is violated (p < .05). | | | | | | | | | | | | | |

| Between Subjects Effects | | | | | | | | | | | | | |
| --- | --- | --- | --- | --- | --- | --- | --- | --- | --- | --- | --- | --- | --- |
| Cases | | Sum of Squares | | df | | Mean Square | | F | | p | | η² | |
| ocd\_vs\_hc |  | 0.194 |  | 1 |  | 0.194 |  | 0.810 |  | 0.371 |  | 0.001 |  |
| Residuals |  | 19.628 |  | 82 |  | 0.239 |  |  |  |  |  |  |  |
|  | | | | | | | | | | | | | |
|  |  |  |  |  |  |  |  |  |  |  |  |  |  |
| --- | --- | --- | --- | --- | --- | --- | --- | --- | --- | --- | --- | --- | --- |
| *Note.*  Type III Sum of Squares | | | | | | | | | | | | | |

### Descriptives

| Descriptives | | | | | | | | | | | |
| --- | --- | --- | --- | --- | --- | --- | --- | --- | --- | --- | --- |
| time | | stim | | ocd\_vs\_hc | | Mean | | SD | | N | |
| 1 |  | min |  | 0 |  | -0.048 |  | 0.485 |  | 50 |  |
|  |  |  |  | 1 |  | 0.126 |  | 0.514 |  | 34 |  |
|  |  | shock |  | 0 |  | 0.093 |  | 0.553 |  | 50 |  |
|  |  |  |  | 1 |  | 0.074 |  | 0.562 |  | 34 |  |
| 2 |  | min |  | 0 |  | -0.056 |  | 0.565 |  | 50 |  |
|  |  |  |  | 1 |  | -0.157 |  | 0.522 |  | 34 |  |
|  |  | shock |  | 0 |  | 0.156 |  | 0.526 |  | 50 |  |
|  |  |  |  | 1 |  | 0.142 |  | 0.476 |  | 34 |  |
| 3 |  | min |  | 0 |  | -0.169 |  | 0.653 |  | 50 |  |
|  |  |  |  | 1 |  | -0.251 |  | 0.547 |  | 34 |  |
|  |  | shock |  | 0 |  | 0.220 |  | 0.503 |  | 50 |  |
|  |  |  |  | 1 |  | 0.022 |  | 0.685 |  | 34 |  |
|  | | | | | | | | | | | |

## Table S11. Pupil dilations conditioning (cs+air) frequentist ANOVAs

| Within Subjects Effects | | | | | | | | | | | | | |
| --- | --- | --- | --- | --- | --- | --- | --- | --- | --- | --- | --- | --- | --- |
| Cases | | Sum of Squares | | df | | Mean Square | | F | | p | | η² | |
| time |  | 2.319 |  | 2 |  | 1.160 |  | 3.257 |  | 0.041 |  | 0.014 |  |
| time ✻ ocd\_vs\_hc |  | 0.165 |  | 2 |  | 0.083 |  | 0.232 |  | 0.793 |  | 9.786e -4 |  |
| Residuals |  | 56.255 |  | 158 |  | 0.356 |  |  |  |  |  |  |  |
| stim |  | 18.635 |  | 1 |  | 18.635 |  | 48.053 |  | < .001 |  | 0.110 |  |
| stim ✻ ocd\_vs\_hc |  | 0.060 |  | 1 |  | 0.060 |  | 0.155 |  | 0.695 |  | 3.547e -4 |  |
| Residuals |  | 30.636 |  | 79 |  | 0.388 |  |  |  |  |  |  |  |
| time ✻ stim |  | 0.944 | ᵃ | 2 | ᵃ | 0.472 | ᵃ | 1.929 | ᵃ | 0.149 | ᵃ | 0.006 |  |
| time ✻ stim ✻ ocd\_vs\_hc |  | 1.058 | ᵃ | 2 | ᵃ | 0.529 | ᵃ | 2.163 | ᵃ | 0.118 | ᵃ | 0.006 |  |
| Residuals |  | 38.657 |  | 158 |  | 0.245 |  |  |  |  |  |  |  |
|  | | | | | | | | | | | | | |
|  |  |  |  |  |  |  |  |  |  |  |  |  |  |
| --- | --- | --- | --- | --- | --- | --- | --- | --- | --- | --- | --- | --- | --- |
| *Note.*  Type III Sum of Squares | | | | | | | | | | | | | |
| ᵃ Mauchly's test of sphericity indicates that the assumption of sphericity is violated (p < .05). | | | | | | | | | | | | | |

| Between Subjects Effects | | | | | | | | | | | | | |
| --- | --- | --- | --- | --- | --- | --- | --- | --- | --- | --- | --- | --- | --- |
| Cases | | Sum of Squares | | df | | Mean Square | | F | | p | | η² | |
| ocd\_vs\_hc |  | 8.481e -4 |  | 1 |  | 8.481e -4 |  | 0.003 |  | 0.954 |  | 5.016e -6 |  |
| Residuals |  | 20.338 |  | 79 |  | 0.257 |  |  |  |  |  |  |  |
|  | | | | | | | | | | | | | |
|  |  |  |  |  |  |  |  |  |  |  |  |  |  |
| --- | --- | --- | --- | --- | --- | --- | --- | --- | --- | --- | --- | --- | --- |
| *Note.*  Type III Sum of Squares | | | | | | | | | | | | | |

### Descriptives

| Descriptives | | | | | | | | | | | |
| --- | --- | --- | --- | --- | --- | --- | --- | --- | --- | --- | --- |
| time | | stim | | ocd\_vs\_hc | | Mean | | SD | | N | |
| 1 |  | air |  | 0 |  | 0.400 |  | 0.575 |  | 48 |  |
|  |  |  |  | 1 |  | 0.291 |  | 0.568 |  | 33 |  |
|  |  | min |  | 0 |  | -0.033 |  | 0.486 |  | 48 |  |
|  |  |  |  | 1 |  | 0.132 |  | 0.520 |  | 33 |  |
| 2 |  | air |  | 0 |  | 0.186 |  | 0.652 |  | 48 |  |
|  |  |  |  | 1 |  | 0.311 |  | 0.523 |  | 33 |  |
|  |  | min |  | 0 |  | -0.104 |  | 0.514 |  | 48 |  |
|  |  |  |  | 1 |  | -0.169 |  | 0.525 |  | 33 |  |
| 3 |  | air |  | 0 |  | 0.338 |  | 0.633 |  | 48 |  |
|  |  |  |  | 1 |  | 0.261 |  | 0.426 |  | 33 |  |
|  |  | min |  | 0 |  | -0.203 |  | 0.592 |  | 48 |  |
|  |  |  |  | 1 |  | -0.227 |  | 0.537 |  | 33 |  |
|  | | | | | | | | | | | |

## Table S12. Pupil dilations extinction (cs+shock) frequentist ANOVAs

| Within Subjects Effects | | | | | | | | | | | | | |
| --- | --- | --- | --- | --- | --- | --- | --- | --- | --- | --- | --- | --- | --- |
| Cases | | Sum of Squares | | df | | Mean Square | | F | | p | | η² | |
| time |  | 4.926 |  | 1 |  | 4.926 |  | 12.184 |  | < .001 |  | 0.042 |  |
| time ✻ ocd\_vs\_hc |  | 0.248 |  | 1 |  | 0.248 |  | 0.613 |  | 0.436 |  | 0.002 |  |
| Residuals |  | 33.558 |  | 83 |  | 0.404 |  |  |  |  |  |  |  |
| stim |  | 0.171 |  | 1 |  | 0.171 |  | 0.525 |  | 0.471 |  | 0.001 |  |
| stim ✻ ocd\_vs\_hc |  | 0.017 |  | 1 |  | 0.017 |  | 0.052 |  | 0.819 |  | 1.465e -4 |  |
| Residuals |  | 27.023 |  | 83 |  | 0.326 |  |  |  |  |  |  |  |
| time ✻ stim |  | 1.852e -4 |  | 1 |  | 1.852e -4 |  | 6.482e -4 |  | 0.980 |  | 1.588e -6 |  |
| time ✻ stim ✻ ocd\_vs\_hc |  | 0.383 |  | 1 |  | 0.383 |  | 1.341 |  | 0.250 |  | 0.003 |  |
| Residuals |  | 23.718 |  | 83 |  | 0.286 |  |  |  |  |  |  |  |
|  | | | | | | | | | | | | | |
|  |  |  |  |  |  |  |  |  |  |  |  |  |  |
| --- | --- | --- | --- | --- | --- | --- | --- | --- | --- | --- | --- | --- | --- |
| *Note.*  Type III Sum of Squares | | | | | | | | | | | | | |

| Between Subjects Effects | | | | | | | | | | | | | |
| --- | --- | --- | --- | --- | --- | --- | --- | --- | --- | --- | --- | --- | --- |
| Cases | | Sum of Squares | | df | | Mean Square | | F | | p | | η² | |
| ocd\_vs\_hc |  | 0.944 |  | 1 |  | 0.944 |  | 3.058 |  | 0.084 |  | 0.008 |  |
| Residuals |  | 25.626 |  | 83 |  | 0.309 |  |  |  |  |  |  |  |
|  | | | | | | | | | | | | | |
|  |  |  |  |  |  |  |  |  |  |  |  |  |  |
| --- | --- | --- | --- | --- | --- | --- | --- | --- | --- | --- | --- | --- | --- |
| *Note.*  Type III Sum of Squares | | | | | | | | | | | | | |

### Descriptives

| Descriptives | | | | | | | | | | | |
| --- | --- | --- | --- | --- | --- | --- | --- | --- | --- | --- | --- |
| time | | stim | | ocd\_vs\_hc | | Mean | | SD | | N | |
| 1 |  | min |  | 0 |  | -0.137 |  | 0.571 |  | 50 |  |
|  |  |  |  | 1 |  | -0.029 |  | 0.627 |  | 35 |  |
|  |  | shock |  | 0 |  | -0.147 |  | 0.552 |  | 50 |  |
|  |  |  |  | 1 |  | 0.069 |  | 0.548 |  | 35 |  |
| 2 |  | min |  | 0 |  | -0.396 |  | 0.551 |  | 50 |  |
|  |  |  |  | 1 |  | -0.262 |  | 0.647 |  | 35 |  |
|  |  | shock |  | 0 |  | -0.267 |  | 0.541 |  | 50 |  |
|  |  |  |  | 1 |  | -0.297 |  | 0.593 |  | 35 |  |
|  | | | | | | | | | | | |

## Table S13. Startle conditioning (cs+shock)

| Model Comparison | | | | | | | | | | | |
| --- | --- | --- | --- | --- | --- | --- | --- | --- | --- | --- | --- |
| Models | | P(M) | | P(M|data) | | BF M | | BF 10 | | error % | |
| time + stim + time  ✻  stim |  | 0.053 |  | 0.798 |  | 71.130 |  | 1.000 |  |  |  |
| time + stim + ocd\_vs\_hc + time  ✻  stim |  | 0.053 |  | 0.154 |  | 3.280 |  | 0.193 |  | 3.479 |  |
| time + stim + ocd\_vs\_hc + time  ✻  stim + time  ✻  ocd\_vs\_hc |  | 0.053 |  | 0.032 |  | 0.595 |  | 0.040 |  | 10.371 |  |
| time + stim |  | 0.053 |  | 0.007 |  | 0.131 |  | 0.009 |  | 3.148 |  |
| time + stim + ocd\_vs\_hc + time  ✻  stim + stim  ✻  ocd\_vs\_hc |  | 0.053 |  | 0.006 |  | 0.105 |  | 0.007 |  | 8.258 |  |
| time + stim + ocd\_vs\_hc |  | 0.053 |  | 0.001 |  | 0.024 |  | 0.002 |  | 2.856 |  |
| time + stim + ocd\_vs\_hc + time  ✻  stim + time  ✻  ocd\_vs\_hc + stim  ✻  ocd\_vs\_hc |  | 0.053 |  | 9.911e  -4 |  | 0.018 |  | 0.001 |  | 4.940 |  |
| time + stim + ocd\_vs\_hc + time  ✻  ocd\_vs\_hc |  | 0.053 |  | 2.458e  -4 |  | 0.004 |  | 3.080e  -4 |  | 5.088 |  |
| time + stim + ocd\_vs\_hc + time  ✻  stim + time  ✻  ocd\_vs\_hc + stim  ✻  ocd\_vs\_hc + time  ✻  stim  ✻  ocd\_vs\_hc |  | 0.053 |  | 1.197e  -4 |  | 0.002 |  | 1.500e  -4 |  | 4.872 |  |
| time + stim + ocd\_vs\_hc + stim  ✻  ocd\_vs\_hc |  | 0.053 |  | 4.758e  -5 |  | 8.565e  -4 |  | 5.962e  -5 |  | 2.938 |  |
| time + stim + ocd\_vs\_hc + time  ✻  ocd\_vs\_hc + stim  ✻  ocd\_vs\_hc |  | 0.053 |  | 9.058e  -6 |  | 1.630e  -4 |  | 1.135e  -5 |  | 3.751 |  |
| time |  | 0.053 |  | 5.042e -11 |  | 9.075e -10 |  | 6.317e -11 |  | 2.984 |  |
| time + ocd\_vs\_hc |  | 0.053 |  | 9.381e -12 |  | 1.689e -10 |  | 1.175e -11 |  | 4.337 |  |
| time + ocd\_vs\_hc + time  ✻  ocd\_vs\_hc |  | 0.053 |  | 1.483e -12 |  | 2.670e -11 |  | 1.859e -12 |  | 3.261 |  |
| stim |  | 0.053 |  | 2.261e -67 |  | 4.070e -66 |  | 2.833e -67 |  | 3.308 |  |
| stim + ocd\_vs\_hc |  | 0.053 |  | 3.450e -68 |  | 6.210e -67 |  | 4.323e -68 |  | 2.964 |  |
| stim + ocd\_vs\_hc + stim  ✻  ocd\_vs\_hc |  | 0.053 |  | 1.197e -69 |  | 2.154e -68 |  | 1.500e -69 |  | 3.052 |  |
| Null model (incl. subject) |  | 0.053 |  | 3.966e -72 |  | 7.138e -71 |  | 4.969e -72 |  | 2.438 |  |
| ocd\_vs\_hc |  | 0.053 |  | 5.984e -73 |  | 1.077e -71 |  | 7.498e -73 |  | 2.582 |  |
|  | | | | | | | | | | | |
|  |  |  |  |  |  |  |  |  |  |  |  |
| --- | --- | --- | --- | --- | --- | --- | --- | --- | --- | --- | --- |
| *Note.*  All models include subject | | | | | | | | | | | |

## Table S14. Startle conditioning (cs+air)

| Model Comparison | | | | | | | | | | | |
| --- | --- | --- | --- | --- | --- | --- | --- | --- | --- | --- | --- |
| Models | | P(M) | | P(M|data) | | BF M | | BF 10 | | error % | |
| time + stim + time  ✻  stim |  | 0.053 |  | 0.625 |  | 30.059 |  | 1.000 |  |  |  |
| time + stim + ocd\_vs\_hc + time  ✻  stim |  | 0.053 |  | 0.297 |  | 7.621 |  | 0.476 |  | 2.745 |  |
| time + stim + ocd\_vs\_hc + time  ✻  stim + time  ✻  ocd\_vs\_hc |  | 0.053 |  | 0.055 |  | 1.039 |  | 0.087 |  | 4.161 |  |
| time + stim + ocd\_vs\_hc + time  ✻  stim + stim  ✻  ocd\_vs\_hc |  | 0.053 |  | 0.016 |  | 0.297 |  | 0.026 |  | 2.670 |  |
| time + stim + ocd\_vs\_hc + time  ✻  stim + time  ✻  ocd\_vs\_hc + stim  ✻  ocd\_vs\_hc |  | 0.053 |  | 0.003 |  | 0.054 |  | 0.005 |  | 3.040 |  |
| time + stim |  | 0.053 |  | 0.002 |  | 0.029 |  | 0.003 |  | 2.044 |  |
| time + stim + ocd\_vs\_hc |  | 0.053 |  | 7.785e  -4 |  | 0.014 |  | 0.001 |  | 3.301 |  |
| time + stim + ocd\_vs\_hc + time  ✻  stim + time  ✻  ocd\_vs\_hc + stim  ✻  ocd\_vs\_hc + time  ✻  stim  ✻  ocd\_vs\_hc |  | 0.053 |  | 7.639e  -4 |  | 0.014 |  | 0.001 |  | 3.047 |  |
| time + stim + ocd\_vs\_hc + time  ✻  ocd\_vs\_hc |  | 0.053 |  | 1.336e  -4 |  | 0.002 |  | 2.136e  -4 |  | 7.517 |  |
| time + stim + ocd\_vs\_hc + stim  ✻  ocd\_vs\_hc |  | 0.053 |  | 5.899e  -5 |  | 0.001 |  | 9.432e  -5 |  | 29.271 |  |
| time + stim + ocd\_vs\_hc + time  ✻  ocd\_vs\_hc + stim  ✻  ocd\_vs\_hc |  | 0.053 |  | 6.906e  -6 |  | 1.243e  -4 |  | 1.104e  -5 |  | 3.383 |  |
| time |  | 0.053 |  | 1.296e -15 |  | 2.332e -14 |  | 2.072e -15 |  | 1.852 |  |
| time + ocd\_vs\_hc |  | 0.053 |  | 5.539e -16 |  | 9.970e -15 |  | 8.856e -16 |  | 5.073 |  |
| time + ocd\_vs\_hc + time  ✻  ocd\_vs\_hc |  | 0.053 |  | 7.752e -17 |  | 1.395e -15 |  | 1.239e -16 |  | 2.980 |  |
| stim |  | 0.053 |  | 7.609e -72 |  | 1.370e -70 |  | 1.216e -71 |  | 2.897 |  |
| stim + ocd\_vs\_hc |  | 0.053 |  | 1.997e -72 |  | 3.595e -71 |  | 3.193e -72 |  | 2.028 |  |
| stim + ocd\_vs\_hc + stim  ✻  ocd\_vs\_hc |  | 0.053 |  | 9.164e -74 |  | 1.650e -72 |  | 1.465e -73 |  | 2.756 |  |
| Null model (incl. subject) |  | 0.053 |  | 3.756e -79 |  | 6.760e -78 |  | 6.004e -79 |  | 1.739 |  |
| ocd\_vs\_hc |  | 0.053 |  | 1.029e -79 |  | 1.851e -78 |  | 1.644e -79 |  | 4.180 |  |
|  | | | | | | | | | | | |
|  |  |  |  |  |  |  |  |  |  |  |  |
| --- | --- | --- | --- | --- | --- | --- | --- | --- | --- | --- | --- |
| *Note.*  All models include subject | | | | | | | | | | | |

## Table S15. Startle extinction (cs+shock)

| Model Comparison | | | | | | | | | | | |
| --- | --- | --- | --- | --- | --- | --- | --- | --- | --- | --- | --- |
| Models | | P(M) | | P(M|data) | | BF M | | BF 10 | | error % | |
| time + stim + ocd\_vs\_hc |  | 0.053 |  | 0.636 |  | 31.418 |  | 1.000 |  |  |  |
| time + stim |  | 0.053 |  | 0.124 |  | 2.542 |  | 0.195 |  | 19.475 |  |
| time + stim + ocd\_vs\_hc + stim  ✻  ocd\_vs\_hc |  | 0.053 |  | 0.100 |  | 2.001 |  | 0.157 |  | 19.272 |  |
| time + stim + ocd\_vs\_hc + time  ✻  ocd\_vs\_hc |  | 0.053 |  | 0.069 |  | 1.342 |  | 0.109 |  | 19.327 |  |
| time + stim + ocd\_vs\_hc + time  ✻  stim |  | 0.053 |  | 0.034 |  | 0.628 |  | 0.053 |  | 19.353 |  |
| time + stim + ocd\_vs\_hc + time  ✻  ocd\_vs\_hc + stim  ✻  ocd\_vs\_hc |  | 0.053 |  | 0.016 |  | 0.295 |  | 0.025 |  | 21.826 |  |
| time + stim + time  ✻  stim |  | 0.053 |  | 0.008 |  | 0.148 |  | 0.013 |  | 19.278 |  |
| time + stim + ocd\_vs\_hc + time  ✻  stim + stim  ✻  ocd\_vs\_hc |  | 0.053 |  | 0.007 |  | 0.124 |  | 0.011 |  | 19.337 |  |
| time + stim + ocd\_vs\_hc + time  ✻  stim + time  ✻  ocd\_vs\_hc |  | 0.053 |  | 0.005 |  | 0.092 |  | 0.008 |  | 19.843 |  |
| time + stim + ocd\_vs\_hc + time  ✻  stim + time  ✻  ocd\_vs\_hc + stim  ✻  ocd\_vs\_hc |  | 0.053 |  | 9.444e  -4 |  | 0.017 |  | 0.001 |  | 19.515 |  |
| time + stim + ocd\_vs\_hc + time  ✻  stim + time  ✻  ocd\_vs\_hc + stim  ✻  ocd\_vs\_hc + time  ✻  stim  ✻  ocd\_vs\_hc |  | 0.053 |  | 1.347e  -4 |  | 0.002 |  | 2.118e  -4 |  | 20.874 |  |
| stim + ocd\_vs\_hc |  | 0.053 |  | 6.244e  -5 |  | 0.001 |  | 9.821e  -5 |  | 19.217 |  |
| stim |  | 0.053 |  | 1.720e  -5 |  | 3.096e  -4 |  | 2.705e  -5 |  | 19.191 |  |
| stim + ocd\_vs\_hc + stim  ✻  ocd\_vs\_hc |  | 0.053 |  | 1.221e  -5 |  | 2.198e  -4 |  | 1.921e  -5 |  | 19.305 |  |
| time + ocd\_vs\_hc |  | 0.053 |  | 1.527e  -7 |  | 2.749e  -6 |  | 2.402e  -7 |  | 19.231 |  |
| time |  | 0.053 |  | 4.613e  -8 |  | 8.304e  -7 |  | 7.256e  -8 |  | 19.296 |  |
| time + ocd\_vs\_hc + time  ✻  ocd\_vs\_hc |  | 0.053 |  | 2.148e  -8 |  | 3.866e  -7 |  | 3.378e  -8 |  | 19.681 |  |
| ocd\_vs\_hc |  | 0.053 |  | 4.377e -11 |  | 7.878e -10 |  | 6.885e -11 |  | 19.195 |  |
| Null model (incl. subject) |  | 0.053 |  | 1.446e -11 |  | 2.602e -10 |  | 2.274e -11 |  | 19.179 |  |
|  | | | | | | | | | | | |
|  |  |  |  |  |  |  |  |  |  |  |  |
| --- | --- | --- | --- | --- | --- | --- | --- | --- | --- | --- | --- |
| *Note.*  All models include subject | | | | | | | | | | | |

## Table S16. Startle conditioning (cs+shock) frequentist ANOVAs

| Within Subjects Effects | | | | | | | | | | | | | |
| --- | --- | --- | --- | --- | --- | --- | --- | --- | --- | --- | --- | --- | --- |
| Cases | | Sum of Squares | | df | | Mean Square | | F | | p | | η² | |
| time |  | 112.568 | ᵃ | 2 | ᵃ | 56.284 | ᵃ | 149.403 | ᵃ | < .001 | ᵃ | 0.316 |  |
| time ✻ ocd\_vs\_hc |  | 1.146 | ᵃ | 2 | ᵃ | 0.573 | ᵃ | 1.521 | ᵃ | 0.222 | ᵃ | 0.003 |  |
| Residuals |  | 59.523 |  | 158 |  | 0.377 |  |  |  |  |  |  |  |
| stim |  | 14.829 |  | 2 |  | 7.414 |  | 21.884 |  | < .001 |  | 0.042 |  |
| stim ✻ ocd\_vs\_hc |  | 0.116 |  | 2 |  | 0.058 |  | 0.171 |  | 0.843 |  | 3.264e -4 |  |
| Residuals |  | 53.530 |  | 158 |  | 0.339 |  |  |  |  |  |  |  |
| time ✻ stim |  | 6.483 | ᵃ | 4 | ᵃ | 1.621 | ᵃ | 5.820 | ᵃ | < .001 | ᵃ | 0.018 |  |
| time ✻ stim ✻ ocd\_vs\_hc |  | 1.511 | ᵃ | 4 | ᵃ | 0.378 | ᵃ | 1.356 | ᵃ | 0.249 | ᵃ | 0.004 |  |
| Residuals |  | 88.012 |  | 316 |  | 0.279 |  |  |  |  |  |  |  |
|  | | | | | | | | | | | | | |
|  |  |  |  |  |  |  |  |  |  |  |  |  |  |
| --- | --- | --- | --- | --- | --- | --- | --- | --- | --- | --- | --- | --- | --- |
| *Note.*  Type III Sum of Squares | | | | | | | | | | | | | |
| ᵃ Mauchly's test of sphericity indicates that the assumption of sphericity is violated (p < .05). | | | | | | | | | | | | | |

| Between Subjects Effects | | | | | | | | | | | | | |
| --- | --- | --- | --- | --- | --- | --- | --- | --- | --- | --- | --- | --- | --- |
| Cases | | Sum of Squares | | df | | Mean Square | | F | | p | | η² | |
| ocd\_vs\_hc |  | 0.629 |  | 1 |  | 0.629 |  | 2.838 |  | 0.096 |  | 0.002 |  |
| Residuals |  | 17.516 |  | 79 |  | 0.222 |  |  |  |  |  |  |  |
|  | | | | | | | | | | | | | |
|  |  |  |  |  |  |  |  |  |  |  |  |  |  |
| --- | --- | --- | --- | --- | --- | --- | --- | --- | --- | --- | --- | --- | --- |
| *Note.*  Type III Sum of Squares | | | | | | | | | | | | | |

### Descriptives

| Descriptives | | | | | | | | | | | |
| --- | --- | --- | --- | --- | --- | --- | --- | --- | --- | --- | --- |
| time | | stim | | ocd\_vs\_hc | | Mean | | SD | | N | |
| 1 |  | iti |  | 0 |  | 0.806 |  | 0.594 |  | 47 |  |
|  |  |  |  | 1 |  | 0.534 |  | 0.558 |  | 34 |  |
|  |  | min |  | 0 |  | 0.645 |  | 0.695 |  | 47 |  |
|  |  |  |  | 1 |  | 0.468 |  | 0.659 |  | 34 |  |
|  |  | shock |  | 0 |  | 0.771 |  | 0.684 |  | 47 |  |
|  |  |  |  | 1 |  | 0.782 |  | 0.802 |  | 34 |  |
| 2 |  | iti |  | 0 |  | -0.169 |  | 0.486 |  | 47 |  |
|  |  |  |  | 1 |  | -0.217 |  | 0.553 |  | 34 |  |
|  |  | min |  | 0 |  | 0.037 |  | 0.449 |  | 47 |  |
|  |  |  |  | 1 |  | 0.082 |  | 0.598 |  | 34 |  |
|  |  | shock |  | 0 |  | 0.294 |  | 0.508 |  | 47 |  |
|  |  |  |  | 1 |  | 0.058 |  | 0.652 |  | 34 |  |
| 3 |  | iti |  | 0 |  | -0.629 |  | 0.387 |  | 47 |  |
|  |  |  |  | 1 |  | -0.575 |  | 0.335 |  | 34 |  |
|  |  | min |  | 0 |  | -0.267 |  | 0.374 |  | 47 |  |
|  |  |  |  | 1 |  | -0.213 |  | 0.543 |  | 34 |  |
|  |  | shock |  | 0 |  | -0.033 |  | 0.447 |  | 47 |  |
|  |  |  |  | 1 |  | 3.318e -4 |  | 0.521 |  | 34 |  |
|  | | | | | | | | | | | |

## Table S17. Startle conditioning (cs+air) frequentist ANOVAs

| Within Subjects Effects | | | | | | | | | | | | | |
| --- | --- | --- | --- | --- | --- | --- | --- | --- | --- | --- | --- | --- | --- |
| Cases | | Sum of Squares | | df | | Mean Square | | F | | p | | η² | |
| time |  | 116.321 | ᵃ | 2 | ᵃ | 58.160 | ᵃ | 169.540 | ᵃ | < .001 | ᵃ | 0.315 |  |
| time ✻ ocd\_vs\_hc |  | 1.098 | ᵃ | 2 | ᵃ | 0.549 | ᵃ | 1.600 | ᵃ | 0.205 | ᵃ | 0.003 |  |
| Residuals |  | 56.260 |  | 164 |  | 0.343 |  |  |  |  |  |  |  |
| stim |  | 19.653 |  | 2 |  | 9.827 |  | 30.976 |  | < .001 |  | 0.053 |  |
| stim ✻ ocd\_vs\_hc |  | 0.386 |  | 2 |  | 0.193 |  | 0.608 |  | 0.545 |  | 0.001 |  |
| Residuals |  | 52.027 |  | 164 |  | 0.317 |  |  |  |  |  |  |  |
| time ✻ stim |  | 6.775 |  | 4 |  | 1.694 |  | 5.821 |  | < .001 |  | 0.018 |  |
| time ✻ stim ✻ ocd\_vs\_hc |  | 2.121 |  | 4 |  | 0.530 |  | 1.822 |  | 0.124 |  | 0.006 |  |
| Residuals |  | 95.434 |  | 328 |  | 0.291 |  |  |  |  |  |  |  |
|  | | | | | | | | | | | | | |
|  |  |  |  |  |  |  |  |  |  |  |  |  |  |
| --- | --- | --- | --- | --- | --- | --- | --- | --- | --- | --- | --- | --- | --- |
| *Note.*  Type III Sum of Squares | | | | | | | | | | | | | |
| ᵃ Mauchly's test of sphericity indicates that the assumption of sphericity is violated (p < .05). | | | | | | | | | | | | | |

| Between Subjects Effects | | | | | | | | | | | | | |
| --- | --- | --- | --- | --- | --- | --- | --- | --- | --- | --- | --- | --- | --- |
| Cases | | Sum of Squares | | df | | Mean Square | | F | | p | | η² | |
| ocd\_vs\_hc |  | 1.471 |  | 1 |  | 1.471 |  | 6.864 |  | 0.010 |  | 0.004 |  |
| Residuals |  | 17.575 |  | 82 |  | 0.214 |  |  |  |  |  |  |  |
|  | | | | | | | | | | | | | |
|  |  |  |  |  |  |  |  |  |  |  |  |  |  |
| --- | --- | --- | --- | --- | --- | --- | --- | --- | --- | --- | --- | --- | --- |
| *Note.*  Type III Sum of Squares | | | | | | | | | | | | | |

### Descriptives

| Descriptives | | | | | | | | | | | |
| --- | --- | --- | --- | --- | --- | --- | --- | --- | --- | --- | --- |
| time | | stim | | ocd\_vs\_hc | | Mean | | SD | | N | |
| 1 |  | air |  | 0 |  | 0.826 |  | 0.735 |  | 50 |  |
|  |  |  |  | 1 |  | 0.813 |  | 0.525 |  | 34 |  |
|  |  | iti |  | 0 |  | 0.824 |  | 0.585 |  | 50 |  |
|  |  |  |  | 1 |  | 0.534 |  | 0.558 |  | 34 |  |
|  |  | min |  | 0 |  | 0.647 |  | 0.682 |  | 50 |  |
|  |  |  |  | 1 |  | 0.468 |  | 0.659 |  | 34 |  |
| 2 |  | air |  | 0 |  | 0.423 |  | 0.507 |  | 50 |  |
|  |  |  |  | 1 |  | 0.082 |  | 0.481 |  | 34 |  |
|  |  | iti |  | 0 |  | -0.147 |  | 0.501 |  | 50 |  |
|  |  |  |  | 1 |  | -0.217 |  | 0.553 |  | 34 |  |
|  |  | min |  | 0 |  | 0.052 |  | 0.503 |  | 50 |  |
|  |  |  |  | 1 |  | 0.082 |  | 0.598 |  | 34 |  |
| 3 |  | air |  | 0 |  | 0.054 |  | 0.605 |  | 50 |  |
|  |  |  |  | 1 |  | -0.015 |  | 0.516 |  | 34 |  |
|  |  | iti |  | 0 |  | -0.636 |  | 0.390 |  | 50 |  |
|  |  |  |  | 1 |  | -0.575 |  | 0.335 |  | 34 |  |
|  |  | min |  | 0 |  | -0.274 |  | 0.379 |  | 50 |  |
|  |  |  |  | 1 |  | -0.213 |  | 0.543 |  | 34 |  |
|  | | | | | | | | | | | |

## Table S18. Startle extinction (cs+shock) frequentist ANOVAs

| Within Subjects Effects | | | | | | | | | | | | | |
| --- | --- | --- | --- | --- | --- | --- | --- | --- | --- | --- | --- | --- | --- |
| Cases | | Sum of Squares | | df | | Mean Square | | F | | p | | η² | |
| time |  | 4.583 |  | 1 |  | 4.583 |  | 20.642 |  | < .001 |  | 0.037 |  |
| time ✻ ocd\_vs\_hc |  | 0.002 |  | 1 |  | 0.002 |  | 0.008 |  | 0.931 |  | 1.354e -5 |  |
| Residuals |  | 18.429 |  | 83 |  | 0.222 |  |  |  |  |  |  |  |
| stim |  | 8.751 |  | 2 |  | 4.375 |  | 16.812 |  | < .001 |  | 0.071 |  |
| stim ✻ ocd\_vs\_hc |  | 0.728 |  | 2 |  | 0.364 |  | 1.399 |  | 0.250 |  | 0.006 |  |
| Residuals |  | 43.201 |  | 166 |  | 0.260 |  |  |  |  |  |  |  |
| time ✻ stim |  | 0.313 | ᵃ | 2 | ᵃ | 0.156 | ᵃ | 0.944 | ᵃ | 0.391 | ᵃ | 0.003 |  |
| time ✻ stim ✻ ocd\_vs\_hc |  | 0.273 | ᵃ | 2 | ᵃ | 0.137 | ᵃ | 0.825 | ᵃ | 0.440 | ᵃ | 0.002 |  |
| Residuals |  | 27.503 |  | 166 |  | 0.166 |  |  |  |  |  |  |  |
|  | | | | | | | | | | | | | |
|  |  |  |  |  |  |  |  |  |  |  |  |  |  |
| --- | --- | --- | --- | --- | --- | --- | --- | --- | --- | --- | --- | --- | --- |
| *Note.*  Type III Sum of Squares | | | | | | | | | | | | | |
| ᵃ Mauchly's test of sphericity indicates that the assumption of sphericity is violated (p < .05). | | | | | | | | | | | | | |

| Between Subjects Effects | | | | | | | | | | | | | |
| --- | --- | --- | --- | --- | --- | --- | --- | --- | --- | --- | --- | --- | --- |
| Cases | | Sum of Squares | | df | | Mean Square | | F | | p | | η² | |
| ocd\_vs\_hc |  | 2.365 |  | 1 |  | 2.365 |  | 11.120 |  | 0.001 |  | 0.019 |  |
| Residuals |  | 17.651 |  | 83 |  | 0.213 |  |  |  |  |  |  |  |
|  | | | | | | | | | | | | | |
|  |  |  |  |  |  |  |  |  |  |  |  |  |  |
| --- | --- | --- | --- | --- | --- | --- | --- | --- | --- | --- | --- | --- | --- |
| *Note.*  Type III Sum of Squares | | | | | | | | | | | | | |

### Descriptives

| Descriptives | | | | | | | | | | | |
| --- | --- | --- | --- | --- | --- | --- | --- | --- | --- | --- | --- |
| time | | stim | | ocd\_vs\_hc | | Mean | | SD | | N | |
| 1 |  | iti |  | 0 |  | -0.564 |  | 0.487 |  | 50 |  |
|  |  |  |  | 1 |  | -0.521 |  | 0.511 |  | 35 |  |
|  |  | min |  | 0 |  | -0.337 |  | 0.535 |  | 50 |  |
|  |  |  |  | 1 |  | -0.145 |  | 0.434 |  | 35 |  |
|  |  | shock |  | 0 |  | -0.368 |  | 0.421 |  | 50 |  |
|  |  |  |  | 1 |  | -0.200 |  | 0.478 |  | 35 |  |
| 2 |  | iti |  | 0 |  | -0.759 |  | 0.398 |  | 50 |  |
|  |  |  |  | 1 |  | -0.716 |  | 0.484 |  | 35 |  |
|  |  | min |  | 0 |  | -0.538 |  | 0.389 |  | 50 |  |
|  |  |  |  | 1 |  | -0.450 |  | 0.480 |  | 35 |  |
|  |  | shock |  | 0 |  | -0.561 |  | 0.450 |  | 50 |  |
|  |  |  |  | 1 |  | -0.266 |  | 0.501 |  | 35 |  |
|  | | | | | | | | | | | |

## Table S19. Shock expectancy ratings conditioning

| Model Comparison | | | | | | | | | | | |
| --- | --- | --- | --- | --- | --- | --- | --- | --- | --- | --- | --- |
| Models | | P(M) | | P(M|data) | | BF M | | BF 10 | | error % | |
| time + stim + time  ✻  stim |  | 0.053 |  | 0.648 |  | 33.142 |  | 1.000 |  |  |  |
| time + stim + ocd\_vs\_hc + time  ✻  stim + stim  ✻  ocd\_vs\_hc |  | 0.053 |  | 0.232 |  | 5.431 |  | 0.358 |  | 4.671 |  |
| time + stim + ocd\_vs\_hc + time  ✻  stim |  | 0.053 |  | 0.087 |  | 1.723 |  | 0.135 |  | 3.911 |  |
| time + stim + ocd\_vs\_hc + time  ✻  stim + time  ✻  ocd\_vs\_hc + stim  ✻  ocd\_vs\_hc |  | 0.053 |  | 0.022 |  | 0.410 |  | 0.034 |  | 3.758 |  |
| time + stim + ocd\_vs\_hc + time  ✻  stim + time  ✻  ocd\_vs\_hc |  | 0.053 |  | 0.009 |  | 0.164 |  | 0.014 |  | 4.484 |  |
| time + stim + ocd\_vs\_hc + time  ✻  stim + time  ✻  ocd\_vs\_hc + stim  ✻  ocd\_vs\_hc + time  ✻  stim  ✻  ocd\_vs\_hc |  | 0.053 |  | 0.001 |  | 0.026 |  | 0.002 |  | 6.329 |  |
| stim |  | 0.053 |  | 3.260e  -63 |  | 5.869e  -62 |  | 5.031e  -63 |  | 3.391 |  |
| stim + ocd\_vs\_hc |  | 0.053 |  | 4.235e  -64 |  | 7.623e  -63 |  | 6.535e  -64 |  | 6.010 |  |
| stim + ocd\_vs\_hc + stim  ✻  ocd\_vs\_hc |  | 0.053 |  | 3.564e  -64 |  | 6.415e  -63 |  | 5.499e  -64 |  | 4.492 |  |
| time + stim |  | 0.053 |  | 2.049e  -65 |  | 3.688e  -64 |  | 3.162e  -65 |  | 3.510 |  |
| time + stim + ocd\_vs\_hc |  | 0.053 |  | 4.140e  -66 |  | 7.451e  -65 |  | 6.388e  -66 |  | 35.303 |  |
| time + stim + ocd\_vs\_hc + stim  ✻  ocd\_vs\_hc |  | 0.053 |  | 2.357e  -66 |  | 4.243e  -65 |  | 3.638e  -66 |  | 6.348 |  |
| time + stim + ocd\_vs\_hc + time  ✻  ocd\_vs\_hc |  | 0.053 |  | 1.278e  -67 |  | 2.301e  -66 |  | 1.972e  -67 |  | 4.819 |  |
| time + stim + ocd\_vs\_hc + time  ✻  ocd\_vs\_hc + stim  ✻  ocd\_vs\_hc |  | 0.053 |  | 1.030e  -67 |  | 1.854e  -66 |  | 1.590e  -67 |  | 4.221 |  |
| Null model (incl. subject) |  | 0.053 |  | 1.509e -167 |  | 2.716e -166 |  | 2.328e -167 |  | 3.295 |  |
| ocd\_vs\_hc |  | 0.053 |  | 1.675e -168 |  | 3.014e -167 |  | 2.584e -168 |  | 3.659 |  |
| time |  | 0.053 |  | 8.030e -170 |  | 1.445e -168 |  | 1.239e -169 |  | 3.371 |  |
| time + ocd\_vs\_hc |  | 0.053 |  | 8.803e -171 |  | 1.585e -169 |  | 1.358e -170 |  | 3.573 |  |
| time + ocd\_vs\_hc + time  ✻  ocd\_vs\_hc |  | 0.053 |  | 2.341e -172 |  | 4.214e -171 |  | 3.613e -172 |  | 6.285 |  |
|  | | | | | | | | | | | |
|  |  |  |  |  |  |  |  |  |  |  |  |
| --- | --- | --- | --- | --- | --- | --- | --- | --- | --- | --- | --- |
| *Note.*  All models include subject | | | | | | | | | | | |

## Table S20. Airblast expectancy ratings conditioning

| Model Comparison | | | | | | | | | | | |
| --- | --- | --- | --- | --- | --- | --- | --- | --- | --- | --- | --- |
| Models | | P(M) | | P(M|data) | | BF M | | BF 10 | | error % | |
| time + stim + time  ✻  stim |  | 0.053 |  | 0.790 |  | 67.595 |  | 1.000 |  |  |  |
| time + stim + ocd\_vs\_hc + time  ✻  stim |  | 0.053 |  | 0.166 |  | 3.592 |  | 0.211 |  | 2.899 |  |
| time + stim + ocd\_vs\_hc + time  ✻  stim + stim  ✻  ocd\_vs\_hc |  | 0.053 |  | 0.024 |  | 0.443 |  | 0.030 |  | 10.805 |  |
| time + stim + ocd\_vs\_hc + time  ✻  stim + time  ✻  ocd\_vs\_hc |  | 0.053 |  | 0.015 |  | 0.274 |  | 0.019 |  | 3.924 |  |
| time + stim + ocd\_vs\_hc + time  ✻  stim + time  ✻  ocd\_vs\_hc + stim  ✻  ocd\_vs\_hc + time  ✻  stim  ✻  ocd\_vs\_hc |  | 0.053 |  | 0.003 |  | 0.055 |  | 0.004 |  | 22.703 |  |
| time + stim + ocd\_vs\_hc + time  ✻  stim + time  ✻  ocd\_vs\_hc + stim  ✻  ocd\_vs\_hc |  | 0.053 |  | 0.002 |  | 0.034 |  | 0.002 |  | 3.321 |  |
| stim |  | 0.053 |  | 2.221e  -68 |  | 3.998e  -67 |  | 2.813e  -68 |  | 2.165 |  |
| stim + ocd\_vs\_hc |  | 0.053 |  | 3.743e  -69 |  | 6.737e  -68 |  | 4.740e  -69 |  | 4.165 |  |
| stim + ocd\_vs\_hc + stim  ✻  ocd\_vs\_hc |  | 0.053 |  | 4.360e  -70 |  | 7.848e  -69 |  | 5.521e  -70 |  | 3.076 |  |
| time + stim |  | 0.053 |  | 2.353e  -70 |  | 4.235e  -69 |  | 2.980e  -70 |  | 2.327 |  |
| time + stim + ocd\_vs\_hc |  | 0.053 |  | 3.778e  -71 |  | 6.800e  -70 |  | 4.784e  -71 |  | 2.459 |  |
| time + stim + ocd\_vs\_hc + stim  ✻  ocd\_vs\_hc |  | 0.053 |  | 5.152e  -72 |  | 9.273e  -71 |  | 6.524e  -72 |  | 6.355 |  |
| time + stim + ocd\_vs\_hc + time  ✻  ocd\_vs\_hc |  | 0.053 |  | 1.562e  -72 |  | 2.811e  -71 |  | 1.978e  -72 |  | 2.546 |  |
| time + stim + ocd\_vs\_hc + time  ✻  ocd\_vs\_hc + stim  ✻  ocd\_vs\_hc |  | 0.053 |  | 1.943e  -73 |  | 3.497e  -72 |  | 2.460e  -73 |  | 3.121 |  |
| Null model (incl. subject) |  | 0.053 |  | 3.362e -172 |  | 6.051e -171 |  | 4.257e -172 |  | 1.861 |  |
| ocd\_vs\_hc |  | 0.053 |  | 4.340e -173 |  | 7.812e -172 |  | 5.496e -173 |  | 2.291 |  |
| time |  | 0.053 |  | 2.357e -174 |  | 4.243e -173 |  | 2.985e -174 |  | 1.964 |  |
| time + ocd\_vs\_hc |  | 0.053 |  | 3.068e -175 |  | 5.523e -174 |  | 3.885e -175 |  | 2.900 |  |
| time + ocd\_vs\_hc + time  ✻  ocd\_vs\_hc |  | 0.053 |  | 7.389e -177 |  | 1.330e -175 |  | 9.357e -177 |  | 6.953 |  |
|  | | | | | | | | | | | |
|  |  |  |  |  |  |  |  |  |  |  |  |
| --- | --- | --- | --- | --- | --- | --- | --- | --- | --- | --- | --- |
| *Note.*  All models include subject | | | | | | | | | | | |

## Table S21. Shock expectancy ratings extinction

| Model Comparison | | | | | | | | | | | |
| --- | --- | --- | --- | --- | --- | --- | --- | --- | --- | --- | --- |
| Models | | P(M) | | P(M|data) | | BF M | | BF 10 | | error % | |
| stim |  | 0.053 |  | 0.460 |  | 15.345 |  | 1.000 |  |  |  |
| time + stim |  | 0.053 |  | 0.261 |  | 6.366 |  | 0.568 |  | 1.975 |  |
| stim + ocd\_vs\_hc |  | 0.053 |  | 0.111 |  | 2.256 |  | 0.242 |  | 3.255 |  |
| time + stim + ocd\_vs\_hc |  | 0.053 |  | 0.063 |  | 1.215 |  | 0.137 |  | 2.851 |  |
| time + stim + time  ✻  stim |  | 0.053 |  | 0.047 |  | 0.896 |  | 0.103 |  | 3.447 |  |
| stim + ocd\_vs\_hc + stim  ✻  ocd\_vs\_hc |  | 0.053 |  | 0.017 |  | 0.318 |  | 0.038 |  | 3.793 |  |
| time + stim + ocd\_vs\_hc + time  ✻  ocd\_vs\_hc |  | 0.053 |  | 0.011 |  | 0.206 |  | 0.025 |  | 3.610 |  |
| time + stim + ocd\_vs\_hc + time  ✻  stim |  | 0.053 |  | 0.011 |  | 0.202 |  | 0.024 |  | 3.806 |  |
| time + stim + ocd\_vs\_hc + stim  ✻  ocd\_vs\_hc |  | 0.053 |  | 0.010 |  | 0.184 |  | 0.022 |  | 3.388 |  |
| time + stim + ocd\_vs\_hc + time  ✻  stim + time  ✻  ocd\_vs\_hc |  | 0.053 |  | 0.002 |  | 0.040 |  | 0.005 |  | 5.555 |  |
| time + stim + ocd\_vs\_hc + time  ✻  ocd\_vs\_hc + stim  ✻  ocd\_vs\_hc |  | 0.053 |  | 0.002 |  | 0.036 |  | 0.004 |  | 5.947 |  |
| time + stim + ocd\_vs\_hc + time  ✻  stim + stim  ✻  ocd\_vs\_hc |  | 0.053 |  | 0.002 |  | 0.035 |  | 0.004 |  | 7.705 |  |
| time + stim + ocd\_vs\_hc + time  ✻  stim + time  ✻  ocd\_vs\_hc + stim  ✻  ocd\_vs\_hc |  | 0.053 |  | 3.299e  -4 |  | 0.006 |  | 7.168e  -4 |  | 2.810 |  |
| time + stim + ocd\_vs\_hc + time  ✻  stim + time  ✻  ocd\_vs\_hc + stim  ✻  ocd\_vs\_hc + time  ✻  stim  ✻  ocd\_vs\_hc |  | 0.053 |  | 7.551e  -5 |  | 0.001 |  | 1.641e  -4 |  | 4.274 |  |
| Null model (incl. subject) |  | 0.053 |  | 1.481e  -8 |  | 2.665e -7 |  | 3.218e  -8 |  | 0.712 |  |
| time |  | 0.053 |  | 6.697e  -9 |  | 1.206e -7 |  | 1.455e  -8 |  | 1.286 |  |
| time + ocd\_vs\_hc |  | 0.053 |  | 3.869e  -9 |  | 6.964e -8 |  | 8.408e  -9 |  | 61.854 |  |
| ocd\_vs\_hc |  | 0.053 |  | 3.189e  -9 |  | 5.740e -8 |  | 6.930e  -9 |  | 1.040 |  |
| time + ocd\_vs\_hc + time  ✻  ocd\_vs\_hc |  | 0.053 |  | 2.738e -10 |  | 4.928e -9 |  | 5.949e -10 |  | 3.177 |  |
|  | | | | | | | | | | | |
|  |  |  |  |  |  |  |  |  |  |  |  |
| --- | --- | --- | --- | --- | --- | --- | --- | --- | --- | --- | --- |
| *Note.*  All models include subject | | | | | | | | | | | |

## Table S22. Airblast expectancy ratings extinction

| Model Comparison | | | | | | | | | | | |
| --- | --- | --- | --- | --- | --- | --- | --- | --- | --- | --- | --- |
| Models | | P(M) | | P(M|data) | | BF M | | BF 10 | | error % | |
| stim |  | 0.053 |  | 0.582 |  | 25.090 |  | 1.000 |  |  |  |
| time + stim |  | 0.053 |  | 0.178 |  | 3.910 |  | 0.306 |  | 2.641 |  |
| stim + ocd\_vs\_hc |  | 0.053 |  | 0.113 |  | 2.284 |  | 0.193 |  | 2.413 |  |
| time + stim + time  ✻  stim |  | 0.053 |  | 0.043 |  | 0.806 |  | 0.074 |  | 2.326 |  |
| time + stim + ocd\_vs\_hc |  | 0.053 |  | 0.037 |  | 0.682 |  | 0.063 |  | 4.775 |  |
| stim + ocd\_vs\_hc + stim  ✻  ocd\_vs\_hc |  | 0.053 |  | 0.019 |  | 0.346 |  | 0.032 |  | 2.763 |  |
| time + stim + ocd\_vs\_hc + time  ✻  stim |  | 0.053 |  | 0.009 |  | 0.159 |  | 0.015 |  | 3.385 |  |
| time + stim + ocd\_vs\_hc + time  ✻  ocd\_vs\_hc |  | 0.053 |  | 0.007 |  | 0.131 |  | 0.012 |  | 5.208 |  |
| time + stim + ocd\_vs\_hc + stim  ✻  ocd\_vs\_hc |  | 0.053 |  | 0.006 |  | 0.101 |  | 0.010 |  | 3.224 |  |
| time + stim + ocd\_vs\_hc + time  ✻  stim + stim  ✻  ocd\_vs\_hc |  | 0.053 |  | 0.003 |  | 0.062 |  | 0.006 |  | 59.092 |  |
| time + stim + ocd\_vs\_hc + time  ✻  stim + time  ✻  ocd\_vs\_hc |  | 0.053 |  | 0.002 |  | 0.032 |  | 0.003 |  | 3.479 |  |
| time + stim + ocd\_vs\_hc + time  ✻  ocd\_vs\_hc + stim  ✻  ocd\_vs\_hc |  | 0.053 |  | 0.001 |  | 0.026 |  | 0.002 |  | 25.145 |  |
| time + stim + ocd\_vs\_hc + time  ✻  stim + time  ✻  ocd\_vs\_hc + stim  ✻  ocd\_vs\_hc |  | 0.053 |  | 2.919e  -4 |  | 0.005 |  | 5.014e  -4 |  | 6.529 |  |
| time + stim + ocd\_vs\_hc + time  ✻  stim + time  ✻  ocd\_vs\_hc + stim  ✻  ocd\_vs\_hc + time  ✻  stim  ✻  ocd\_vs\_hc |  | 0.053 |  | 6.404e  -5 |  | 0.001 |  | 1.100e  -4 |  | 7.075 |  |
| Null model (incl. subject) |  | 0.053 |  | 3.154e -14 |  | 5.677e -13 |  | 5.417e -14 |  | 1.026 |  |
| time |  | 0.053 |  | 8.322e -15 |  | 1.498e -13 |  | 1.429e -14 |  | 3.770 |  |
| ocd\_vs\_hc |  | 0.053 |  | 5.552e -15 |  | 9.994e -14 |  | 9.536e -15 |  | 1.511 |  |
| time + ocd\_vs\_hc |  | 0.053 |  | 1.357e -15 |  | 2.443e -14 |  | 2.331e -15 |  | 1.962 |  |
| time + ocd\_vs\_hc + time  ✻  ocd\_vs\_hc |  | 0.053 |  | 2.574e -16 |  | 4.633e -15 |  | 4.421e -16 |  | 2.619 |  |
|  | | | | | | | | | | | |
|  |  |  |  |  |  |  |  |  |  |  |  |
| --- | --- | --- | --- | --- | --- | --- | --- | --- | --- | --- | --- |
| *Note.*  All models include subject | | | | | | | | | | | |

## Table S23. Shock expectancy ratings conditioning frequentist ANOVAs

| Within Subjects Effects | | | | | | | | | | | | | |
| --- | --- | --- | --- | --- | --- | --- | --- | --- | --- | --- | --- | --- | --- |
| Cases | | Sum of Squares | | df | | Mean Square | | F | | p | | η² | |
| time |  | 4.933 | ᵃ | 3 | ᵃ | 1.644 | ᵃ | 0.433 | ᵃ | 0.730 | ᵃ | 5.068e -4 |  |
| time ✻ ocd\_vs\_hc |  | 22.224 | ᵃ | 3 | ᵃ | 7.408 | ᵃ | 1.949 | ᵃ | 0.122 | ᵃ | 0.002 |  |
| Residuals |  | 1037.454 |  | 273 |  | 3.800 |  |  |  |  |  |  |  |
| stim |  | 4486.720 |  | 1 |  | 4486.720 |  | 550.313 |  | < .001 |  | 0.461 |  |
| stim ✻ ocd\_vs\_hc |  | 28.860 |  | 1 |  | 28.860 |  | 3.540 |  | 0.063 |  | 0.003 |  |
| Residuals |  | 741.925 |  | 91 |  | 8.153 |  |  |  |  |  |  |  |
| time ✻ stim |  | 1705.142 | ᵃ | 3 | ᵃ | 568.381 | ᵃ | 128.286 | ᵃ | < .001 | ᵃ | 0.175 |  |
| time ✻ stim ✻ ocd\_vs\_hc |  | 7.948 | ᵃ | 3 | ᵃ | 2.649 | ᵃ | 0.598 | ᵃ | 0.617 | ᵃ | 8.164e -4 |  |
| Residuals |  | 1209.547 |  | 273 |  | 4.431 |  |  |  |  |  |  |  |
|  | | | | | | | | | | | | | |
|  |  |  |  |  |  |  |  |  |  |  |  |  |  |
| --- | --- | --- | --- | --- | --- | --- | --- | --- | --- | --- | --- | --- | --- |
| *Note.*  Type III Sum of Squares | | | | | | | | | | | | | |
| ᵃ Mauchly's test of sphericity indicates that the assumption of sphericity is violated (p < .05). | | | | | | | | | | | | | |

| Between Subjects Effects | | | | | | | | | | | | | |
| --- | --- | --- | --- | --- | --- | --- | --- | --- | --- | --- | --- | --- | --- |
| Cases | | Sum of Squares | | df | | Mean Square | | F | | p | | η² | |
| ocd\_vs\_hc |  | 4.172 |  | 1 |  | 4.172 |  | 0.780 |  | 0.379 |  | 4.285e -4 |  |
| Residuals |  | 486.452 |  | 91 |  | 5.346 |  |  |  |  |  |  |  |
|  | | | | | | | | | | | | | |
|  |  |  |  |  |  |  |  |  |  |  |  |  |  |
| --- | --- | --- | --- | --- | --- | --- | --- | --- | --- | --- | --- | --- | --- |
| *Note.*  Type III Sum of Squares | | | | | | | | | | | | | |

### Descriptives

| Descriptives | | | | | | | | | | | |
| --- | --- | --- | --- | --- | --- | --- | --- | --- | --- | --- | --- |
| time | | stim | | ocd\_vs\_hc | | Mean | | SD | | N | |
| 1 |  | min |  | 0 |  | 4.536 |  | 2.434 |  | 56 |  |
|  |  |  |  | 1 |  | 4.135 |  | 2.850 |  | 37 |  |
|  |  | shock |  | 0 |  | 4.571 |  | 2.327 |  | 56 |  |
|  |  |  |  | 1 |  | 3.676 |  | 2.416 |  | 37 |  |
| 2 |  | min |  | 0 |  | 1.036 |  | 1.907 |  | 56 |  |
|  |  |  |  | 1 |  | 2.000 |  | 2.934 |  | 37 |  |
|  |  | shock |  | 0 |  | 7.393 |  | 2.506 |  | 56 |  |
|  |  |  |  | 1 |  | 6.973 |  | 3.078 |  | 37 |  |
| 3 |  | min |  | 0 |  | 0.714 |  | 1.724 |  | 56 |  |
|  |  |  |  | 1 |  | 0.919 |  | 1.816 |  | 37 |  |
|  |  | shock |  | 0 |  | 8.464 |  | 1.537 |  | 56 |  |
|  |  |  |  | 1 |  | 7.649 |  | 2.720 |  | 37 |  |
| 4 |  | min |  | 0 |  | 0.446 |  | 1.374 |  | 56 |  |
|  |  |  |  | 1 |  | 0.676 |  | 1.582 |  | 37 |  |
|  |  | shock |  | 0 |  | 7.982 |  | 1.555 |  | 56 |  |
|  |  |  |  | 1 |  | 7.892 |  | 2.025 |  | 37 |  |
|  | | | | | | | | | | | |

## Table S24. Airblast expectancy ratings conditioning frequentist ANOVAs

| Within Subjects Effects | | | | | | | | | | | | | |
| --- | --- | --- | --- | --- | --- | --- | --- | --- | --- | --- | --- | --- | --- |
| Cases | | Sum of Squares | | df | | Mean Square | | F | | p | | η² | |
| time |  | 14.728 | ᵃ | 3 | ᵃ | 4.909 | ᵃ | 1.366 | ᵃ | 0.253 | ᵃ | 0.002 |  |
| time ✻ ocd\_vs\_hc |  | 19.018 | ᵃ | 3 | ᵃ | 6.339 | ᵃ | 1.764 | ᵃ | 0.154 | ᵃ | 0.002 |  |
| Residuals |  | 980.990 |  | 273 |  | 3.593 |  |  |  |  |  |  |  |
| stim |  | 4357.964 |  | 1 |  | 4357.964 |  | 527.276 |  | < .001 |  | 0.468 |  |
| stim ✻ ocd\_vs\_hc |  | 0.953 |  | 1 |  | 0.953 |  | 0.115 |  | 0.735 |  | 1.022e -4 |  |
| Residuals |  | 752.120 |  | 91 |  | 8.265 |  |  |  |  |  |  |  |
| time ✻ stim |  | 1620.255 | ᵃ | 3 | ᵃ | 540.085 | ᵃ | 156.623 | ᵃ | < .001 | ᵃ | 0.174 |  |
| time ✻ stim ✻ ocd\_vs\_hc |  | 36.621 | ᵃ | 3 | ᵃ | 12.207 | ᵃ | 3.540 | ᵃ | 0.015 | ᵃ | 0.004 |  |
| Residuals |  | 941.392 |  | 273 |  | 3.448 |  |  |  |  |  |  |  |
|  | | | | | | | | | | | | | |
|  |  |  |  |  |  |  |  |  |  |  |  |  |  |
| --- | --- | --- | --- | --- | --- | --- | --- | --- | --- | --- | --- | --- | --- |
| *Note.*  Type III Sum of Squares | | | | | | | | | | | | | |
| ᵃ Mauchly's test of sphericity indicates that the assumption of sphericity is violated (p < .05). | | | | | | | | | | | | | |

| Between Subjects Effects | | | | | | | | | | | | | |
| --- | --- | --- | --- | --- | --- | --- | --- | --- | --- | --- | --- | --- | --- |
| Cases | | Sum of Squares | | df | | Mean Square | | F | | p | | η² | |
| ocd\_vs\_hc |  | 9.917 |  | 1 |  | 9.917 |  | 1.538 |  | 0.218 |  | 0.001 |  |
| Residuals |  | 586.795 |  | 91 |  | 6.448 |  |  |  |  |  |  |  |
|  | | | | | | | | | | | | | |
|  |  |  |  |  |  |  |  |  |  |  |  |  |  |
| --- | --- | --- | --- | --- | --- | --- | --- | --- | --- | --- | --- | --- | --- |
| *Note.*  Type III Sum of Squares | | | | | | | | | | | | | |

### Descriptives

| Descriptives | | | | | | | | | | | |
| --- | --- | --- | --- | --- | --- | --- | --- | --- | --- | --- | --- |
| time | | stim | | ocd\_vs\_hc | | Mean | | SD | | N | |
| 1 |  | min |  | 0 |  | 5.107 |  | 2.432 |  | 56 |  |
|  |  |  |  | 1 |  | 3.730 |  | 2.684 |  | 37 |  |
|  |  | shock |  | 0 |  | 4.179 |  | 2.046 |  | 56 |  |
|  |  |  |  | 1 |  | 4.189 |  | 2.612 |  | 37 |  |
| 2 |  | min |  | 0 |  | 1.232 |  | 2.166 |  | 56 |  |
|  |  |  |  | 1 |  | 1.676 |  | 2.473 |  | 37 |  |
|  |  | shock |  | 0 |  | 7.482 |  | 1.868 |  | 56 |  |
|  |  |  |  | 1 |  | 7.514 |  | 2.388 |  | 37 |  |
| 3 |  | min |  | 0 |  | 0.857 |  | 2.169 |  | 56 |  |
|  |  |  |  | 1 |  | 0.946 |  | 1.957 |  | 37 |  |
|  |  | shock |  | 0 |  | 8.196 |  | 1.313 |  | 56 |  |
|  |  |  |  | 1 |  | 7.676 |  | 2.148 |  | 37 |  |
| 4 |  | min |  | 0 |  | 0.536 |  | 1.673 |  | 56 |  |
|  |  |  |  | 1 |  | 0.730 |  | 1.967 |  | 37 |  |
|  |  | shock |  | 0 |  | 7.946 |  | 1.313 |  | 56 |  |
|  |  |  |  | 1 |  | 7.189 |  | 2.787 |  | 37 |  |
|  | | | | | | | | | | | |

## Table S25. Shock expectancy ratings extinction frequentist ANOVAs

| Within Subjects Effects | | | | | | | | | | | | | |
| --- | --- | --- | --- | --- | --- | --- | --- | --- | --- | --- | --- | --- | --- |
| Cases | | Sum of Squares | | df | | Mean Square | | F | | p | | η² | |
| time |  | 5.404 |  | 1 |  | 5.404 |  | 4.767 |  | 0.032 |  | 0.004 |  |
| time ✻ ocd\_vs\_hc |  | 0.630 |  | 1 |  | 0.630 |  | 0.556 |  | 0.458 |  | 4.504e -4 |  |
| Residuals |  | 103.165 |  | 91 |  | 1.134 |  |  |  |  |  |  |  |
| stim |  | 79.765 |  | 1 |  | 79.765 |  | 19.943 |  | < .001 |  | 0.057 |  |
| stim ✻ ocd\_vs\_hc |  | 0.066 |  | 1 |  | 0.066 |  | 0.016 |  | 0.898 |  | 4.693e -5 |  |
| Residuals |  | 363.967 |  | 91 |  | 4.000 |  |  |  |  |  |  |  |
| time ✻ stim |  | 0.456 |  | 1 |  | 0.456 |  | 0.606 |  | 0.438 |  | 3.260e -4 |  |
| time ✻ stim ✻ ocd\_vs\_hc |  | 0.177 |  | 1 |  | 0.177 |  | 0.235 |  | 0.629 |  | 1.262e -4 |  |
| Residuals |  | 68.469 |  | 91 |  | 0.752 |  |  |  |  |  |  |  |
|  | | | | | | | | | | | | | |
|  |  |  |  |  |  |  |  |  |  |  |  |  |  |
| --- | --- | --- | --- | --- | --- | --- | --- | --- | --- | --- | --- | --- | --- |
| *Note.*  Type III Sum of Squares | | | | | | | | | | | | | |

| Between Subjects Effects | | | | | | | | | | | | | |
| --- | --- | --- | --- | --- | --- | --- | --- | --- | --- | --- | --- | --- | --- |
| Cases | | Sum of Squares | | df | | Mean Square | | F | | p | | η² | |
| ocd\_vs\_hc |  | 0.241 |  | 1 |  | 0.241 |  | 0.028 |  | 0.867 |  | 1.722e -4 |  |
| Residuals |  | 777.071 |  | 91 |  | 8.539 |  |  |  |  |  |  |  |
|  | | | | | | | | | | | | | |
|  |  |  |  |  |  |  |  |  |  |  |  |  |  |
| --- | --- | --- | --- | --- | --- | --- | --- | --- | --- | --- | --- | --- | --- |
| *Note.*  Type III Sum of Squares | | | | | | | | | | | | | |

### Descriptives

| Descriptives | | | | | | | | | | | |
| --- | --- | --- | --- | --- | --- | --- | --- | --- | --- | --- | --- |
| time | | stim | | ocd\_vs\_hc | | Mean | | SD | | N | |
| 1 |  | min |  | 0 |  | 0.393 |  | 1.397 |  | 56 |  |
|  |  |  |  | 1 |  | 0.432 |  | 1.365 |  | 37 |  |
|  |  | shock |  | 0 |  | 1.482 |  | 2.635 |  | 56 |  |
|  |  |  |  | 1 |  | 1.378 |  | 2.215 |  | 37 |  |
| 2 |  | min |  | 0 |  | 0.179 |  | 0.636 |  | 56 |  |
|  |  |  |  | 1 |  | 0.297 |  | 1.051 |  | 37 |  |
|  |  | shock |  | 0 |  | 1.036 |  | 2.381 |  | 56 |  |
|  |  |  |  | 1 |  | 1.189 |  | 2.390 |  | 37 |  |
|  | | | | | | | | | | | |

## Table S26. Shock expectancy ratings extinction frequentist ANOVAs

| Within Subjects Effects | | | | | | | | | | | | | |
| --- | --- | --- | --- | --- | --- | --- | --- | --- | --- | --- | --- | --- | --- |
| Cases | | Sum of Squares | | df | | Mean Square | | F | | p | | η² | |
| time |  | 9.200 |  | 1 |  | 9.200 |  | 5.615 |  | 0.020 |  | 0.004 |  |
| time ✻ ocd\_vs\_hc |  | 2.050 |  | 1 |  | 2.050 |  | 1.251 |  | 0.266 |  | 8.608e -4 |  |
| Residuals |  | 149.111 |  | 91 |  | 1.639 |  |  |  |  |  |  |  |
| stim |  | 280.313 |  | 1 |  | 280.313 |  | 32.484 |  | < .001 |  | 0.118 |  |
| stim ✻ ocd\_vs\_hc |  | 9.458e -4 |  | 1 |  | 9.458e -4 |  | 1.096e -4 |  | 0.992 |  | 3.971e -7 |  |
| Residuals |  | 785.257 |  | 91 |  | 8.629 |  |  |  |  |  |  |  |
| time ✻ stim |  | 4.407 |  | 1 |  | 4.407 |  | 3.075 |  | 0.083 |  | 0.002 |  |
| time ✻ stim ✻ ocd\_vs\_hc |  | 0.697 |  | 1 |  | 0.697 |  | 0.486 |  | 0.487 |  | 2.927e -4 |  |
| Residuals |  | 130.421 |  | 91 |  | 1.433 |  |  |  |  |  |  |  |
|  | | | | | | | | | | | | | |
|  |  |  |  |  |  |  |  |  |  |  |  |  |  |
| --- | --- | --- | --- | --- | --- | --- | --- | --- | --- | --- | --- | --- | --- |
| *Note.*  Type III Sum of Squares | | | | | | | | | | | | | |

| Between Subjects Effects | | | | | | | | | | | | | |
| --- | --- | --- | --- | --- | --- | --- | --- | --- | --- | --- | --- | --- | --- |
| Cases | | Sum of Squares | | df | | Mean Square | | F | | p | | η² | |
| ocd\_vs\_hc |  | 0.462 |  | 1 |  | 0.462 |  | 0.041 |  | 0.839 |  | 1.942e -4 |  |
| Residuals |  | 1019.570 |  | 91 |  | 11.204 |  |  |  |  |  |  |  |
|  | | | | | | | | | | | | | |
|  |  |  |  |  |  |  |  |  |  |  |  |  |  |
| --- | --- | --- | --- | --- | --- | --- | --- | --- | --- | --- | --- | --- | --- |
| *Note.*  Type III Sum of Squares | | | | | | | | | | | | | |

### Descriptives

| Descriptives | | | | | | | | | | | |
| --- | --- | --- | --- | --- | --- | --- | --- | --- | --- | --- | --- |
| time | | stim | | ocd\_vs\_hc | | Mean | | SD | | N | |
| 1 |  | min |  | 0 |  | 0.375 |  | 1.229 |  | 56 |  |
|  |  |  |  | 1 |  | 0.514 |  | 1.387 |  | 37 |  |
|  |  | shock |  | 0 |  | 2.286 |  | 3.251 |  | 56 |  |
|  |  |  |  | 1 |  | 2.595 |  | 3.270 |  | 37 |  |
| 2 |  | min |  | 0 |  | 0.339 |  | 1.269 |  | 56 |  |
|  |  |  |  | 1 |  | 0.351 |  | 1.296 |  | 37 |  |
|  |  | shock |  | 0 |  | 1.982 |  | 3.171 |  | 56 |  |
|  |  |  |  | 1 |  | 1.811 |  | 2.706 |  | 37 |  |
|  | | | | | | | | | | | |
