# Supplementary material for: Examining Differences in Fear Learning in Patients With Obsessive-Compulsive Disorder With Pupillometry, Startle Electromyography and Skin Conductance Responses
Source: Front Psychiatry. 2021 Oct 1;12:730742. doi: 10.3389/fpsyt.2021.730742 (PMC8517251; doi:10.3389/fpsyt.2021.730742)
Supplement: Supplementary file 1 [file Data_Sheet_1.ZIP › supplementary tables S27 - S41.html]

JASP 


# sample B recall and reinstatement - tables S27 - S41

## Table S27. SCR recall (cs+shock) frequentist ANOVAs

| Within Subjects Effects | | | | | | | | | | | | | |
| --- | --- | --- | --- | --- | --- | --- | --- | --- | --- | --- | --- | --- | --- |
| Cases | | Sum of Squares | | df | | Mean Square | | F | | p | | η² | |
| time |  | 10.066 |  | 1 |  | 10.066 |  | 17.251 |  | < .001 |  | 0.058 |  |
| time ✻ ocd\_vs\_hc |  | 0.747 |  | 1 |  | 0.747 |  | 1.281 |  | 0.262 |  | 0.004 |  |
| Residuals |  | 38.513 |  | 66 |  | 0.584 |  |  |  |  |  |  |  |
| stim |  | 9.087 |  | 1 |  | 9.087 |  | 22.876 |  | < .001 |  | 0.052 |  |
| stim ✻ ocd\_vs\_hc |  | 0.099 |  | 1 |  | 0.099 |  | 0.249 |  | 0.619 |  | 5.662e -4 |  |
| Residuals |  | 26.217 |  | 66 |  | 0.397 |  |  |  |  |  |  |  |
| time ✻ stim |  | 1.555 |  | 1 |  | 1.555 |  | 4.269 |  | 0.043 |  | 0.009 |  |
| time ✻ stim ✻ ocd\_vs\_hc |  | 0.648 |  | 1 |  | 0.648 |  | 1.780 |  | 0.187 |  | 0.004 |  |
| Residuals |  | 24.037 |  | 66 |  | 0.364 |  |  |  |  |  |  |  |
|  | | | | | | | | | | | | | |
|  |  |  |  |  |  |  |  |  |  |  |  |  |  |
| --- | --- | --- | --- | --- | --- | --- | --- | --- | --- | --- | --- | --- | --- |
| *Note.*  Type III Sum of Squares | | | | | | | | | | | | | |

| Between Subjects Effects | | | | | | | | | | | | | |
| --- | --- | --- | --- | --- | --- | --- | --- | --- | --- | --- | --- | --- | --- |
| Cases | | Sum of Squares | | df | | Mean Square | | F | | p | | η² | |
| ocd\_vs\_hc |  | 0.003 |  | 1 |  | 0.003 |  | 0.003 |  | 0.956 |  | 1.659e -5 |  |
| Residuals |  | 63.791 |  | 66 |  | 0.967 |  |  |  |  |  |  |  |
|  | | | | | | | | | | | | | |
|  |  |  |  |  |  |  |  |  |  |  |  |  |  |
| --- | --- | --- | --- | --- | --- | --- | --- | --- | --- | --- | --- | --- | --- |
| *Note.*  Type III Sum of Squares | | | | | | | | | | | | | |

### Descriptives

| Descriptives | | | | | | | | | | | |
| --- | --- | --- | --- | --- | --- | --- | --- | --- | --- | --- | --- |
| time | | stim | | ocd\_vs\_hc | | Mean | | SD | | N | |
| 1 |  | min |  | 0 |  | 0.040 |  | 0.601 |  | 41 |  |
|  |  |  |  | 1 |  | 0.292 |  | 0.904 |  | 27 |  |
|  |  | shock |  | 0 |  | 0.707 |  | 1.074 |  | 41 |  |
|  |  |  |  | 1 |  | 0.682 |  | 0.929 |  | 27 |  |
| 2 |  | min |  | 0 |  | 0.008 |  | 0.526 |  | 41 |  |
|  |  |  |  | 1 |  | -0.153 |  | 0.370 |  | 27 |  |
|  |  | shock |  | 0 |  | 0.166 |  | 0.762 |  | 41 |  |
|  |  |  |  | 1 |  | 0.127 |  | 0.633 |  | 27 |  |
|  | | | | | | | | | | | |

#### Descriptives plots

##### ocd\_vs\_hc: 0

##### ocd\_vs\_hc: 1

## Table S28. SCR recall (cs+air) frequentist ANOVAs

| Within Subjects Effects | | | | | | | | | | | | | |
| --- | --- | --- | --- | --- | --- | --- | --- | --- | --- | --- | --- | --- | --- |
| Cases | | Sum of Squares | | df | | Mean Square | | F | | p | | η² | |
| time |  | 6.092 |  | 1 |  | 6.092 |  | 12.460 |  | < .001 |  | 0.049 |  |
| time ✻ ocd\_vs\_hc |  | 1.177 |  | 1 |  | 1.177 |  | 2.407 |  | 0.126 |  | 0.010 |  |
| Residuals |  | 32.267 |  | 66 |  | 0.489 |  |  |  |  |  |  |  |
| stim |  | 0.225 |  | 1 |  | 0.225 |  | 0.677 |  | 0.414 |  | 0.002 |  |
| stim ✻ ocd\_vs\_hc |  | 0.009 |  | 1 |  | 0.009 |  | 0.026 |  | 0.872 |  | 7.068e -5 |  |
| Residuals |  | 21.925 |  | 66 |  | 0.332 |  |  |  |  |  |  |  |
| time ✻ stim |  | 0.294 |  | 1 |  | 0.294 |  | 1.109 |  | 0.296 |  | 0.002 |  |
| time ✻ stim ✻ ocd\_vs\_hc |  | 0.342 |  | 1 |  | 0.342 |  | 1.290 |  | 0.260 |  | 0.003 |  |
| Residuals |  | 17.497 |  | 66 |  | 0.265 |  |  |  |  |  |  |  |
|  | | | | | | | | | | | | | |
|  |  |  |  |  |  |  |  |  |  |  |  |  |  |
| --- | --- | --- | --- | --- | --- | --- | --- | --- | --- | --- | --- | --- | --- |
| *Note.*  Type III Sum of Squares | | | | | | | | | | | | | |

| Between Subjects Effects | | | | | | | | | | | | | |
| --- | --- | --- | --- | --- | --- | --- | --- | --- | --- | --- | --- | --- | --- |
| Cases | | Sum of Squares | | df | | Mean Square | | F | | p | | η² | |
| ocd\_vs\_hc |  | 0.213 |  | 1 |  | 0.213 |  | 0.324 |  | 0.571 |  | 0.002 |  |
| Residuals |  | 43.514 |  | 66 |  | 0.659 |  |  |  |  |  |  |  |
|  | | | | | | | | | | | | | |
|  |  |  |  |  |  |  |  |  |  |  |  |  |  |
| --- | --- | --- | --- | --- | --- | --- | --- | --- | --- | --- | --- | --- | --- |
| *Note.*  Type III Sum of Squares | | | | | | | | | | | | | |

### Descriptives

| Descriptives | | | | | | | | | | | |
| --- | --- | --- | --- | --- | --- | --- | --- | --- | --- | --- | --- |
| time | | stim | | ocd\_vs\_hc | | Mean | | SD | | N | |
| 1 |  | min |  | 0 |  | 0.040 |  | 0.601 |  | 41 |  |
|  |  |  |  | 1 |  | 0.292 |  | 0.904 |  | 27 |  |
|  |  | shock |  | 0 |  | 0.227 |  | 0.703 |  | 41 |  |
|  |  |  |  | 1 |  | 0.357 |  | 0.834 |  | 27 |  |
| 2 |  | min |  | 0 |  | 0.008 |  | 0.526 |  | 41 |  |
|  |  |  |  | 1 |  | -0.153 |  | 0.370 |  | 27 |  |
|  |  | shock |  | 0 |  | -0.084 |  | 0.693 |  | 41 |  |
|  |  |  |  | 1 |  | -0.078 |  | 0.548 |  | 27 |  |
|  | | | | | | | | | | | |

#### Descriptives plots

##### ocd\_vs\_hc: 0

##### ocd\_vs\_hc: 1

## Table S29. SCR reinstatement (cs+shock) frequentist ANOVAs

| Within Subjects Effects | | | | | | | | | | | | | |
| --- | --- | --- | --- | --- | --- | --- | --- | --- | --- | --- | --- | --- | --- |
| Cases | | Sum of Squares | | df | | Mean Square | | F | | p | | η² | |
| time |  | 4.216 |  | 1 |  | 4.216 |  | 7.082 |  | 0.010 |  | 0.030 |  |
| time ✻ ocd\_vs\_hc |  | 0.048 |  | 1 |  | 0.048 |  | 0.080 |  | 0.778 |  | 3.361e -4 |  |
| Residuals |  | 31.551 |  | 53 |  | 0.595 |  |  |  |  |  |  |  |
| stim |  | 0.806 |  | 1 |  | 0.806 |  | 1.911 |  | 0.173 |  | 0.006 |  |
| stim ✻ ocd\_vs\_hc |  | 0.003 |  | 1 |  | 0.003 |  | 0.007 |  | 0.934 |  | 2.045e -5 |  |
| Residuals |  | 22.367 |  | 53 |  | 0.422 |  |  |  |  |  |  |  |
| time ✻ stim |  | 2.884 |  | 1 |  | 2.884 |  | 3.877 |  | 0.054 |  | 0.020 |  |
| time ✻ stim ✻ ocd\_vs\_hc |  | 0.623 |  | 1 |  | 0.623 |  | 0.838 |  | 0.364 |  | 0.004 |  |
| Residuals |  | 39.428 |  | 53 |  | 0.744 |  |  |  |  |  |  |  |
|  | | | | | | | | | | | | | |
|  |  |  |  |  |  |  |  |  |  |  |  |  |  |
| --- | --- | --- | --- | --- | --- | --- | --- | --- | --- | --- | --- | --- | --- |
| *Note.*  Type III Sum of Squares | | | | | | | | | | | | | |

| Between Subjects Effects | | | | | | | | | | | | | |
| --- | --- | --- | --- | --- | --- | --- | --- | --- | --- | --- | --- | --- | --- |
| Cases | | Sum of Squares | | df | | Mean Square | | F | | p | | η² | |
| ocd\_vs\_hc |  | 2.323e -4 |  | 1 |  | 2.323e -4 |  | 3.059e -4 |  | 0.986 |  | 1.634e -6 |  |
| Residuals |  | 40.245 |  | 53 |  | 0.759 |  |  |  |  |  |  |  |
|  | | | | | | | | | | | | | |
|  |  |  |  |  |  |  |  |  |  |  |  |  |  |
| --- | --- | --- | --- | --- | --- | --- | --- | --- | --- | --- | --- | --- | --- |
| *Note.*  Type III Sum of Squares | | | | | | | | | | | | | |

### Descriptives

| Descriptives | | | | | | | | | | | |
| --- | --- | --- | --- | --- | --- | --- | --- | --- | --- | --- | --- |
| time | | stim | | ocd\_vs\_hc | | Mean | | SD | | N | |
| 1 |  | min |  | 0 |  | 0.077 |  | 0.757 |  | 30 |  |
|  |  |  |  | 1 |  | -0.068 |  | 0.544 |  | 25 |  |
|  |  | shock |  | 0 |  | 0.315 |  | 0.898 |  | 30 |  |
|  |  |  |  | 1 |  | 0.397 |  | 1.060 |  | 25 |  |
| 2 |  | min |  | 0 |  | -0.107 |  | 0.661 |  | 30 |  |
|  |  |  |  | 1 |  | 0.020 |  | 1.113 |  | 25 |  |
|  |  | shock |  | 0 |  | -0.116 |  | 0.684 |  | 30 |  |
|  |  |  |  | 1 |  | -0.188 |  | 0.387 |  | 25 |  |
|  | | | | | | | | | | | |

#### Descriptives plots

##### ocd\_vs\_hc: 0

##### ocd\_vs\_hc: 1

## Table S30. SCR reinstatement (cs+air) frequentist ANOVAs

| Within Subjects Effects | | | | | | | | | | | | | |
| --- | --- | --- | --- | --- | --- | --- | --- | --- | --- | --- | --- | --- | --- |
| Cases | | Sum of Squares | | df | | Mean Square | | F | | p | | η² | |
| time |  | 0.132 |  | 1 |  | 0.132 |  | 0.130 |  | 0.720 |  | 7.308e -4 |  |
| time ✻ ocd\_vs\_hc |  | 0.515 |  | 1 |  | 0.515 |  | 0.504 |  | 0.481 |  | 0.003 |  |
| Residuals |  | 55.197 |  | 54 |  | 1.022 |  |  |  |  |  |  |  |
| stim |  | 0.117 |  | 1 |  | 0.117 |  | 0.162 |  | 0.689 |  | 6.480e -4 |  |
| stim ✻ ocd\_vs\_hc |  | 1.038 |  | 1 |  | 1.038 |  | 1.430 |  | 0.237 |  | 0.006 |  |
| Residuals |  | 39.221 |  | 54 |  | 0.726 |  |  |  |  |  |  |  |
| time ✻ stim |  | 0.861 |  | 1 |  | 0.861 |  | 1.236 |  | 0.271 |  | 0.005 |  |
| time ✻ stim ✻ ocd\_vs\_hc |  | 1.517 |  | 1 |  | 1.517 |  | 2.179 |  | 0.146 |  | 0.008 |  |
| Residuals |  | 37.595 |  | 54 |  | 0.696 |  |  |  |  |  |  |  |
|  | | | | | | | | | | | | | |
|  |  |  |  |  |  |  |  |  |  |  |  |  |  |
| --- | --- | --- | --- | --- | --- | --- | --- | --- | --- | --- | --- | --- | --- |
| *Note.*  Type III Sum of Squares | | | | | | | | | | | | | |

| Between Subjects Effects | | | | | | | | | | | | | |
| --- | --- | --- | --- | --- | --- | --- | --- | --- | --- | --- | --- | --- | --- |
| Cases | | Sum of Squares | | df | | Mean Square | | F | | p | | η² | |
| ocd\_vs\_hc |  | 0.006 |  | 1 |  | 0.006 |  | 0.007 |  | 0.932 |  | 3.354e -5 |  |
| Residuals |  | 44.933 |  | 54 |  | 0.832 |  |  |  |  |  |  |  |
|  | | | | | | | | | | | | | |
|  |  |  |  |  |  |  |  |  |  |  |  |  |  |
| --- | --- | --- | --- | --- | --- | --- | --- | --- | --- | --- | --- | --- | --- |
| *Note.*  Type III Sum of Squares | | | | | | | | | | | | | |

### Descriptives

| Descriptives | | | | | | | | | | | |
| --- | --- | --- | --- | --- | --- | --- | --- | --- | --- | --- | --- |
| time | | stim | | ocd\_vs\_hc | | Mean | | SD | | N | |
| 1 |  | min |  | 0 |  | 0.320 |  | 1.543 |  | 31 |  |
|  |  |  |  | 1 |  | -0.068 |  | 0.544 |  | 25 |  |
|  |  | shock |  | 0 |  | -0.153 |  | 0.493 |  | 31 |  |
|  |  |  |  | 1 |  | 0.063 |  | 0.961 |  | 25 |  |
| 2 |  | min |  | 0 |  | -0.116 |  | 0.651 |  | 31 |  |
|  |  |  |  | 1 |  | 0.020 |  | 1.113 |  | 25 |  |
|  |  | shock |  | 0 |  | -0.008 |  | 0.591 |  | 31 |  |
|  |  |  |  | 1 |  | 0.070 |  | 0.818 |  | 25 |  |
|  | | | | | | | | | | | |

#### Descriptives plots

##### ocd\_vs\_hc: 0

##### ocd\_vs\_hc: 1

## Table S31. Pupil dilation recall (cs+shock) frequentist ANOVAs

| Within Subjects Effects | | | | | | | | | | | | | |
| --- | --- | --- | --- | --- | --- | --- | --- | --- | --- | --- | --- | --- | --- |
| Cases | | Sum of Squares | | df | | Mean Square | | F | | p | | η² | |
| time |  | 4.488 |  | 1 |  | 4.488 |  | 18.072 |  | < .001 |  | 0.059 |  |
| time ✻ ocd\_vs\_hc |  | 0.158 |  | 1 |  | 0.158 |  | 0.638 |  | 0.428 |  | 0.002 |  |
| Residuals |  | 14.154 |  | 57 |  | 0.248 |  |  |  |  |  |  |  |
| stim |  | 4.060 |  | 1 |  | 4.060 |  | 8.954 |  | 0.004 |  | 0.053 |  |
| stim ✻ ocd\_vs\_hc |  | 0.541 |  | 1 |  | 0.541 |  | 1.194 |  | 0.279 |  | 0.007 |  |
| Residuals |  | 25.848 |  | 57 |  | 0.453 |  |  |  |  |  |  |  |
| time ✻ stim |  | 0.370 |  | 1 |  | 0.370 |  | 1.376 |  | 0.246 |  | 0.005 |  |
| time ✻ stim ✻ ocd\_vs\_hc |  | 0.202 |  | 1 |  | 0.202 |  | 0.751 |  | 0.390 |  | 0.003 |  |
| Residuals |  | 15.310 |  | 57 |  | 0.269 |  |  |  |  |  |  |  |
|  | | | | | | | | | | | | | |
|  |  |  |  |  |  |  |  |  |  |  |  |  |  |
| --- | --- | --- | --- | --- | --- | --- | --- | --- | --- | --- | --- | --- | --- |
| *Note.*  Type III Sum of Squares | | | | | | | | | | | | | |

| Between Subjects Effects | | | | | | | | | | | | | |
| --- | --- | --- | --- | --- | --- | --- | --- | --- | --- | --- | --- | --- | --- |
| Cases | | Sum of Squares | | df | | Mean Square | | F | | p | | η² | |
| ocd\_vs\_hc |  | 0.096 |  | 1 |  | 0.096 |  | 0.492 |  | 0.486 |  | 0.001 |  |
| Residuals |  | 11.098 |  | 57 |  | 0.195 |  |  |  |  |  |  |  |
|  | | | | | | | | | | | | | |
|  |  |  |  |  |  |  |  |  |  |  |  |  |  |
| --- | --- | --- | --- | --- | --- | --- | --- | --- | --- | --- | --- | --- | --- |
| *Note.*  Type III Sum of Squares | | | | | | | | | | | | | |

### Descriptives

| Descriptives | | | | | | | | | | | |
| --- | --- | --- | --- | --- | --- | --- | --- | --- | --- | --- | --- |
| time | | stim | | ocd\_vs\_hc | | Mean | | SD | | N | |
| 1 |  | min |  | 0 |  | -0.033 |  | 0.627 |  | 34 |  |
|  |  |  |  | 1 |  | -0.177 |  | 0.492 |  | 25 |  |
|  |  | shock |  | 0 |  | 0.157 |  | 0.453 |  | 34 |  |
|  |  |  |  | 1 |  | 0.324 |  | 0.605 |  | 25 |  |
| 2 |  | min |  | 0 |  | -0.239 |  | 0.426 |  | 34 |  |
|  |  |  |  | 1 |  | -0.369 |  | 0.435 |  | 25 |  |
|  |  | shock |  | 0 |  | -0.091 |  | 0.578 |  | 34 |  |
|  |  |  |  | 1 |  | -0.146 |  | 0.662 |  | 25 |  |
|  | | | | | | | | | | | |

#### Descriptives plots

##### ocd\_vs\_hc: 0

##### ocd\_vs\_hc: 1

## Table S32. Pupil dilation recall (cs+air) frequentist ANOVAs

| Within Subjects Effects | | | | | | | | | | | | | |
| --- | --- | --- | --- | --- | --- | --- | --- | --- | --- | --- | --- | --- | --- |
| Cases | | Sum of Squares | | df | | Mean Square | | F | | p | | η² | |
| time |  | 3.490 |  | 1 |  | 3.490 |  | 11.981 |  | 0.001 |  | 0.040 |  |
| time ✻ ocd\_vs\_hc |  | 0.006 |  | 1 |  | 0.006 |  | 0.019 |  | 0.891 |  | 6.315e -5 |  |
| Residuals |  | 16.605 |  | 57 |  | 0.291 |  |  |  |  |  |  |  |
| stim |  | 5.941 |  | 1 |  | 5.941 |  | 17.013 |  | < .001 |  | 0.068 |  |
| stim ✻ ocd\_vs\_hc |  | 1.268 |  | 1 |  | 1.268 |  | 3.630 |  | 0.062 |  | 0.014 |  |
| Residuals |  | 19.904 |  | 57 |  | 0.349 |  |  |  |  |  |  |  |
| time ✻ stim |  | 0.128 |  | 1 |  | 0.128 |  | 0.520 |  | 0.474 |  | 0.001 |  |
| time ✻ stim ✻ ocd\_vs\_hc |  | 5.390e -4 |  | 1 |  | 5.390e -4 |  | 0.002 |  | 0.963 |  | 6.161e -6 |  |
| Residuals |  | 14.025 |  | 57 |  | 0.246 |  |  |  |  |  |  |  |
|  | | | | | | | | | | | | | |
|  |  |  |  |  |  |  |  |  |  |  |  |  |  |
| --- | --- | --- | --- | --- | --- | --- | --- | --- | --- | --- | --- | --- | --- |
| *Note.*  Type III Sum of Squares | | | | | | | | | | | | | |

| Between Subjects Effects | | | | | | | | | | | | | |
| --- | --- | --- | --- | --- | --- | --- | --- | --- | --- | --- | --- | --- | --- |
| Cases | | Sum of Squares | | df | | Mean Square | | F | | p | | η² | |
| ocd\_vs\_hc |  | 0.007 |  | 1 |  | 0.007 |  | 0.014 |  | 0.906 |  | 7.443e -5 |  |
| Residuals |  | 26.105 |  | 57 |  | 0.458 |  |  |  |  |  |  |  |
|  | | | | | | | | | | | | | |
|  |  |  |  |  |  |  |  |  |  |  |  |  |  |
| --- | --- | --- | --- | --- | --- | --- | --- | --- | --- | --- | --- | --- | --- |
| *Note.*  Type III Sum of Squares | | | | | | | | | | | | | |

### Descriptives

| Descriptives | | | | | | | | | | | |
| --- | --- | --- | --- | --- | --- | --- | --- | --- | --- | --- | --- |
| time | | stim | | ocd\_vs\_hc | | Mean | | SD | | N | |
| 1 |  | min |  | 0 |  | -0.033 |  | 0.627 |  | 34 |  |
|  |  |  |  | 1 |  | -0.177 |  | 0.492 |  | 25 |  |
|  |  | shock |  | 0 |  | 0.190 |  | 0.620 |  | 34 |  |
|  |  |  |  | 1 |  | 0.336 |  | 0.697 |  | 25 |  |
| 2 |  | min |  | 0 |  | -0.239 |  | 0.426 |  | 34 |  |
|  |  |  |  | 1 |  | -0.369 |  | 0.435 |  | 25 |  |
|  |  | shock |  | 0 |  | -0.116 |  | 0.729 |  | 34 |  |
|  |  |  |  | 1 |  | 0.056 |  | 0.478 |  | 25 |  |
|  | | | | | | | | | | | |

#### Descriptives plots

##### ocd\_vs\_hc: 0

##### ocd\_vs\_hc: 1

## Table S33. Pupil dilation reinstatement (cs+shock) frequentist ANOVAs

| Within Subjects Effects | | | | | | | | | | | | | |
| --- | --- | --- | --- | --- | --- | --- | --- | --- | --- | --- | --- | --- | --- |
| Cases | | Sum of Squares | | df | | Mean Square | | F | | p | | η² | |
| time |  | 2.079 |  | 1 |  | 2.079 |  | 6.290 |  | 0.016 |  | 0.022 |  |
| time ✻ ocd\_vs\_hc |  | 0.010 |  | 1 |  | 0.010 |  | 0.031 |  | 0.860 |  | 1.103e -4 |  |
| Residuals |  | 15.207 |  | 46 |  | 0.331 |  |  |  |  |  |  |  |
| stim |  | 0.035 |  | 1 |  | 0.035 |  | 0.076 |  | 0.785 |  | 3.759e -4 |  |
| stim ✻ ocd\_vs\_hc |  | 2.615e -4 |  | 1 |  | 2.615e -4 |  | 5.589e -4 |  | 0.981 |  | 2.778e -6 |  |
| Residuals |  | 21.525 |  | 46 |  | 0.468 |  |  |  |  |  |  |  |
| time ✻ stim |  | 0.089 |  | 1 |  | 0.089 |  | 0.248 |  | 0.621 |  | 9.414e -4 |  |
| time ✻ stim ✻ ocd\_vs\_hc |  | 0.075 |  | 1 |  | 0.075 |  | 0.209 |  | 0.650 |  | 7.937e -4 |  |
| Residuals |  | 16.456 |  | 46 |  | 0.358 |  |  |  |  |  |  |  |
|  | | | | | | | | | | | | | |
|  |  |  |  |  |  |  |  |  |  |  |  |  |  |
| --- | --- | --- | --- | --- | --- | --- | --- | --- | --- | --- | --- | --- | --- |
| *Note.*  Type III Sum of Squares | | | | | | | | | | | | | |

| Between Subjects Effects | | | | | | | | | | | | | |
| --- | --- | --- | --- | --- | --- | --- | --- | --- | --- | --- | --- | --- | --- |
| Cases | | Sum of Squares | | df | | Mean Square | | F | | p | | η² | |
| ocd\_vs\_hc |  | 7.131e -4 |  | 1 |  | 7.131e -4 |  | 8.487e -4 |  | 0.977 |  | 7.576e -6 |  |
| Residuals |  | 38.650 |  | 46 |  | 0.840 |  |  |  |  |  |  |  |
|  | | | | | | | | | | | | | |
|  |  |  |  |  |  |  |  |  |  |  |  |  |  |
| --- | --- | --- | --- | --- | --- | --- | --- | --- | --- | --- | --- | --- | --- |
| *Note.*  Type III Sum of Squares | | | | | | | | | | | | | |

### Descriptives

| Descriptives | | | | | | | | | | | |
| --- | --- | --- | --- | --- | --- | --- | --- | --- | --- | --- | --- |
| time | | stim | | ocd\_vs\_hc | | Mean | | SD | | N | |
| 1 |  | min |  | 0 |  | -0.208 |  | 0.608 |  | 24 |  |
|  |  |  |  | 1 |  | -0.231 |  | 0.626 |  | 24 |  |
|  |  | shock |  | 0 |  | -0.234 |  | 0.695 |  | 24 |  |
|  |  |  |  | 1 |  | -0.173 |  | 0.747 |  | 24 |  |
| 2 |  | min |  | 0 |  | -0.398 |  | 0.831 |  | 24 |  |
|  |  |  |  | 1 |  | -0.371 |  | 0.725 |  | 24 |  |
|  |  | shock |  | 0 |  | -0.431 |  | 0.776 |  | 24 |  |
|  |  |  |  | 1 |  | -0.479 |  | 0.612 |  | 24 |  |
|  | | | | | | | | | | | |

#### Descriptives plots

##### ocd\_vs\_hc: 0

##### ocd\_vs\_hc: 1

## Table S34. Pupil dilation reinstatement (cs+air) frequentist ANOVAs

| Within Subjects Effects | | | | | | | | | | | | | |
| --- | --- | --- | --- | --- | --- | --- | --- | --- | --- | --- | --- | --- | --- |
| Cases | | Sum of Squares | | df | | Mean Square | | F | | p | | η² | |
| time |  | 0.455 |  | 1 |  | 0.455 |  | 1.931 |  | 0.171 |  | 0.005 |  |
| time ✻ ocd\_vs\_hc |  | 0.537 |  | 1 |  | 0.537 |  | 2.280 |  | 0.138 |  | 0.006 |  |
| Residuals |  | 10.828 |  | 46 |  | 0.235 |  |  |  |  |  |  |  |
| stim |  | 2.264 |  | 1 |  | 2.264 |  | 3.917 |  | 0.054 |  | 0.025 |  |
| stim ✻ ocd\_vs\_hc |  | 0.087 |  | 1 |  | 0.087 |  | 0.150 |  | 0.700 |  | 9.577e -4 |  |
| Residuals |  | 26.592 |  | 46 |  | 0.578 |  |  |  |  |  |  |  |
| time ✻ stim |  | 0.221 |  | 1 |  | 0.221 |  | 0.517 |  | 0.476 |  | 0.002 |  |
| time ✻ stim ✻ ocd\_vs\_hc |  | 0.817 |  | 1 |  | 0.817 |  | 1.910 |  | 0.174 |  | 0.009 |  |
| Residuals |  | 19.685 |  | 46 |  | 0.428 |  |  |  |  |  |  |  |
|  | | | | | | | | | | | | | |
|  |  |  |  |  |  |  |  |  |  |  |  |  |  |
| --- | --- | --- | --- | --- | --- | --- | --- | --- | --- | --- | --- | --- | --- |
| *Note.*  Type III Sum of Squares | | | | | | | | | | | | | |

| Between Subjects Effects | | | | | | | | | | | | | |
| --- | --- | --- | --- | --- | --- | --- | --- | --- | --- | --- | --- | --- | --- |
| Cases | | Sum of Squares | | df | | Mean Square | | F | | p | | η² | |
| ocd\_vs\_hc |  | 0.093 |  | 1 |  | 0.093 |  | 0.147 |  | 0.703 |  | 0.001 |  |
| Residuals |  | 29.104 |  | 46 |  | 0.633 |  |  |  |  |  |  |  |
|  | | | | | | | | | | | | | |
|  |  |  |  |  |  |  |  |  |  |  |  |  |  |
| --- | --- | --- | --- | --- | --- | --- | --- | --- | --- | --- | --- | --- | --- |
| *Note.*  Type III Sum of Squares | | | | | | | | | | | | | |

### Descriptives

| Descriptives | | | | | | | | | | | |
| --- | --- | --- | --- | --- | --- | --- | --- | --- | --- | --- | --- |
| time | | stim | | ocd\_vs\_hc | | Mean | | SD | | N | |
| 1 |  | min |  | 0 |  | -0.208 |  | 0.608 |  | 24 |  |
|  |  |  |  | 1 |  | -0.231 |  | 0.626 |  | 24 |  |
|  |  | shock |  | 0 |  | -0.231 |  | 0.685 |  | 24 |  |
|  |  |  |  | 1 |  | 0.091 |  | 0.718 |  | 24 |  |
| 2 |  | min |  | 0 |  | -0.398 |  | 0.831 |  | 24 |  |
|  |  |  |  | 1 |  | -0.371 |  | 0.725 |  | 24 |  |
|  |  | shock |  | 0 |  | -0.025 |  | 0.729 |  | 24 |  |
|  |  |  |  | 1 |  | -0.174 |  | 0.506 |  | 24 |  |
|  | | | | | | | | | | | |

#### Descriptives plots

##### ocd\_vs\_hc: 0

##### ocd\_vs\_hc: 1

## Table S35. Startle recall (cs+shock) frequentist ANOVAs

| Within Subjects Effects | | | | | | | | | | | | | |
| --- | --- | --- | --- | --- | --- | --- | --- | --- | --- | --- | --- | --- | --- |
| Cases | | Sum of Squares | | df | | Mean Square | | F | | p | | η² | |
| time |  | 20.908 |  | 1 |  | 20.908 |  | 52.299 |  | < .001 |  | 0.107 |  |
| time ✻ ocd\_vs\_hc |  | 0.032 |  | 1 |  | 0.032 |  | 0.080 |  | 0.779 |  | 1.633e -4 |  |
| Residuals |  | 25.186 |  | 63 |  | 0.400 |  |  |  |  |  |  |  |
| stim |  | 6.209 | ᵃ | 2 | ᵃ | 3.104 | ᵃ | 9.461 | ᵃ | < .001 | ᵃ | 0.032 |  |
| stim ✻ ocd\_vs\_hc |  | 0.495 | ᵃ | 2 | ᵃ | 0.247 | ᵃ | 0.754 | ᵃ | 0.473 | ᵃ | 0.003 |  |
| Residuals |  | 41.345 |  | 126 |  | 0.328 |  |  |  |  |  |  |  |
| time ✻ stim |  | 2.141 |  | 2 |  | 1.071 |  | 3.597 |  | 0.030 |  | 0.011 |  |
| time ✻ stim ✻ ocd\_vs\_hc |  | 0.702 |  | 2 |  | 0.351 |  | 1.180 |  | 0.311 |  | 0.004 |  |
| Residuals |  | 37.508 |  | 126 |  | 0.298 |  |  |  |  |  |  |  |
|  | | | | | | | | | | | | | |
|  |  |  |  |  |  |  |  |  |  |  |  |  |  |
| --- | --- | --- | --- | --- | --- | --- | --- | --- | --- | --- | --- | --- | --- |
| *Note.*  Type III Sum of Squares | | | | | | | | | | | | | |
| ᵃ Mauchly's test of sphericity indicates that the assumption of sphericity is violated (p < .05). | | | | | | | | | | | | | |

| Between Subjects Effects | | | | | | | | | | | | | |
| --- | --- | --- | --- | --- | --- | --- | --- | --- | --- | --- | --- | --- | --- |
| Cases | | Sum of Squares | | df | | Mean Square | | F | | p | | η² | |
| ocd\_vs\_hc |  | 0.219 |  | 1 |  | 0.219 |  | 0.230 |  | 0.633 |  | 0.001 |  |
| Residuals |  | 59.836 |  | 63 |  | 0.950 |  |  |  |  |  |  |  |
|  | | | | | | | | | | | | | |
|  |  |  |  |  |  |  |  |  |  |  |  |  |  |
| --- | --- | --- | --- | --- | --- | --- | --- | --- | --- | --- | --- | --- | --- |
| *Note.*  Type III Sum of Squares | | | | | | | | | | | | | |

### Descriptives

| Descriptives | | | | | | | | | | | |
| --- | --- | --- | --- | --- | --- | --- | --- | --- | --- | --- | --- |
| time | | stim | | ocd\_vs\_hc | | Mean | | SD | | N | |
| 1 |  | iti |  | 0 |  | 0.056 |  | 0.594 |  | 40 |  |
|  |  |  |  | 1 |  | 0.208 |  | 1.146 |  | 25 |  |
|  |  | min |  | 0 |  | 0.461 |  | 0.701 |  | 40 |  |
|  |  |  |  | 1 |  | 0.233 |  | 0.591 |  | 25 |  |
|  |  | shock |  | 0 |  | 0.432 |  | 0.591 |  | 40 |  |
|  |  |  |  | 1 |  | 0.307 |  | 0.632 |  | 25 |  |
| 2 |  | iti |  | 0 |  | -0.296 |  | 0.525 |  | 40 |  |
|  |  |  |  | 1 |  | -0.343 |  | 0.618 |  | 25 |  |
|  |  | min |  | 0 |  | -0.321 |  | 0.559 |  | 40 |  |
|  |  |  |  | 1 |  | -0.331 |  | 0.448 |  | 25 |  |
|  |  | shock |  | 0 |  | 0.084 |  | 0.649 |  | 40 |  |
|  |  |  |  | 1 |  | 0.050 |  | 0.776 |  | 25 |  |
|  | | | | | | | | | | | |

#### Descriptives plots

##### ocd\_vs\_hc: 0

##### ocd\_vs\_hc: 1

## Table S36. Startle recall (cs+air) frequentist ANOVAs

| Within Subjects Effects | | | | | | | | | | | | | |
| --- | --- | --- | --- | --- | --- | --- | --- | --- | --- | --- | --- | --- | --- |
| Cases | | Sum of Squares | | df | | Mean Square | | F | | p | | η² | |
| time |  | 28.074 |  | 1 |  | 28.074 |  | 62.288 |  | < .001 |  | 0.137 |  |
| time ✻ ocd\_vs\_hc |  | 0.448 |  | 1 |  | 0.448 |  | 0.994 |  | 0.323 |  | 0.002 |  |
| Residuals |  | 27.944 |  | 62 |  | 0.451 |  |  |  |  |  |  |  |
| stim |  | 6.137 |  | 2 |  | 3.068 |  | 8.997 |  | < .001 |  | 0.030 |  |
| stim ✻ ocd\_vs\_hc |  | 1.073 |  | 2 |  | 0.537 |  | 1.574 |  | 0.211 |  | 0.005 |  |
| Residuals |  | 42.290 |  | 124 |  | 0.341 |  |  |  |  |  |  |  |
| time ✻ stim |  | 1.385 |  | 2 |  | 0.693 |  | 2.669 |  | 0.073 |  | 0.007 |  |
| time ✻ stim ✻ ocd\_vs\_hc |  | 0.516 |  | 2 |  | 0.258 |  | 0.994 |  | 0.373 |  | 0.003 |  |
| Residuals |  | 32.178 |  | 124 |  | 0.259 |  |  |  |  |  |  |  |
|  | | | | | | | | | | | | | |
|  |  |  |  |  |  |  |  |  |  |  |  |  |  |
| --- | --- | --- | --- | --- | --- | --- | --- | --- | --- | --- | --- | --- | --- |
| *Note.*  Type III Sum of Squares | | | | | | | | | | | | | |
| ᵃ Mauchly's test of sphericity indicates that the assumption of sphericity is violated (p < .05). | | | | | | | | | | | | | |

| Between Subjects Effects | | | | | | | | | | | | | |
| --- | --- | --- | --- | --- | --- | --- | --- | --- | --- | --- | --- | --- | --- |
| Cases | | Sum of Squares | | df | | Mean Square | | F | | p | | η² | |
| ocd\_vs\_hc |  | 1.513 |  | 1 |  | 1.513 |  | 1.493 |  | 0.226 |  | 0.007 |  |
| Residuals |  | 62.841 |  | 62 |  | 1.014 |  |  |  |  |  |  |  |
|  | | | | | | | | | | | | | |
|  |  |  |  |  |  |  |  |  |  |  |  |  |  |
| --- | --- | --- | --- | --- | --- | --- | --- | --- | --- | --- | --- | --- | --- |
| *Note.*  Type III Sum of Squares | | | | | | | | | | | | | |

### Descriptives

| Descriptives | | | | | | | | | | | |
| --- | --- | --- | --- | --- | --- | --- | --- | --- | --- | --- | --- |
| time | | stim | | ocd\_vs\_hc | | Mean | | SD | | N | |
| 1 |  | air |  | 0 |  | 0.707 |  | 0.980 |  | 40 |  |
|  |  |  |  | 1 |  | 0.294 |  | 0.648 |  | 24 |  |
|  |  | iti |  | 0 |  | 0.056 |  | 0.594 |  | 40 |  |
|  |  |  |  | 1 |  | 0.083 |  | 0.980 |  | 24 |  |
|  |  | min |  | 0 |  | 0.461 |  | 0.701 |  | 40 |  |
|  |  |  |  | 1 |  | 0.248 |  | 0.600 |  | 24 |  |
| 2 |  | air |  | 0 |  | -0.045 |  | 0.584 |  | 40 |  |
|  |  |  |  | 1 |  | -0.177 |  | 0.450 |  | 24 |  |
|  |  | iti |  | 0 |  | -0.296 |  | 0.525 |  | 40 |  |
|  |  |  |  | 1 |  | -0.339 |  | 0.631 |  | 24 |  |
|  |  | min |  | 0 |  | -0.321 |  | 0.559 |  | 40 |  |
|  |  |  |  | 1 |  | -0.324 |  | 0.456 |  | 24 |  |
|  | | | | | | | | | | | |

#### Descriptives plots

##### ocd\_vs\_hc: 0

##### ocd\_vs\_hc: 1

## Table S37. Startle reinstatement (cs+shock) frequentist ANOVAs

| Within Subjects Effects | | | | | | | | | | | | | |
| --- | --- | --- | --- | --- | --- | --- | --- | --- | --- | --- | --- | --- | --- |
| Cases | | Sum of Squares | | df | | Mean Square | | F | | p | | η² | |
| time |  | 19.913 |  | 1 |  | 19.913 |  | 39.618 |  | < .001 |  | 0.190 |  |
| time ✻ ocd\_vs\_hc |  | 1.137 |  | 1 |  | 1.137 |  | 2.263 |  | 0.139 |  | 0.011 |  |
| Residuals |  | 23.623 |  | 47 |  | 0.503 |  |  |  |  |  |  |  |
| stim |  | 2.667 |  | 1 |  | 2.667 |  | 11.512 |  | 0.001 |  | 0.025 |  |
| stim ✻ ocd\_vs\_hc |  | 0.018 |  | 1 |  | 0.018 |  | 0.076 |  | 0.785 |  | 1.674e -4 |  |
| Residuals |  | 10.887 |  | 47 |  | 0.232 |  |  |  |  |  |  |  |
| time ✻ stim |  | 0.328 |  | 1 |  | 0.328 |  | 1.960 |  | 0.168 |  | 0.003 |  |
| time ✻ stim ✻ ocd\_vs\_hc |  | 0.168 |  | 1 |  | 0.168 |  | 1.004 |  | 0.321 |  | 0.002 |  |
| Residuals |  | 7.859 |  | 47 |  | 0.167 |  |  |  |  |  |  |  |
|  | | | | | | | | | | | | | |
|  |  |  |  |  |  |  |  |  |  |  |  |  |  |
| --- | --- | --- | --- | --- | --- | --- | --- | --- | --- | --- | --- | --- | --- |
| *Note.*  Type III Sum of Squares | | | | | | | | | | | | | |

| Between Subjects Effects | | | | | | | | | | | | | |
| --- | --- | --- | --- | --- | --- | --- | --- | --- | --- | --- | --- | --- | --- |
| Cases | | Sum of Squares | | df | | Mean Square | | F | | p | | η² | |
| ocd\_vs\_hc |  | 0.084 |  | 1 |  | 0.084 |  | 0.105 |  | 0.748 |  | 8.077e -4 |  |
| Residuals |  | 37.928 |  | 47 |  | 0.807 |  |  |  |  |  |  |  |
|  | | | | | | | | | | | | | |
|  |  |  |  |  |  |  |  |  |  |  |  |  |  |
| --- | --- | --- | --- | --- | --- | --- | --- | --- | --- | --- | --- | --- | --- |
| *Note.*  Type III Sum of Squares | | | | | | | | | | | | | |

### Descriptives

| Descriptives | | | | | | | | | | | |
| --- | --- | --- | --- | --- | --- | --- | --- | --- | --- | --- | --- |
| time | | stim | | ocd\_vs\_hc | | Mean | | SD | | N | |
| 1 |  | min |  | 0 |  | 0.010 |  | 0.836 |  | 28 |  |
|  |  |  |  | 1 |  | -0.146 |  | 0.651 |  | 21 |  |
|  |  | shock |  | 0 |  | 0.368 |  | 0.986 |  | 28 |  |
|  |  |  |  | 1 |  | 0.132 |  | 0.770 |  | 21 |  |
| 2 |  | min |  | 0 |  | -0.647 |  | 0.417 |  | 28 |  |
|  |  |  |  | 1 |  | -0.613 |  | 0.294 |  | 21 |  |
|  |  | shock |  | 0 |  | -0.572 |  | 0.373 |  | 28 |  |
|  |  |  |  | 1 |  | -0.382 |  | 0.486 |  | 21 |  |
|  | | | | | | | | | | | |

#### Descriptives plots

##### ocd\_vs\_hc: 0

##### ocd\_vs\_hc: 1

## Table S38. Startle reinstatement (cs+air) frequentist ANOVAs

| Within Subjects Effects | | | | | | | | | | | | | |
| --- | --- | --- | --- | --- | --- | --- | --- | --- | --- | --- | --- | --- | --- |
| Cases | | Sum of Squares | | df | | Mean Square | | F | | p | | η² | |
| time |  | 12.167 |  | 1 |  | 12.167 |  | 28.804 |  | < .001 |  | 0.126 |  |
| time ✻ ocd\_vs\_hc |  | 0.554 |  | 1 |  | 0.554 |  | 1.310 |  | 0.259 |  | 0.006 |  |
| Residuals |  | 18.163 |  | 43 |  | 0.422 |  |  |  |  |  |  |  |
| stim |  | 2.160 |  | 1 |  | 2.160 |  | 7.838 |  | 0.008 |  | 0.022 |  |
| stim ✻ ocd\_vs\_hc |  | 0.055 |  | 1 |  | 0.055 |  | 0.199 |  | 0.658 |  | 5.679e -4 |  |
| Residuals |  | 11.852 |  | 43 |  | 0.276 |  |  |  |  |  |  |  |
| time ✻ stim |  | 0.186 |  | 1 |  | 0.186 |  | 0.916 |  | 0.344 |  | 0.002 |  |
| time ✻ stim ✻ ocd\_vs\_hc |  | 0.089 |  | 1 |  | 0.089 |  | 0.438 |  | 0.511 |  | 9.242e -4 |  |
| Residuals |  | 8.743 |  | 43 |  | 0.203 |  |  |  |  |  |  |  |
|  | | | | | | | | | | | | | |
|  |  |  |  |  |  |  |  |  |  |  |  |  |  |
| --- | --- | --- | --- | --- | --- | --- | --- | --- | --- | --- | --- | --- | --- |
| *Note.*  Type III Sum of Squares | | | | | | | | | | | | | |

| Between Subjects Effects | | | | | | | | | | | | | |
| --- | --- | --- | --- | --- | --- | --- | --- | --- | --- | --- | --- | --- | --- |
| Cases | | Sum of Squares | | df | | Mean Square | | F | | p | | η² | |
| ocd\_vs\_hc |  | 0.865 |  | 1 |  | 0.865 |  | 0.894 |  | 0.350 |  | 0.009 |  |
| Residuals |  | 41.599 |  | 43 |  | 0.967 |  |  |  |  |  |  |  |
|  | | | | | | | | | | | | | |
|  |  |  |  |  |  |  |  |  |  |  |  |  |  |
| --- | --- | --- | --- | --- | --- | --- | --- | --- | --- | --- | --- | --- | --- |
| *Note.*  Type III Sum of Squares | | | | | | | | | | | | | |

### Descriptives

| Descriptives | | | | | | | | | | | |
| --- | --- | --- | --- | --- | --- | --- | --- | --- | --- | --- | --- |
| time | | stim | | ocd\_vs\_hc | | Mean | | SD | | N | |
| 1 |  | air |  | 0 |  | 0.283 |  | 0.977 |  | 24 |  |
|  |  |  |  | 1 |  | -0.046 |  | 0.845 |  | 21 |  |
|  |  | min |  | 0 |  | 0.049 |  | 0.866 |  | 24 |  |
|  |  |  |  | 1 |  | -0.122 |  | 0.645 |  | 21 |  |
| 2 |  | air |  | 0 |  | -0.329 |  | 0.480 |  | 24 |  |
|  |  |  |  | 1 |  | -0.347 |  | 0.592 |  | 21 |  |
|  |  | min |  | 0 |  | -0.604 |  | 0.435 |  | 24 |  |
|  |  |  |  | 1 |  | -0.641 |  | 0.307 |  | 21 |  |
|  | | | | | | | | | | | |

#### Descriptives plots

##### ocd\_vs\_hc: 0

##### ocd\_vs\_hc: 1

## Table S39. Subjective shock expectancy frequentist ANOVAs

| Within Subjects Effects | | | | | | | | | | | | | |
| --- | --- | --- | --- | --- | --- | --- | --- | --- | --- | --- | --- | --- | --- |
| Cases | | Sum of Squares | | df | | Mean Square | | F | | p | | η² | |
| time |  | 1161.486 | ᵃ | 2 | ᵃ | 580.743 | ᵃ | 239.123 | ᵃ | < .001 | ᵃ | 0.377 |  |
| time ✻ ocd\_vs\_hc |  | 1.795 | ᵃ | 2 | ᵃ | 0.897 | ᵃ | 0.370 | ᵃ | 0.692 | ᵃ | 5.830e -4 |  |
| Residuals |  | 325.437 |  | 134 |  | 2.429 |  |  |  |  |  |  |  |
| stim |  | 140.512 |  | 1 |  | 140.512 |  | 29.976 |  | < .001 |  | 0.046 |  |
| stim ✻ ocd\_vs\_hc |  | 1.923 |  | 1 |  | 1.923 |  | 0.410 |  | 0.524 |  | 6.246e -4 |  |
| Residuals |  | 314.067 |  | 67 |  | 4.688 |  |  |  |  |  |  |  |
| time ✻ stim |  | 136.967 | ᵃ | 2 | ᵃ | 68.483 | ᵃ | 18.708 | ᵃ | < .001 | ᵃ | 0.044 |  |
| time ✻ stim ✻ ocd\_vs\_hc |  | 1.411 | ᵃ | 2 | ᵃ | 0.706 | ᵃ | 0.193 | ᵃ | 0.825 | ᵃ | 4.584e -4 |  |
| Residuals |  | 490.526 |  | 134 |  | 3.661 |  |  |  |  |  |  |  |
|  | | | | | | | | | | | | | |
|  |  |  |  |  |  |  |  |  |  |  |  |  |  |
| --- | --- | --- | --- | --- | --- | --- | --- | --- | --- | --- | --- | --- | --- |
| *Note.*  Type III Sum of Squares | | | | | | | | | | | | | |
| ᵃ Mauchly's test of sphericity indicates that the assumption of sphericity is violated (p < .05). | | | | | | | | | | | | | |

| Between Subjects Effects | | | | | | | | | | | | | |
| --- | --- | --- | --- | --- | --- | --- | --- | --- | --- | --- | --- | --- | --- |
| Cases | | Sum of Squares | | df | | Mean Square | | F | | p | | η² | |
| ocd\_vs\_hc |  | 4.077 |  | 1 |  | 4.077 |  | 0.546 |  | 0.463 |  | 0.001 |  |
| Residuals |  | 500.416 |  | 67 |  | 7.469 |  |  |  |  |  |  |  |
|  | | | | | | | | | | | | | |
|  |  |  |  |  |  |  |  |  |  |  |  |  |  |
| --- | --- | --- | --- | --- | --- | --- | --- | --- | --- | --- | --- | --- | --- |
| *Note.*  Type III Sum of Squares | | | | | | | | | | | | | |

### Descriptives

| Descriptives | | | | | | | | | | | |
| --- | --- | --- | --- | --- | --- | --- | --- | --- | --- | --- | --- |
| time | | stim | | ocd\_vs\_hc | | Mean | | SD | | N | |
| 1 |  | air |  | 0 |  | 5.732 |  | 3.202 |  | 41 |  |
|  |  |  |  | 1 |  | 5.036 |  | 3.214 |  | 28 |  |
|  |  | min |  | 0 |  | 2.585 |  | 2.889 |  | 41 |  |
|  |  |  |  | 1 |  | 2.500 |  | 2.333 |  | 28 |  |
| 2 |  | air |  | 0 |  | 0.732 |  | 2.013 |  | 41 |  |
|  |  |  |  | 1 |  | 0.571 |  | 1.451 |  | 28 |  |
|  |  | min |  | 0 |  | 0.268 |  | 1.001 |  | 41 |  |
|  |  |  |  | 1 |  | 0.179 |  | 0.772 |  | 28 |  |
| 3 |  | air |  | 0 |  | 0.488 |  | 1.762 |  | 41 |  |
|  |  |  |  | 1 |  | 0.321 |  | 1.156 |  | 28 |  |
|  |  | min |  | 0 |  | 0.122 |  | 0.640 |  | 41 |  |
|  |  |  |  | 1 |  | 0.107 |  | 0.567 |  | 28 |  |
|  | | | | | | | | | | | |

#### Descriptives plots

##### ocd\_vs\_hc: 0

##### ocd\_vs\_hc: 1

## Table S40. Subjective airblast expectancy frequentist ANOVAs

| Within Subjects Effects | | | | | | | | | | | | | |
| --- | --- | --- | --- | --- | --- | --- | --- | --- | --- | --- | --- | --- | --- |
| Cases | | Sum of Squares | | df | | Mean Square | | F | | p | | η² | |
| time |  | 1162.291 | ᵃ | 2 | ᵃ | 581.145 | ᵃ | 172.255 | ᵃ | < .001 | ᵃ | 0.378 |  |
| time ✻ ocd\_vs\_hc |  | 3.508 | ᵃ | 2 | ᵃ | 1.754 | ᵃ | 0.520 | ᵃ | 0.596 | ᵃ | 0.001 |  |
| Residuals |  | 452.081 |  | 134 |  | 3.374 |  |  |  |  |  |  |  |
| stim |  | 171.111 |  | 1 |  | 171.111 |  | 49.181 |  | < .001 |  | 0.056 |  |
| stim ✻ ocd\_vs\_hc |  | 8.416e -6 |  | 1 |  | 8.416e -6 |  | 2.419e -6 |  | 0.999 |  | 2.735e -9 |  |
| Residuals |  | 233.106 |  | 67 |  | 3.479 |  |  |  |  |  |  |  |
| time ✻ stim |  | 228.351 | ᵃ | 2 | ᵃ | 114.175 | ᵃ | 39.835 | ᵃ | < .001 | ᵃ | 0.074 |  |
| time ✻ stim ✻ ocd\_vs\_hc |  | 0.621 | ᵃ | 2 | ᵃ | 0.311 | ᵃ | 0.108 | ᵃ | 0.897 | ᵃ | 2.018e -4 |  |
| Residuals |  | 384.070 |  | 134 |  | 2.866 |  |  |  |  |  |  |  |
|  | | | | | | | | | | | | | |
|  |  |  |  |  |  |  |  |  |  |  |  |  |  |
| --- | --- | --- | --- | --- | --- | --- | --- | --- | --- | --- | --- | --- | --- |
| *Note.*  Type III Sum of Squares | | | | | | | | | | | | | |
| ᵃ Mauchly's test of sphericity indicates that the assumption of sphericity is violated (p < .05). | | | | | | | | | | | | | |

| Between Subjects Effects | | | | | | | | | | | | | |
| --- | --- | --- | --- | --- | --- | --- | --- | --- | --- | --- | --- | --- | --- |
| Cases | | Sum of Squares | | df | | Mean Square | | F | | p | | η² | |
| ocd\_vs\_hc |  | 0.004 |  | 1 |  | 0.004 |  | 6.740e -4 |  | 0.979 |  | 1.447e -6 |  |
| Residuals |  | 442.551 |  | 67 |  | 6.605 |  |  |  |  |  |  |  |
|  | | | | | | | | | | | | | |
|  |  |  |  |  |  |  |  |  |  |  |  |  |  |
| --- | --- | --- | --- | --- | --- | --- | --- | --- | --- | --- | --- | --- | --- |
| *Note.*  Type III Sum of Squares | | | | | | | | | | | | | |

### Descriptives

| Descriptives | | | | | | | | | | | |
| --- | --- | --- | --- | --- | --- | --- | --- | --- | --- | --- | --- |
| time | | stim | | ocd\_vs\_hc | | Mean | | SD | | N | |
| 1 |  | air |  | 0 |  | 5.634 |  | 2.888 |  | 41 |  |
|  |  |  |  | 1 |  | 5.857 |  | 2.578 |  | 28 |  |
|  |  | min |  | 0 |  | 2.171 |  | 2.376 |  | 41 |  |
|  |  |  |  | 1 |  | 2.429 |  | 2.441 |  | 28 |  |
| 2 |  | air |  | 0 |  | 0.585 |  | 1.565 |  | 41 |  |
|  |  |  |  | 1 |  | 0.643 |  | 1.496 |  | 28 |  |
|  |  | min |  | 0 |  | 0.366 |  | 1.639 |  | 41 |  |
|  |  |  |  | 1 |  | 0.214 |  | 0.833 |  | 28 |  |
| 3 |  | air |  | 0 |  | 0.585 |  | 1.949 |  | 41 |  |
|  |  |  |  | 1 |  | 0.286 |  | 1.150 |  | 28 |  |
|  |  | min |  | 0 |  | 0.341 |  | 1.622 |  | 41 |  |
|  |  |  |  | 1 |  | 0.214 |  | 1.134 |  | 28 |  |
|  | | | | | | | | | | | |

#### Descriptives plots

##### ocd\_vs\_hc: 0

##### ocd\_vs\_hc: 1

## Table S41. Subjective shock expectancy frequentist ANOVAs

| Within Subjects Effects | | | | | | | | | | | | | |
| --- | --- | --- | --- | --- | --- | --- | --- | --- | --- | --- | --- | --- | --- |
| Cases | | Sum of Squares | | df | | Mean Square | | F | | p | | η² | |
| time |  | 0.983 |  | 1 |  | 0.983 |  | 3.928 |  | 0.052 |  | 0.003 |  |
| time ✻ ocd\_vs\_hc |  | 4.408e -4 |  | 1 |  | 4.408e -4 |  | 0.002 |  | 0.967 |  | 1.160e -6 |  |
| Residuals |  | 13.763 |  | 55 |  | 0.250 |  |  |  |  |  |  |  |
| stim |  | 0.233 |  | 1 |  | 0.233 |  | 0.524 |  | 0.472 |  | 6.134e -4 |  |
| stim ✻ ocd\_vs\_hc |  | 0.058 |  | 1 |  | 0.058 |  | 0.130 |  | 0.720 |  | 1.519e -4 |  |
| Residuals |  | 24.477 |  | 55 |  | 0.445 |  |  |  |  |  |  |  |
| time ✻ stim |  | 0.114 |  | 1 |  | 0.114 |  | 0.430 |  | 0.515 |  | 3.010e -4 |  |
| time ✻ stim ✻ ocd\_vs\_hc |  | 0.009 |  | 1 |  | 0.009 |  | 0.034 |  | 0.854 |  | 2.406e -5 |  |
| Residuals |  | 14.631 |  | 55 |  | 0.266 |  |  |  |  |  |  |  |
|  | | | | | | | | | | | | | |
|  |  |  |  |  |  |  |  |  |  |  |  |  |  |
| --- | --- | --- | --- | --- | --- | --- | --- | --- | --- | --- | --- | --- | --- |
| *Note.*  Type III Sum of Squares | | | | | | | | | | | | | |

| Between Subjects Effects | | | | | | | | | | | | | |
| --- | --- | --- | --- | --- | --- | --- | --- | --- | --- | --- | --- | --- | --- |
| Cases | | Sum of Squares | | df | | Mean Square | | F | | p | | η² | |
| ocd\_vs\_hc |  | 3.704 |  | 1 |  | 3.704 |  | 0.632 |  | 0.430 |  | 0.010 |  |
| Residuals |  | 322.165 |  | 55 |  | 5.858 |  |  |  |  |  |  |  |
|  | | | | | | | | | | | | | |
|  |  |  |  |  |  |  |  |  |  |  |  |  |  |
| --- | --- | --- | --- | --- | --- | --- | --- | --- | --- | --- | --- | --- | --- |
| *Note.*  Type III Sum of Squares | | | | | | | | | | | | | |

### Descriptives

| Descriptives | | | | | | | | | | | |
| --- | --- | --- | --- | --- | --- | --- | --- | --- | --- | --- | --- |
| time | | stim | | ocd\_vs\_hc | | Mean | | SD | | N | |
| 1 |  | min |  | 0 |  | 0.452 |  | 1.823 |  | 31 |  |
|  |  |  |  | 1 |  | 0.154 |  | 0.784 |  | 26 |  |
|  |  | shock |  | 0 |  | 0.516 |  | 1.913 |  | 31 |  |
|  |  |  |  | 1 |  | 0.308 |  | 1.192 |  | 26 |  |
| 2 |  | min |  | 0 |  | 0.355 |  | 1.380 |  | 31 |  |
|  |  |  |  | 1 |  | 0.077 |  | 0.392 |  | 26 |  |
|  |  | shock |  | 0 |  | 0.355 |  | 1.279 |  | 31 |  |
|  |  |  |  | 1 |  | 0.115 |  | 0.431 |  | 26 |  |
|  | | | | | | | | | | | |

#### Descriptives plots

##### ocd\_vs\_hc: 0

##### ocd\_vs\_hc: 1

## Table 42. Subjective airblast expectancy frequentist ANOVAs

| Within Subjects Effects | | | | | | | | | | | | | |
| --- | --- | --- | --- | --- | --- | --- | --- | --- | --- | --- | --- | --- | --- |
| Cases | | Sum of Squares | | df | | Mean Square | | F | | p | | η² | |
| time |  | 0.290 |  | 1 |  | 0.290 |  | 0.255 |  | 0.616 |  | 5.568e -4 |  |
| time ✻ ocd\_vs\_hc |  | 2.659 |  | 1 |  | 2.659 |  | 2.333 |  | 0.132 |  | 0.005 |  |
| Residuals |  | 62.683 |  | 55 |  | 1.140 |  |  |  |  |  |  |  |
| stim |  | 0.506 |  | 1 |  | 0.506 |  | 0.509 |  | 0.479 |  | 9.706e -4 |  |
| stim ✻ ocd\_vs\_hc |  | 0.348 |  | 1 |  | 0.348 |  | 0.350 |  | 0.556 |  | 6.678e -4 |  |
| Residuals |  | 54.713 |  | 55 |  | 0.995 |  |  |  |  |  |  |  |
| time ✻ stim |  | 2.751 |  | 1 |  | 2.751 |  | 3.019 |  | 0.088 |  | 0.005 |  |
| time ✻ stim ✻ ocd\_vs\_hc |  | 0.418 |  | 1 |  | 0.418 |  | 0.458 |  | 0.501 |  | 8.006e -4 |  |
| Residuals |  | 50.118 |  | 55 |  | 0.911 |  |  |  |  |  |  |  |
|  | | | | | | | | | | | | | |
|  |  |  |  |  |  |  |  |  |  |  |  |  |  |
| --- | --- | --- | --- | --- | --- | --- | --- | --- | --- | --- | --- | --- | --- |
| *Note.*  Type III Sum of Squares | | | | | | | | | | | | | |

| Between Subjects Effects | | | | | | | | | | | | | |
| --- | --- | --- | --- | --- | --- | --- | --- | --- | --- | --- | --- | --- | --- |
| Cases | | Sum of Squares | | df | | Mean Square | | F | | p | | η² | |
| ocd\_vs\_hc |  | 5.874 |  | 1 |  | 5.874 |  | 0.947 |  | 0.335 |  | 0.011 |  |
| Residuals |  | 341.187 |  | 55 |  | 6.203 |  |  |  |  |  |  |  |
|  | | | | | | | | | | | | | |
|  |  |  |  |  |  |  |  |  |  |  |  |  |  |
| --- | --- | --- | --- | --- | --- | --- | --- | --- | --- | --- | --- | --- | --- |
| *Note.*  Type III Sum of Squares | | | | | | | | | | | | | |

### Descriptives

| Descriptives | | | | | | | | | | | |
| --- | --- | --- | --- | --- | --- | --- | --- | --- | --- | --- | --- |
| time | | stim | | ocd\_vs\_hc | | Mean | | SD | | N | |
| 1 |  | air |  | 0 |  | 0.613 |  | 2.028 |  | 31 |  |
|  |  |  |  | 1 |  | 0.500 |  | 1.556 |  | 26 |  |
|  |  | min |  | 0 |  | 0.290 |  | 0.864 |  | 31 |  |
|  |  |  |  | 1 |  | 0.192 |  | 0.801 |  | 26 |  |
| 2 |  | air |  | 0 |  | 0.452 |  | 1.748 |  | 31 |  |
|  |  |  |  | 1 |  | 0.077 |  | 0.272 |  | 26 |  |
|  |  | min |  | 0 |  | 0.742 |  | 2.529 |  | 31 |  |
|  |  |  |  | 1 |  | 0.038 |  | 0.196 |  | 26 |  |
|  | | | | | | | | | | | |

#### Descriptives plots

##### ocd\_vs\_hc: 0

##### ocd\_vs\_hc: 1
